# Secreted KIAA1199 promotes the progression of rheumatoid arthritis by mediating hyaluronic acid degradation in an ANXA1-dependent manner

**DOI:** 10.1038/s41419-021-03393-5

**Published:** 2021-01-20

**Authors:** Wei Zhang, Guoyu Yin, Heping Zhao, Hanzhi Ling, Zhen Xie, Chipeng Xiao, Yan Chen, Yufan Lin, Tao Jiang, Shengwei Jin, Jianguang Wang, Xinyu Yang

**Affiliations:** 1 Department of Biochemistry, School of Basic Medical Sciences, Wenzhou Medical University, Wenzhou, China; 2 Department of Anesthesia and Critical Care, The Second Affiliated Hospital of Wenzhou Medical University, Wenzhou, China; 3 Department of Medicinal Chemistry, School of Pharmaceutical Sciences, Wenzhou Medical University, Wenzhou, China

**Keywords:** Extracellular signalling molecules, Rheumatoid arthritis

## Abstract

In inflamed joints, enhanced hyaluronic acid (HA) degradation is closely related to the pathogenesis of rheumatoid arthritis (RA). KIAA1199 has been identified as a hyaladherin that mediates the intracellular degradation of HA, but its extracellular function remains unclear. In this study, we found that the serum and synovial levels of secreted KIAA1199 (sKIAA1199) and low-molecular-weight HA (LMW-HA, MW < 100 kDa) in RA patients were significantly increased, and the positive correlation between them was shown for the first time. Of note, treatment with anti-KIAA1199 mAb effectively alleviated the severity of arthritis and reduced serum LMW-HA levels and cytokine secretion in collagen-induced arthritis (CIA) mice. In vitro, sKIAA1199 was shown to mediate exogenous HA degradation by attaching to the cell membrane of RA fibroblast-like synoviosytes (RA FLS). Furthermore, the HA-degrading activity of sKIAA1199 depended largely on its adhesion to the membrane, which was achieved by its G8 domain binding to ANXA1. In vivo, *kiaa1199*-KO mice exhibited greater resistance to collagen-induced arthritis. Interestingly, this resistance could be partially reversed by intra-articular injection of vectors encoding full-length KIAA1199 instead of G8-deleted KIAA119 mutant, which further confirmed the indispensable role of G8 domain in KIAA1199 involvement in RA pathological processes. Mechanically, the activation of NF-κB by interleukin-6 (IL-6) through PI3K/Akt signaling is suggested to be the main pathway to induce KIAA1199 expression in RA FLS. In conclusion, our study supported the contribution of sKIAA1199 to RA pathogenesis, providing a new therapeutic target for RA by blocking sKIAA1199-mediated HA degradation.

## Introduction

Rheumatoid arthritis (RA) is a chronic autoimmune disease characterized by pain, swelling, bone and cartilage destruction, and ultimately leading to joint disability^1^. Imbalance of hyaluronic acid (HA) metabolism in synovial tissues is tightly related to the pathogenesis of RA^2,3^. Native HA (n-HA) with high molecular weight (HMW, MW > 1000 kDa) in synovial fluid normally functions as a lubricant and a barrier to cytokines. Under inflammatory conditions, however, excessive low-molecular-weight HA (LMW-HA, MW < 100 kD) produced by intensified n-HA degradation exerts pro-inflammatory and pro-angiogenic effects^4,5^. Although HA size is thought to be largely determined by its catabolism^6^, the exact mechanism of HA degradation in inflamed joints is still not fully understood.

Hyaluronidases (HYALs) and oxygen-derived free radicals were generally accepted as two main causes of LMW-HA accumulation in synovial fluid of RA patients^2^. However, a novel pathway of KIAA1199-mediated HA degradation in dermis of the skin and arthritic synovium was first revealed a few years ago by Yoshida et al.^7^. Just before that, our proteomics results also showed that the protein level of KIAA1199 was abnormally elevated in RA fibroblast-like synoviocytes (FLS)^8^. In vitro functional experiments further demonstrated that circulating KIAA1199 could be a potential biomarker for RA diagnosis^9^. Nonetheless, in vivo evidence for the effect of KIAA1199 on the progression of RA is still lacking.

KIAA1199, also known as CEMIP (cell migration inducing protein), is a multi-domain protein with a molecular weight of ~ 153 kDa encoded by the *CEMIP* gene located on chromosome 15q25.1. Structurally, the newly translated KIAA1199 has an N-terminal signal peptide required for its intracellular transport, HA-degrading activity, and even secretion^10^. Although its clinical diagnostic value has been evaluated in several diseases including RA^9,11^, the function of secreted KIAA1199 (sKIAA1199) remains elusive. A few studies have reported that HMW-HA could not be degraded in the KIAA1199-rich conditioned medium^7,10^. In contrast, a recent study suggested that increased extracellular KIAA1199 resulted in the accumulation of HA fragments in inflamed colon of patients with Crohn’s disease^12^. Thus, more research is needed to clarify whether sKIAA1199 has the ability to degrade HA.

Inflammatory cytokines such as tumor necrosis factor-α (TNF-α), interleukin (IL)-1β, and IL-6 are typically elevated in RA patients^13,14^; however, the interactive network between cytokines and synoviocytes underlying RA pathogenesis is far from clear. FLS are the main source of n-HA in synovial fluid and homeostasis of HA metabolism in FLS is crucial for maintaining normal synovial function. In arthritic conditions, the stimulation of inflammatory factors leads to HA metabolic imbalance in FLS, which in turn changes the size and bioactivity of HA. Previous studies have shown that the expression of KIAA1199 in skin fibroblasts can be inversely regulated by histamine and transforming growth factor-β1 (TGF-β1)^7,15,16^. However, the regulatory mechanism of KIAA1199 expression in RA FLS remains to be investigated.

In the present study, a positive correlation between KIAA1199 and LMW-HA in clinical serum and synovial samples was obtained for the first time. Animal experiments revealed that neutralizing anti-KIAA1199 monoclonal antibody (mAb) had a therapeutic effect on collagen-induced arthritis (CIA) mice. On this basis, we innovatively identified a new pathway for sKIAA1199-mediated HA degradation and further elucidated its mechanism. Our results confirmed the contribution of sKIAA1199 to RA progression and provided a new strategy for RA treatment by blocking sKIAA1199-mediated HA degradation.

## Results

### sKIAA1199 is positively correlated with LMW-HA in serum and synovial fluid

**Fig. 1:**
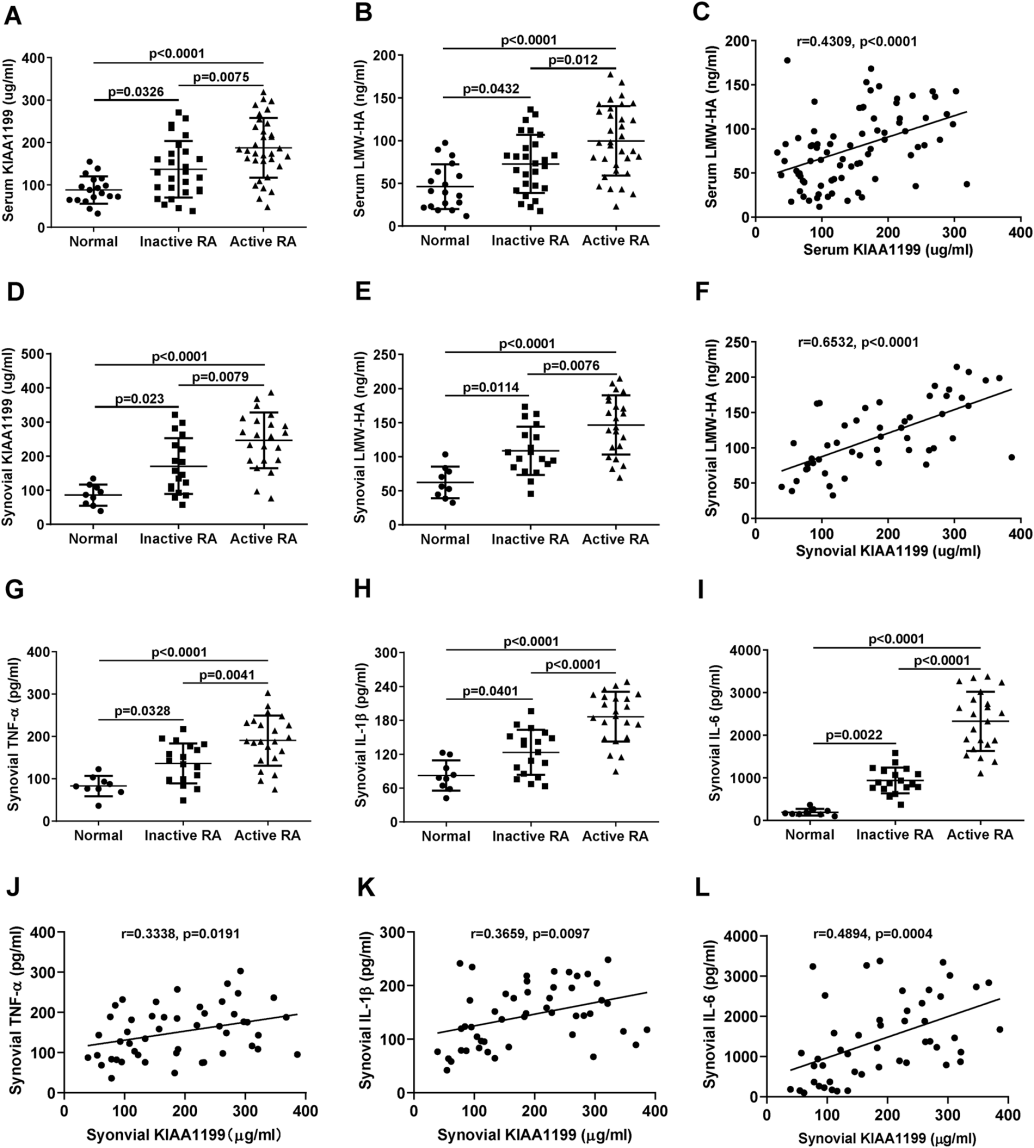
The levels of KIAA1199, LMW-HA, and inflammatory cytokines in serum and synovial fluid. **A**, **B** Serum levels of KIAA1199 (**A**) and LMW-HA (MW < 100 kDa) (**B**) in normal subjects (*n* = 18), inactive RA (*n* = 27), and active RA (*n* = 33) patients were determined by ELISA. **C** Correlation between serum KIAA1199 and LMW-HA was obtained by the Pearson’s coefficient analysis (*r* = 0.4309, *p* < 0.0001, *n* = 78). **D**, **E** Synovial levels of KIAA1199 (**D**) and LMW-HA (**E**) in normal subjects (*n* = 9), inactive RA (*n* = 18), and active RA (*n* = 22) patients. **F** Linear correlation between KIAA1199 and LMW-HA in the synovial fluid (*r* = 0.6532, *p* < 0.0001, *n* = 49). **G**–**I** TNF-α (**G**), IL-1β (**H**), and IL-6 (**I**) levels in the synovial fluid of normal subjects (*n* = 9), inactive (*n* = 18), and active (*n* = 22) RA patients. **J**, **K** Correlation analysis of synovial TNF-ɑ (**J**), IL-1β (**K**), IL-6 (**L**), and KIAA1199, respectively (*n* = 49). All of the reactions were conducted in triplicate. Data were presented with mean ± SD.

The serum levels of KIAA1199 and LMW-HA (MW < 100 kDa) in RA patients were significantly higher than those in normal subjects (Fig. 1A, B), between which a positive correlation was found in Fig. 1C (*r* = 0.4309, *p* < 0.0001). Similar results with better correlation coefficients were also observed in synovial fluid (Fig. 1D–F, *r* = 0.6532, *p* < 0.0001). Meanwhile, inflammatory factors TNF-α, IL-1β, and IL-6 were collectively increased in synovial fluid of RA patients, especially those with active RA (Fig. 1G–I). Among these cytokines, IL-6 showed the best correlation with sKIAA1199 in synovial fluid (Fig. 1J–L). In addition, we also detected the levels of HYAL1 and HYAL2 (two important HYALs) in synovial samples. The results showed that HYAL2 (a cell surface enzyme) level in RA patients was slightly higher than that in normal subjects, yet no significant difference was found (Supplementary Fig. S1). By contrast, HYAL1 (a lysosomal enzyme) was almost undetectable in synovial fluid of both normal subjects and RA patients (data not shown).

### Neutralizing anti-KIAA1199 mAb effectively reduces the severity of arthritis in CIA mice

**Fig. 2:**
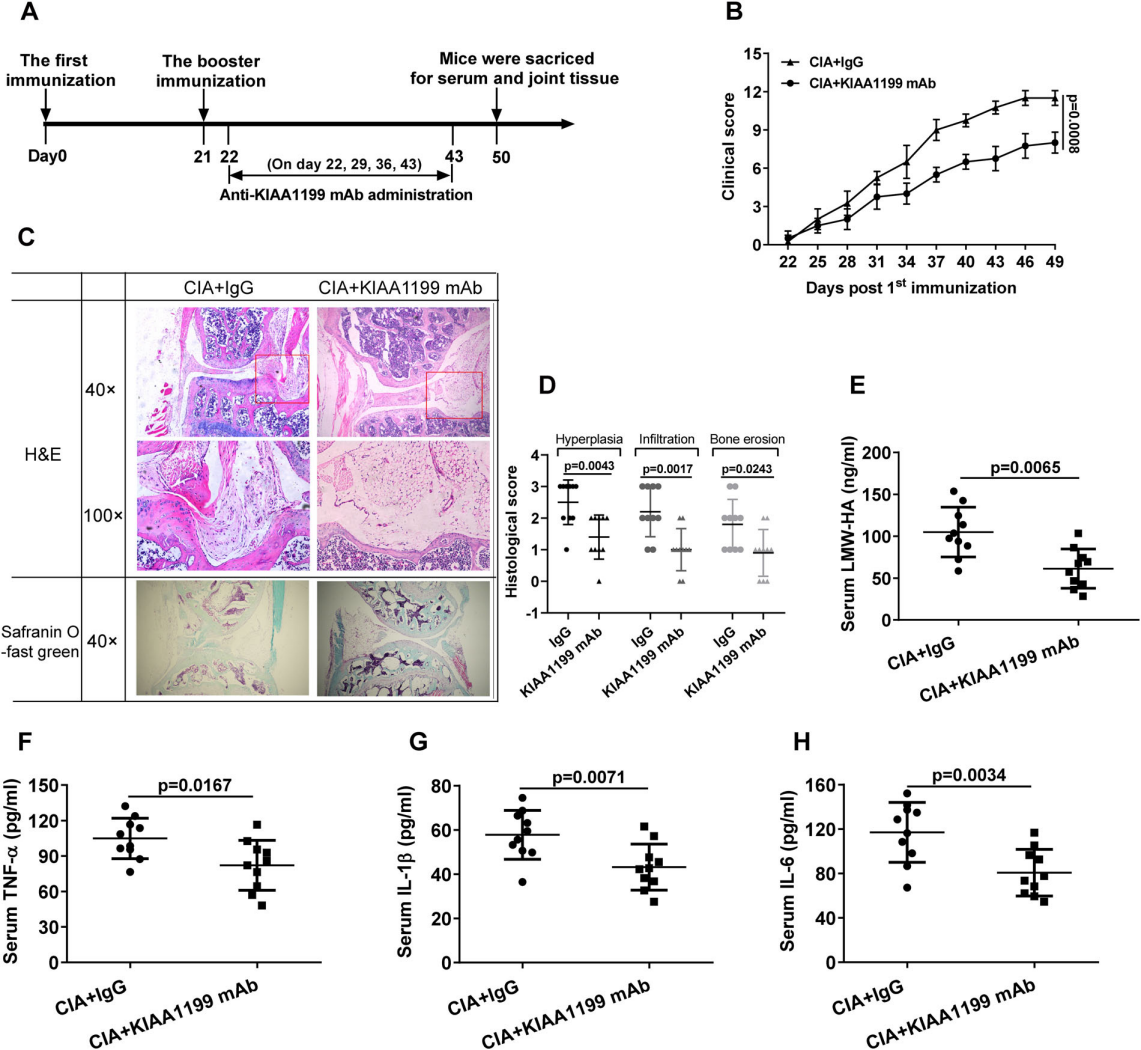
Therapeutic effect of neutralizing anti-KIAA1199 mAb on CIA mice. **A** Timeline of neutralizing anti-KIAA1199 mAb and IgG (Control) treatment in CIA mice (*n* = 10 per group). **B** Clinical scores of CIA mice treated with KIAA1199 mAb and IgG. The severity of arthritis in mice was inspected by two independent observers every 3 days in a double-blind manner and graded on a scale of 0–4 (0 = no erythema and swelling, 1 = erythema and mild swelling on tarsals or ankle joints, 2 = erythema and mild swelling from tarsals to ankles, 3 = erythema and moderate swelling from ankles to metatarsal joints, 4 = erythema and severe swelling around the ankles, feet, and digits or ankylosis of the limbs). Each paw was graded, so the maximum possible clincial score for each mouse was 16. **C**, **D** Histopathological analysis of CIA mice. Knee joints of the mice were stained by H&E and safranin O-fast green (**C**). Histological scores for synovial hyperplasia, inflammatory cell infiltration, and cartilage and bone erosion were shown as in **D**. **E**–**H** Serum levels of LMW-HA (**E**), IL-6 (**F**), IL-1β (**G**), and TNF-α (**H**) in CIA mice treated with antibodies for 4 weeks were determined by ELISA. All reactions were conducted in triplicate and data were presented with mean ± SD.

To evaluate the role of sKIAA1199 in RA pathogenesis, neutralizing anti-KIAA1199 mAb was prepared to intervene CIA mice as illustrated in Fig. 2A. Compared with IgG-treated controls, less paw swelling and toe joint damage were observed in CIA mice treated with KIAA1199 antibody for 4 weeks (Supplementary Fig. S2). Accordingly, the clinical and histological scores of CIA mice treated with KIAA1199 antibody were significantly lower than those of control mice (Fig. 2B–D). In addition, the serum levels of LMW-HA and inflammatory factors TNF-α, IL-1β, IL-6 in the CIA mice were distinctly reduced after antibody treatment (Fig. 2E–H). The therapeutic effect of neutralizing KIAA1199 mAb on CIA mice confirmed the role of sKIAA1199 in the pathogenesis of RA.

### sKIAA1199-mediated HA degradation requires RA FLS cell membranes

**Fig. 3:**
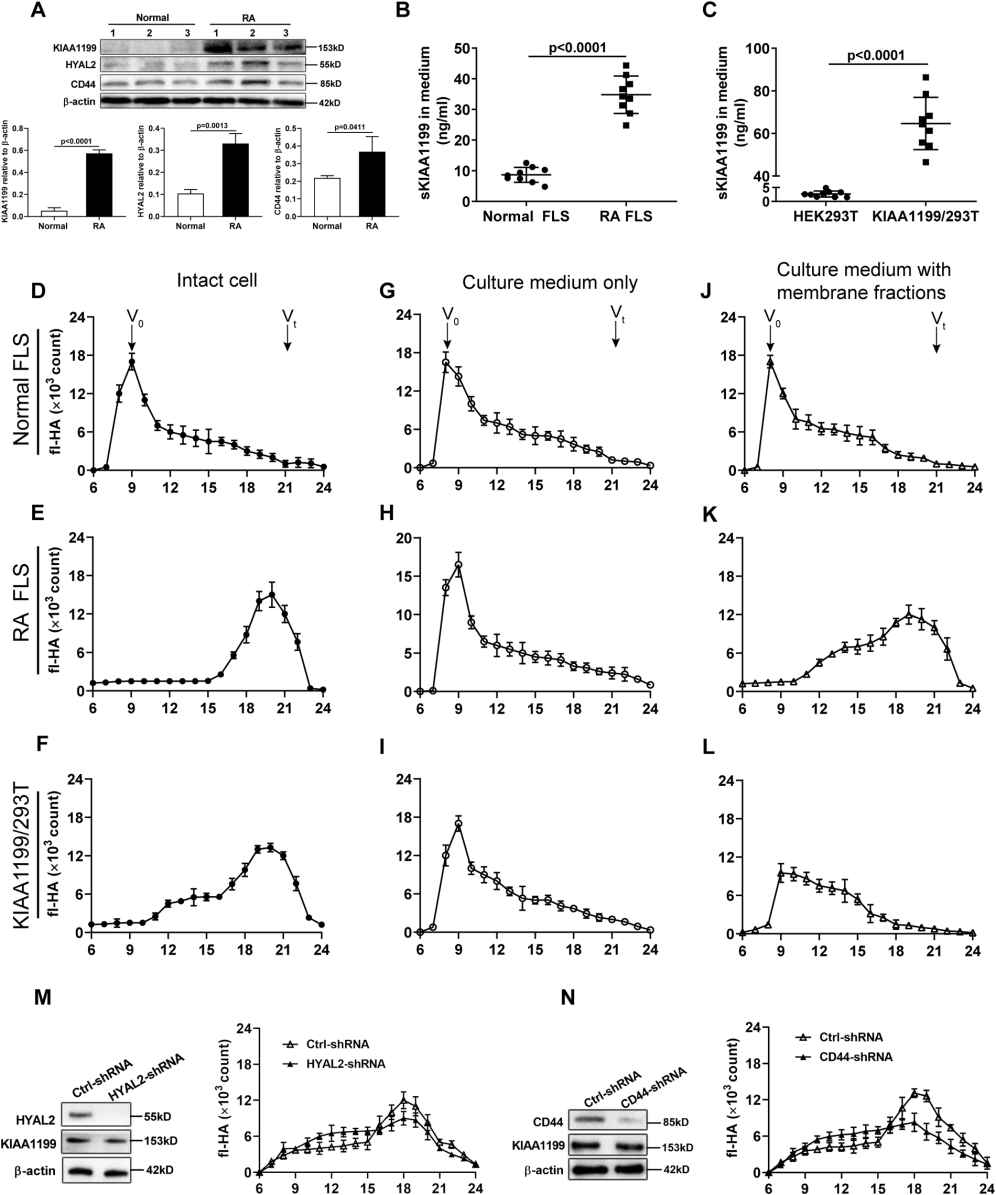
Degradation of exogenous fl-HA by intact cells and their media without or with the addition of cell membrane fractions. **A** Western blot analysis of KIAA1199, HYAL2, and CD44 protein in FLS from normal subjects (*n* = 3) and RA patients (*n* = 3). The relative expression of protein was calculated by the gray ratio of protein band to β-actin. **B**, **C** Comparison of sKIAA1199 in the culture media of normal FLS and RA FLS (**B**), HEK293T and KIAA1199/293T cells (**C**). Normal FLS and RA FLS were isolated from synovial tissues of normal subjects (*n* = 3) and active RA patients (*n* = 3), respectively. **D**–**L** The HA-degrading activity of intact normal FLS, RA FLS, and KIAA1199/293T cells (**D**–**F**), their culture media without (**G**–**I**) or with cell membrane fractions (**J**–**L**). Arrow head indicated the positions of the void volume (*V*_o_) and the total volume (*V*_t_) of the column. **M**, **N** The effect of *HYAL2* knockdown (**M**) or *CD44* knockdown (**N**) in RA FLS on the HA-degrading activity of membrane-bound sKIAA1199. All experiments were performed at least in triplicates, the data were presented as mean ± SD.

The therapeutic effect of KIAA1199 mAb on RA in combination with the catalytic mode of cell surface HYAL led us to speculate that sKIAA1199 may be involved in HA catabolism through adhesion to cell membranes. We first verified that the mRNA expression of KIAA1199, HYAL2, and CD44 in RA synovial tissues was notably increased, especially in patients with active RA (Supplementary Fig. S3). It was further found that higher levels of these three proteins in RA FLS compared with normal FLS (Fig. 3A) and more KIAA1199 was detected in the medium from RA FLS (Fig. 3B). In parallel, HEK293T cells transfected with vectors containing full-length KIAA1199 cDNA (named KIAA1199/293T) also highly secreted KIAA1199 into the medium (Fig. 3C). Subsequently, the HA-degrading ability of intact cells (FLS, RA FLS, and KIAA1199/293T) and their culture media without or with their own cell membrane fractions were determined, respectively. The results indicated that KIAA1199/293T and RA FLS showed similar high-efficiency profiles for fluorescein-labeled HA (fl-HA) degradation, whereas the HA-degrading ability of normal FLS with low expression of KIAA1199 was extremely low (Fig. 3D–F). However, almost all exogenous fl-HA remained intact when incubated in sKIAA1199-rich media either from RA FLS or from KIAA1199/293T cells, as in the normal FLS medium (Fig. 3G–I). Surprisingly, fl-HA could be efficiently degraded in sKIAA1199-rich medium supplemented with RA FLS membrane fractions, but not in the same medium with KIAA1199/293T cell membranes (Fig. 3J–L). To eliminate the possible effect of membrane-anchored HYAL2 on HA degradation, the expression of *HYAL2* and *CD44* genes in RA FLS were silenced, respectively. Then, the HA-degrading assays showed that knockdown of HYAL2 and CD44 did not alter the trend of HA degradation mediated by membrane-bound sKIAA1199 (Fig. 3M, N), suggesting that some membrane molecule(s) might be required for this process.

### Adhesion of sKIAA1199 to cell membrane by binding to ANXA1

**Fig. 4:**
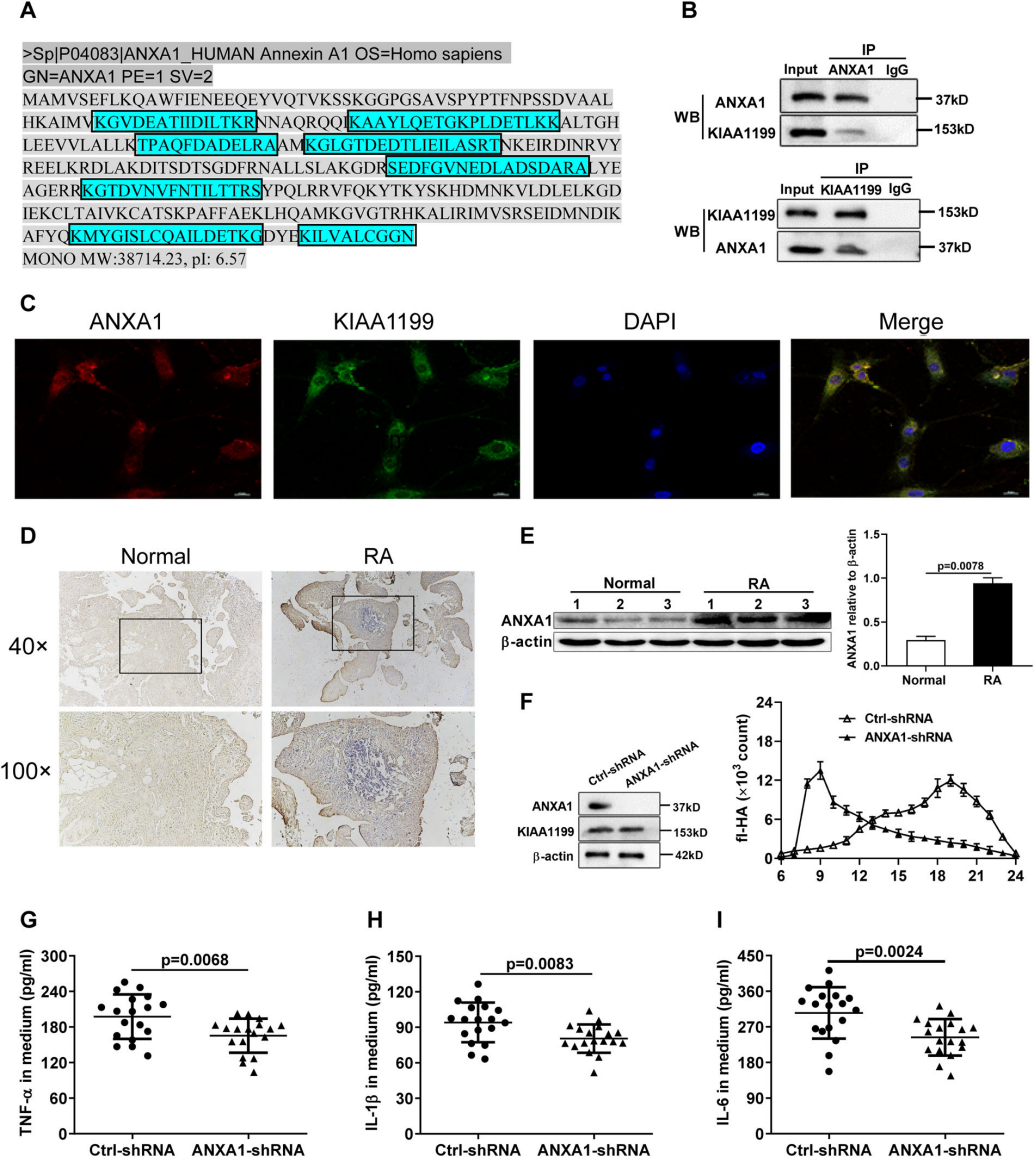
Identification of ANXA1 as a KIAA1199-binding membrane protein required for its HA-degrading activity. **A** Amino acid sequences of ANXA1 identified by LC-MS/MS (in blue rectangle). **B** The interaction of KIAA1199 with ANXA1 was analyzed by Co-IP using ANXA1 and KIAA1199 antibodies with the whole-cell lysates of RA FLS. **C** Colocalization of ANXA1 and KIAA1199 on RA FLS cell membrane by immunofluorescent staining. Images were captured with a confocal microscopy at a magnification of ×400. **D** Immunohistochemical staining of ANXA1 was performed on the sections of joint tissues from RA patients and normal subjects with the magnification of ×40 and ×100. **E** Western blot analysis of ANXA1 protein in FLS from normal subjects (*n* = 3) and RA patients (*n* = 3). **F** The effect of ANXA1 knockdown on fl-HA degradation mediated by membrane-bound sKIAA1199. **G**–**I** The effect of AXNA1 knockdown on the secretion of TNF-α (**G**), IL-1β (**H**), and IL-6 (**I**) by FLS from RA patients (*n* = 6). All reactions were conducted in triplicate and data were presented with mean ± SD.

To obtain the membrane molecule(s) involved in sKIAA1199-mediated HA degradation, immunoprecipitation (IP) was performed using anti-KIAA1199 mAb with membrane protein extracts of RA FLS. The precipitated protein mixture was separated by SDS-polyacrylamide gel electrophoresis (PAGE) and then subjected to liquid chromatography-tandem mass spectrometry (LC-MS/MS) for identification (Supplementary Fig. S4). All identified proteins were listed in Supplementary Materials, among which ANXA1 was selected for verification due to its high amino acid coverage (Fig. 4A and Supplementary Materials: Identified proteins by LC-MS/MS).

ANXA1 was first identified as a KIAA1199-interacting membrane protein by Co-IP (Fig. 4B), which could be colocalized with KIAA1199 on RA FLS cell membrane by immunofluorescence staining (Fig. 4C). High expression of ANXA1 was found in synovial tissues and FLS from RA patients (Fig. 4D, E). More importantly, silencing the expression of ANXA1 in RA FLS strongly inhibited the HA-degrading activity of membrane-bound sKIAA1199 (Fig. 4F) and resulted in a collective reduction of TNF-α, IL-1β, and IL-6 levels in the medium (Fig. 4G–I).

### The G8 domain is essential for KIAA1199 binding to ANXA1

**Fig. 5:**
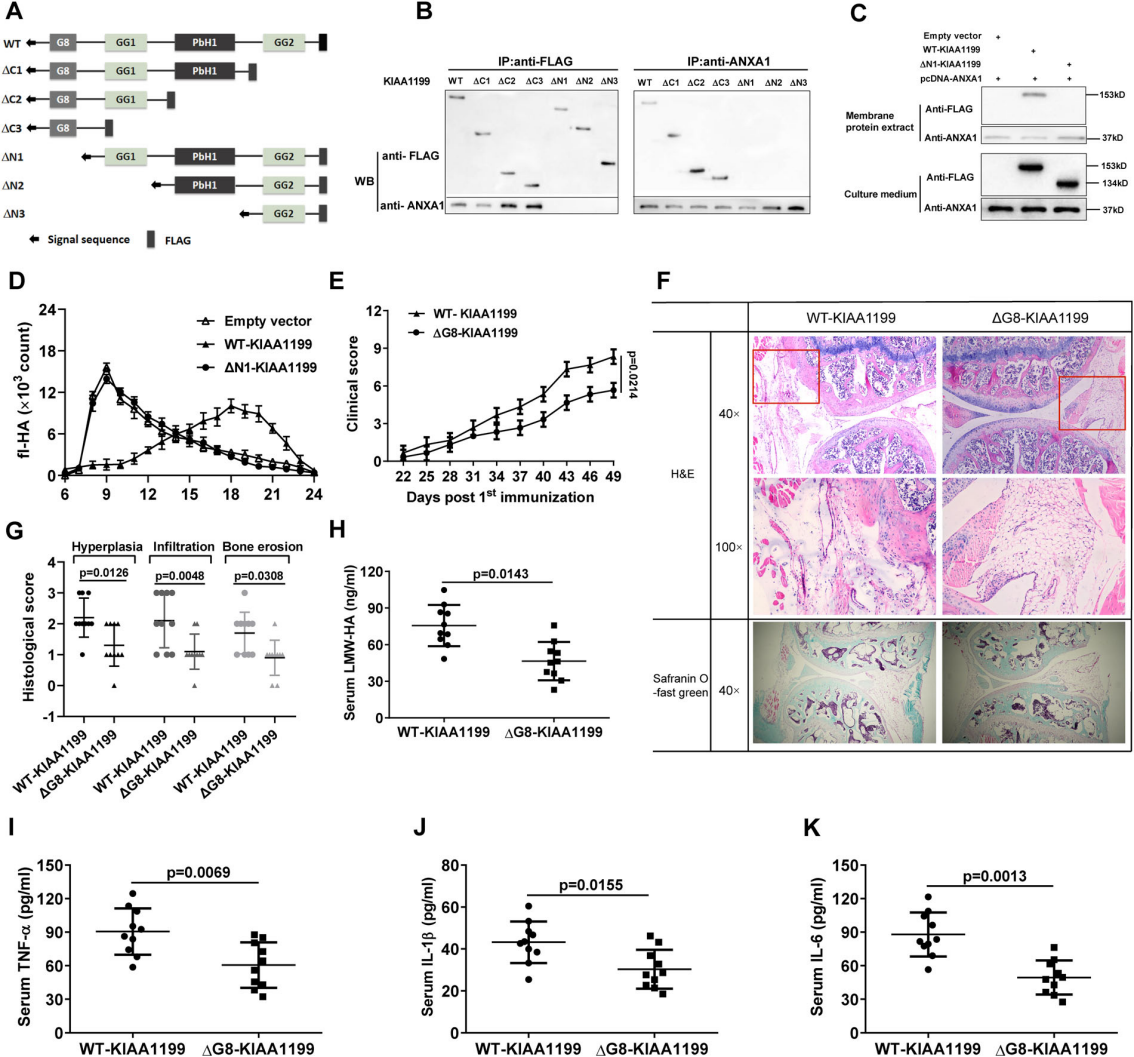
G8 domain is necessary for sKIAA1199 binding to membrane ANXA1 and its in vivo function. **A** Schematic diagram of KIAA1199 domain mapping. Wild-type (WT) KIAA1199 and various mutants have a N-terminal signal peptide sequence and a C-terminal FLAG tag. **B** The interaction of ANXA1 with WT- or various domain-deleted KIAA1199 mutants was analyzed by Co-IP assays using anti-FLAG and anti-ANXA1 antibodies. WT-KIAA1199 is a 153 kDa protein and the molecular weights of ΔC1, ΔC2, ΔC3, ΔN1, ΔN2, and ΔN3 mutants are predicted to be 95, 33, 19, 134, 105, and 40 kDa, respectively. **C** Detection of WT-KIAA1199 and ΔN1-KIAA1199 mutant (without G8 domain) in membrane protein extracts and in the medium of ANXA1/293T cells transfected with vectors containing WT-KIAA1199 or ΔN1-KIAA1199 cDNAs, respectively. Empty vector was used as a negative control. **D** The effect of secreted WT-KIAA1199 and ΔN1-KIAA1199 on HA degradation in the media mixed with the membrane fractions of ANXA1/293T cells. **E** Clinical scores of CIA mice with *kiaa1199-KO* background following injection of adenovirus-coated WT-KIAA1199 and ΔG8-KIAA1199 vectors for 4 weeks, respectively. **F**, **G** Knee joints of CIA mice were stained by H&E and safranin O-fast green (**H**). Histological scores for synovial hyperplasia, inflammatory cell infiltration, and cartilage and bone erosion were shown in **I**. **H**–**K** Serum levels of LMW-HA (H) and cytokines TNF-α (**I**), IL-1β (**J**), and IL-6 (**K**) in CIA mice after 4 weeks of treatment. All experiments were performed at least in triplicates, the data are presented as mean ± SD.

As shown in Fig. 5A, domain mapping was designed to investigate which domain of KIAA1199 directly mediated its binding to ANXA1. Specifically, various vectors carrying full-length (wild type, WT) KIAA1199 and several domain-deleted mutants were constructed to transfect ANXA1/293 T cells, respectively. Co-IP results showed that only WT-KIAA1199 and G8-containing mutants (ΔC1, ΔC2, and ΔC3) could co-precipitate with ANXA1 (Fig. 5B). Then we selectively extracted the membrane proteins from ANXA1/293T cells transfected with WT- and ∆N1-KIAA1199 vectors. Subsequently, the membrane-bound ability and HA-degrading activity of WT-KIAA1199 and ∆N1-KIAA1199 were compared. As expected, although highly secreted into culture medium, ∆N1-KIAA1199 mutant without G8 domain could not adhere to cell membranes as effectively as WT-KIAA1199 (Fig. 5C). As a result, exogenous fl-HA was no longer degraded by ΔN1-KIAA1199 mutant even when ANXA1-rich membrane was added to the culture medium (Fig. 5D).

To further verify the effect of G8 domain on KIAA1199 function in vivo, we established CIA models using global KIAA1199-deficient (*Kiaa1199*-KO) mice for rescue experiments, as shown in Supplementary Fig. S5. As predicted, deletion of *kiaa1199* gene did increase the mice’s resistance to collage-induced arthritis (Supplementary Fig. S6). The following rescue experiments showed that intra-articular injection of adenovirus-mediated WT-KIAA1199 vectors was more effective in impairing this resistance than ∆G8-KIAA1199 mutant, mainly manifested by higher arthritis scores, more severe inflammatory cell infiltration, and cartilage erosion (Fig. 5E–G). In addition, the serum levels of LMW-HA and TNF-α, IL-1β, and IL-6 were consistently elevated in the mice injected with WT-KIAA1199 vectors (Fig. 5H–K).

### IL-6 promotes KIAA1199 expression via the PI3K/Akt/NF-κB signaling pathway in RA FLS

**Fig. 6:**
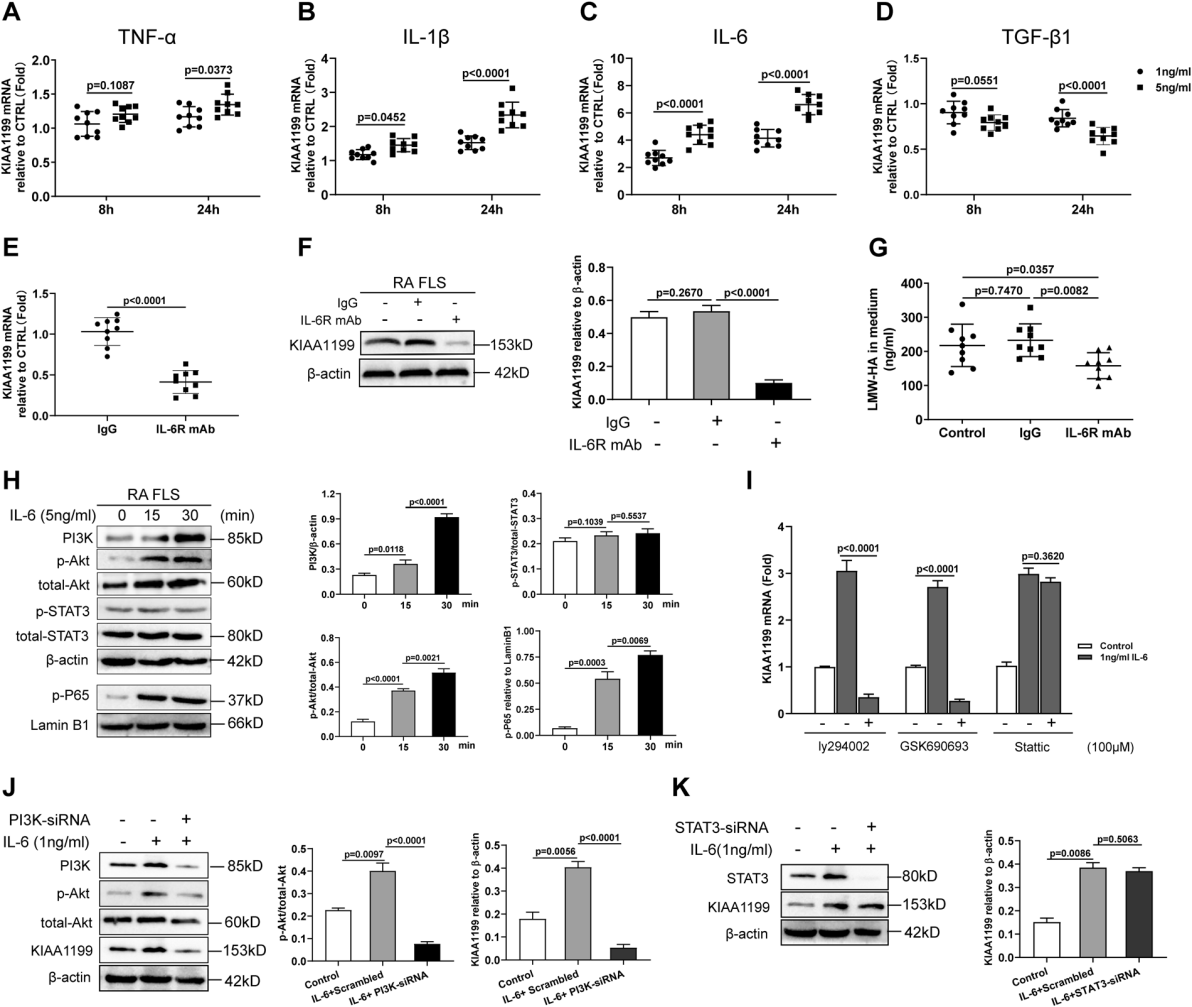
IL-6 promotes KIAA1199 expression through the PI3K/Akt/NF-κB signaling pathway in RA FLS. **A**–**D** Relative mRNA expression of KIAA1199 in FLS from healthy subjects (*n* = 9) stimulated without (Control) or with 1 and 5 ng/ml TNF-α (**A**), IL-1β (**B**), IL-6 (**C**), and TGF-β1 (**D**), respectively. **E**–**G** Inhibitory effect of IL-6R blocking mAb on the expression of KIAA1199 mRNA (**E**), protein (**F**) in FLS from RA patients (*n* = 9), and the formation of LMW-HA in the medium (**G**). **H** Western blot analysis of cytoplasmic PI3K, p-Akt, toatl-Akt, p-STAT3, total-STAT3, and intranuclear p-P65 in RA FLS stimulated with 5 ng/ml IL-6 at 0, 15, 30 min. **I** Effect of ly294002, GSK690693, and Stattic on the mRNA expression of KIAA1199 in response to 1 ng/ml IL-6. **J**, **K** Effect of siRNA knockdown of PI3K (**J**) and STAT3 (**K**) on the protein expression of KIAA1199 in RA FLS stimulated without or with 1 ng/ml IL-6. All reactions were conducted in triplicate and the data were presented with mean ± SD.

To explore the effect of inflammatory factors on KIAA1199, quantitative PCR was used to detect the mRNA expression of KIAA1199 in FLS stimulated with TNF-ɑ, IL-1β, IL-6, and TGF-β1, respectively. The results showed that IL-6 was the most effective stimulus to induce KIAA1199, whereas TGF-β1 slightly downregulated its expression (Fig. 6A–D). Moreover, elevated IL-6 levels were found in RA FLS medium cultured for 10 days compared with normal FLS (Supplementary Fig. S7), suggesting a promotion effect of autocrine IL-6 on KIAA1199 expression in RA FLS. In response, blocking IL-6 receptor (IL-6R) with specific antibody not only inhibited the expression of KIAA1199 in RA FLS (Fig. 6E, F), but also reduced the formation of LMW-HA in the medium (Fig. 6G). To further elucidate the mechanism by which IL-6 induced KIAA1199, we examined the protein changes in the IL-6R signaling pathways and found that phosphatidylinositol 3-kinase (PI3K), p-Akt, and p-P65 levels in IL-6-stimulated RA FLS were significantly elevated (Fig. 6H). Echoing the above findings, LY294002 (PI3K inhibitor) and GSK690693 (Akt inhibitor) greatly inhibited the mRNA expression of KIAA1199, whereas Stattic (signal transducer and activator of transcription 3 (STAT3) inhibitor) had no such effect (Fig. 6I). Furthermore, specific small interfering RNA (siRNA) sequences were synthesized to knock down the expression of PI3K and STAT3 in RA FLS, respectively. The results showed that PI3K knockdown greatly inhibited Akt phosphorylation and KIAA production in IL-6-stimulated RA FLS (Fig. 6J). In contrast, the induction of KIAA1199 by IL-6 was not significantly affected by STAT3 knockdown (Fig. 6K). Therefore, we proposed that highly expressed KIAA1199 in RA FLS might be induced by IL-6-mediated PI3K/AKT/nuclear factor-κB (NF-κB) pathway.

## Discussion

In inflamed joints, HA metabolic disorder is suggested to be related to the pathogenesis of RA, although the mechanism is not fully understood. Recently, a novel HA degradation pathway mediated by KIAA1199 was elucidated in arthritic synovium^7^, indicating a link between KIAA1199 and RA pathogenesis. Of note, sKIAA1199 has been identified as a diagnostic biomarker for several diseases including RA^9,17^, although its function remains ambiguous. Here we demonstrated for the first time that KIAA1199 and LMW-HA levels were positively correlated in clinical serum and synovial fluids. The following in vivo study showed that the blockade of sKIAA1199 was effective in alleviating arthritis development in CIA mice, as evidenced by less paw swelling and joint destruction, reduced inflammatory cell infiltration, lower serum levels of cytokines, and LMW-HA. All of these findings lead us to speculate that sKIAA1199 may be associated with HA catabolism, but whether and how it participates in this process remains to be experimentally clarified.

At present, the widely accepted HA-degrading pattern in normal tissues is as follows: cell surface HYAL2 cleaves extracellular n-HA into medium-sized HA, which are then internalized and further degraded into LMW-HA fragments by lysosomal HYAL1^18^. HYAL1 and HYAL2 are considered to be the most important members of HYAL family in various tissues, and increased HYAL1 and/or HYAL2 has been reported in rheumatoid synovium^19^. Although their presence in synovial fluid remains controversial, available data suggest that HYAL1 is more likely to be a lysosomal enzyme, whereas HYAL2 functions primarily on cell surface^20,21^. Our results revealed no significant difference in HYAL1 and HYAL2 levels in synovial fluid between RA patients and normal subjects. In fact, both enzymes rarely function in synovial fluid due to their preference for acidic pH^20,22^. In addition, no other functional HYALs has been identified in synovial fluid so far.

Consistent with previous studies^7,10^, KIAA1199/293T with high expression of KIAA1199 acquired the ability to degrade exogenous HMW-HA and RA FLS. However, the exogenous HA could not be degraded in sKIAA1199-rich medium either from RA FLS or KIAA1199/293T, confirming that sKIAA1199 alone did not have HA-degrading activity. Despite the lack of transmembrane domain, KIAA1199 is highly similar to the extracellular domains of TMEM2, a newly identified cell surface HYAL^22^. Hence, we hypothesized that sKIAA1199 may be engaged in extracellular HA degradation by adhesion to cell membranes. Fortunately, exogenous HA could indeed be degraded effectively when incubated in KIAA1199-rich medium supplemented with cell membrane fractions of RA FLS, which supported our hypothesis. Interestingly, the HA-degrading activity of sKIAA1199 was almost lost when the membrane fractions of KIAA1199/293T cells rather than RA FLS were added into the medium, indicating certain molecule(s) on RA FLS cell membrane may be required for sKIAA1199 involving in HA degradation. In addition, KIAA1199 was suspected to regulate HYAL2-mediated HA degradation, although not proven^23^. Here we showed that the expression of HYAL2 and CD44 in RA FLS increased consistently with that of KIAA1199, but sKIAA1199-mediated HA degradation was not dependent on membrane HYAL2 and CD44. It is reasonable to surmise that HA degradation is a dynamic, synergistic process involving multiple enzymes and pathways. The association of KIAA1199 with other molecules in this process still needs further study.

We identified ANXA1 as a KIAA1199-binding protein for the first time, by which sKIAA1199 could be attached to cell membrane to degrade extracellular HA. In concert with previous studies ^24,25^, we confirmed that ANXA1 was notably increased in synovial tissues and FLS of RA. As a large protein with one G8 domain, two GG domains and four PbH1 domains, KIAA1199 has a structural basis for interactions with other molecules. Several studies have elucidated the biological function of KIAA1199 in the epidermal growth factor receptor (EGFR), fibroblast growth factor receptor (FGFR), and Wnt/β-catenin signaling pathways^23,26,27^. Although initially defined as a negative regulator of inflammation^28^, ANXA1 was reported to induce MMP-1 secretion in RA FLS in response to TNF-α stimulation^29^, showing its pro-inflammatory effect. The dual effects of ANXA1 on inflammation has also been observed in animal models. For instance, ANXA^−/−^ mice were more susceptible to induction of arthritis^30^. In contrast, injection with exogenous ANXA1 exacerbated the severity of arthritis in CIA mice^31^. The role of ANXA1 in HA metabolism needs to be further verified in animal model in the future.

As none of the G8-deleted KIAA1199 mutants co-precipitated with ANXA1 antibody, the G8 domain of KIAA1199 was essential for its binding to ANXA1. As expected, deletion of G8 domain (∆N1-KIAA1199) deprived KIAA1199 of the HA-degrading activity, further verifying that attachment to cell membrane was necessary for sKIAA1199-mediated HA degradation. G8 domain was predicted to mediate extracellular ligand binding^32^. Intact G8 domain has been reported to be essential for KIAA1199 binding to its intracellular receptor Plexin A2^33^. To further verify the effect of G8 domain on KIAA1199 function in vivo, global *kiaa1199*-KO mice were generated to induce CIA model. KIAA1199 deficiency significantly increased the mice’s resistance to arthritis induction. However, the following rescue experiments showed that intra-articular injection of WT-KIAA1199 was more effective in weakening the resistance than G8-deleted KIAA1199 mutant, which highlighted the importance of G8 domain for KIAA1199 in RA pathogenesis. Of course, our results did not exclude the possibility of sKIAA1199 interacting with other extracellular or membrane molecules or even other unidentified HYALs in synovial fluid. In an earlier study, an endo-*N*-acetylglucosaminidase was reported in the medium of human skin fibroblasts^34^, which could cleave HMW-HA into ~40 kDa small fragments in a catalytic mode similar to intracellular KIAA1199^7^.

The levels of inflammatory TNF-α, IL-lβ, and IL-6 are typically elevated in serum and synovial fluid of RA patients, especially those with active RA. Broad effects of cytokines on joint tissues including synovial hyperplasia, angiogenesis, bone and cartilage destruction have been widely reported^13,35^. TNF-α and IL-1β were reported to promote HA degradation by increasing the synthesis of HYALs in RA synovial tissues^36^. Blocking TNF-α and IL-6 with antibodies has been licensed for the treatment of patients with severe RA. As a new favorite mediating HA degradation, KIAA1199 expression in skin fibroblasts could be reversibly regulated by histamine and TGF-β1^36^. However, in this study, IL-6 was shown to be a key driver of KIAA1199 expression in RA FLS, as observed in colon fibroblasts^12^. As a downstream mediator of TNF-α and IL-lβ, serum IL-6 is positively associated with the RA disease activity index^13,37^. Therefore, it is possible that abundant IL-6 in synovial fluid can sufficiently interact with adjacent synovial cells to further amplify the pro-inflammatory effects of TNF-α and IL-lβ. In an animal study, mice with IL-6 deletion showed milder severity of arthritis, less cartilage damage, and more antibody in the development of antigen-induced arthritis^38^. Our results also confirmed that the blockade of IL-6R could suppress KIAA1199 expression and LMW-HA formation. In addition to the classic JAK/STAT3 pathway, IL-6 also contributes to its pathological functions in many inflammatory diseases by mediating trans-signaling activation of the PI3K/Akt pathway^39^. Here we found that IL-6 significantly increased the levels of PI3K, p-Akt, and p-P65 in RA FLS. Moreover, specific inhibitor or knockdown of PI3K and Akt rather than STAT3 greatly inhibited the mRNA expression of KIAA1199. Promoter analysis have revealed four NF-κB binding sites upstream of the TATA box in *kiaa1199* gene promoter and the binding of P65 to the fourth site effectively activated its transcription^40^. Conversely, P65 deletion almost eliminated KIAA1199 expression in several tumor cell lines^33^. Taken together, we proposed that the PI3K/Akt/NF-κB axis activated by IL-6 was the main pathway promoting high expression of KIAA1199 in RA FLS. In addition, TGF-β1 was reported to relieve chronic inflammatory diseases including RA^41,42^ and slightly downregulate KIAA1199 expression via the PI3K/Akt signaling pathway^16^. Thus, a complex, synergistic mechanism involving increased IL-6 and decreased TGF-β1 may play a crucial role in promoting KIAA1199 expression and accelerating HA degradation in RA FLS.

Taken together, this study elucidated the role and mechanism of sKIAA1199 in extracellular HA degradation and provided in vivo evidence for sKIAA1199’s contribution to RA progression. Inhibition of sKIAA1199-mediated HA degradation to maintain the homeostasis of HA metabolism in joints is suggested as a therapeutic strategy for RA disease. More research will be required to resolve the structural alternation of sKIAA1199 and explore other possible functions in arthritic conditions.

## Materials and methods

### Patients and samples

Clinical samples were collected from the First Affiliated Hospital of Wenzhou Medical University from June 2016 to May 2018. Specifically, serum was obtained from 60 RA patients and 18 normal subjects, and knee synovial fluid and synovium from 40 RA patients who underwent arthrocentesis or joint replacement surgeries and 9 normal subjects with high amputation. All patients fulfilled the 2010 ACR criteria, which were categorized as inactive or active RA according to the disease activity score in 28 joints (DAS28) (inactive RA: DAS28 < 3.2; active RA: DAS28 ≥ 3.2). Each participant submitted a signed consent form approved by the Clinical Research Ethics Committee of the First Affiliated Hospital of Wenzhou Medical University. Clinical data for all participants were available from Supplementary Tables S1 and S2.

### ELISA assay

Commercial enzyme-linked immunosorbent assay (ELISA) kits were used to determine the levels of LMW-HA (#DHYAL0, R&D Systems), HYAL1 (#DY7358, R&D Systems), HYAL2 (#E1126h, EIAab), TNF-α (#MTA00B for human; #DAT00D for mouse, R&D Systems), IL-1β (#DY201 for human; #MLB00C for mouse, R&D Systems), and IL-6 (#D6050 for human; #M6000B for mouse, R&D Systems) in serum, synovial fluid, and culture medium according to the manufacturer’s instructions. The samples for LMW-HA determination were properly diluted, and centrifuged through Amicon Ultra-0.5 Centrifugal Filter with a 100 kDa cutoff (Millipore). sKIAA1199 was measured by sandwich ELISA method using KIAA1199 mAb (sc-293483, Santa Cruz). The absorbance of 450 nm was determined using Bio-Rad iMarkTM microplate reader (Bio-Rad).

### Construction, intervention, and clinical evaluation of CIA model

DBA/1 and C57BL/6 mice were purchased from Shanghai Lab Animal Research Center and were raised under pathogen-free conditions. *Kiaa1199*-KO mice were constructed with C57BL/6 mice using Cre/loxP system as described previously^43^. All animal experiments were approved by the Institutional Animal Care and Use Committee of Wenzhou Medical University.

CIA model was established using DBA/1 and *kiaa1199*-KO C57BL/6 mice, respectively. On day 0, each DBA/1 mouse was immunized by intradermal injection of 200 µg type II bovine collagen (#20021, Chondrex) emulsified in Freund’s complete adjuvant (#7009, Chondrex) at the base of tail and type II chicken collagen was used for the immunization of C57BL/6 mice. On day 21, the mice were given a booster immunization with 200 µg type II bovine or chicken collagen emulsified in Freund’s incomplete adjuvant (#7002, Chondrex). Neutralizing anti-KIAA1199 mAb was obtained by genetic immunization method developed previously^44,45^, 200 μg neutralizing anti-KIAA1199 mAb or purified mouse IgG (Sigma-Aldrich) were intraperitoneally injected into two groups of CIA mice (*n* = 10 per group) constructed with DBA/1 weekly, and a total of four injections from day 22 to day 43. In the rescue experiments, two groups of CIA mice (*n* = 10 per group) constructed with *Kiaa1199*-KO mice were treated weekly by intra-articular injection of adenovirus-coated vectors containing WT- KIAA1199 or ∆G8-KIAA1199 cDNAs (GeneChem, Shanghai) to each ankle joint with a dose of 10^7^ particles. On day 50, the mice were killed to collect serum and joint tissues for further study. The detailed immunization and intervention schedules were presented in Fig. 2A and Supplementary Fig. S5.

### Histological and immunohistochemical analysis

Knee joints of CIA mice were dissected and fixed in 4% paraformaldehyde for 24 h followed by decalcification in 50 nM EDTA (pH 7.2 ~ 7.3) for 4 ~ 5 weeks. The decalcified joints were embedded, sectioned, deparaffinized, rehydrated, and then stained with hematoxylin and eosin or safranin O-fast green. Histological scores were assessed as previously described^46^.

For immunohistochemical analysis of ANXA1, the sections of synovial tissues from RA patients and normal subjects were incubated with ANXA1 antibody (#AF3770, R&D Systems) followed by secondary antibody conjugated with horseradish peroxidase (HRP) and 3, 3′-diaminobenzidine (DAB) substrate for signal visualization. The images were captured using a Nikon photomicroscope.

### Cells, plasmids, overexpression, and RNA interference

Primary FLS was isolated from synovial tissues of normal and RA patients as previously described^5^, and was used between the third and fifth generations. Normal FLS, RA FLS, and HEK293T cell lines were maintained in Dulbecco’s modified Eagle’s medium supplemented with 10% fetal calf serum (Gibco) at 37 °C in 5% CO_2_.

pcDNA3.2-KIAA1199 containing a complete human KIAA1199 cDNA was kindly donated by Dr Marra (University of Zurich, Switzerland). pcDNA3.1-ANXA1 was constructed by inserting human ANXA1 cDNA into pcDNA3.1(-). HEK293T cells were transfected with pcDNA3.2-KIAA1199 and pcDNA3.1-ANXA1 mediated by Lipofectamine 2000 (#11668019, Invitrogen), and screened in 600 μg/ml neomycin (#IN0130, Solarbio). The cell lines with stable expression of KIAA1199 and ANXA1 were named KIAA1199/293T and ANXA1/293T, respectively. In addition, full-length KIAA1199 and various domain-deleted KIAA1199 cDNAs were amplified using specific primers (Supplementary Table S3), which were inserted separately into pCMV vector carrying a N-terminal signal peptide and a C-terminal FLAG tag. These newly constructed plasmids were transfected into ANXA1/293T and screened in 500 μg/ml hygromycin (#IH0160, Solarbio).

Lentivirus-enveloped short hairpin RNAs (shRNAs; Genechem, Shanghai) were used to silence the expression of *HYAL2*, *CD44*, and *ANXA1* in RA FLS according to the manufacturer’s instructions. GV102 vector was used as a negative control (Ctrl-shRNA). All shRNA-targeting gene sequences were listed in Supplementary Table S3. In addition, specific siRNA sequences (Genechem, Shanghai) were synthesized to knock down the expression of PI3K and STAT3 in RA FLS, respectively. Scrambled siRNAs were used as negative controls.

### Quantitative reverse-transcriptase PCR

Total RNA was extracted from cells or synovial tissues by Trizol reagent (Invitrogen) and the cDNAs were obtained by Primescript^TM^ RT reagent Kit with gDNA Eraser (Takara). Quantitative reverse-transcriptase PCR (qRT-PCR) was performed in CFX96 Touch Real-Time PCR Detection System (Bio-Rad) using TB Green™ Premix Ex Taq™ II kit (Takara). The qRT-PCR primers for *kiaa1199*, *hyal2*, *cd44*, and *anxa1* genes were described in Supplementary Table S3. The relative mRNA expression normalized to β-actin was calculated using the 2^−ΔΔCt^ method.

### Western blotting

Total protein, membrane protein, cytoplasmic protein, and nucleoprotein were extracted from RA FLS and HEK293T cells by ProteoPrep® Total Extraction Sample Kit (Sigma-Aldrich), ProteoPrep® membrane Extraction Kit (Sigma-Aldrich), and NE-PER™Nuclear and Cytoplasmic Extraction Reagents (Thermo Fisher Scientific), respectively. Protein in culture medium (100 ml) was pretreated by freeze-drying method and then dissolved in an appropriate amount of double distilled water. After quantification with Pierce BCA Protein Assay Kit (Thermo Fisher Scientific), 25 μg protein samples were separated by SDS-PAGE and then electrotransfered onto polyvinylidene difluoride membranes. After blocked in 5% skim milk for 2 h, the membranes were incubated with antibodies of KIAA1199 (#ab98947, Abcam), HYAL2 (#ab126099, Abcam), CD44 (#ab157107, Abcam), AXNA1 (#ab88865, Abcam), PI3K (#4249, CST), total-Akt (#2920, CST), p-Akt (Ser473) (#4060, CST), total-STAT3 (#4904, CST), p-STAT3 (Tyr705) (#9131, CST), p-P65 (Ser536) (#3036, CST), and then with matching HRP-conjugated secondary antibodies. Protein bands were visualized using ECL substrate (#35055, Thermo Fisher Scientific). β-Actin (#AP0060, Bioworld) and Lamin B1 (#12987-1-AP, Proteintech) were used as the internal reference of cytoplasmic protein and nucleoprotein, respectively. The intensity of protein bands were analyzed by Image J (v1.8.0).

### HA-degrading assay

The preparation of fl-HA and its degradation were performed in accordance with previous method with slight modification^20^. Briefly, normal FLS, RA FLS, and KIAA1199/293T cells were severally inoculated into 25 ml cell culture flasks until 95% confluence was reached. The cells and culture media were then collected, and 10 μg/ml fl-HA was incubated with intact cells (5 × 10^6^), culture media with or without cell membrane fractions for 3 days at 37 °C, 5% CO_2_, respectively. After centrifugation at 3000 r.p.m., the supernatant was analyzed by size-exclusion chromatography using Sepharose CL-2B column equilibrated with phosphate-buffered saline. Fractions of 2 ml were collected at 0.5 ml/min and the fluorescence was determined by a Varioskan Lux (Thermo Fisher Scientific).

### Immunoprecipitation

RA FLS membrane protein extracts were incubated overnight with anti-KIAA1199 mAb (#H00057214-M01, Abnova). Protein A/G Magnetic Agarose Beads (#78609, Pierce) were added and incubated for 4 h. After washing, the beads were boiled in 2 × SDS loading buffer for 5 min. The precipitated proteins were separated by SDS-PAGE and identified by Nano-LC-MS/MS on a Q Exactive mass spectrometer coupled to Easy nLC (Thermo Fisher Scientific).

To identify KIAA1199/ANXA1 interaction, RA FLS were collected and lysed in RIPA buffer with protease inhibitors and 1% NP-40. The lysates were incubated with anti-KIAA1199 mAb and anti-ANXA1 mAb (#ab214486, Abcam), respectively. For domain mapping, the lysates of HEK293T cells transfected with full-length and various domain-deleted KIAA1199 were incubated with anti-FLAG antibody (#MAB8529, R&D Systems) and anti-ANXA1 antibody, respectively. Protein A/G agarose beads were used to precipitate immune complexes, which were further identified by western blotting.

### Immunofluorescent staining

RA FLS slides were fixed, blocked, and incubated with KIAA1199 Rabbit antibody (#orb2292, Biorbyt) and ANXA1 Mouse antibody (#ab88865, Abcam), followed by secondary antibody of Anti-Rabbit IgG H&L (Alexa Fluor® 488) (#ab150073, Abcam) and Anti-Mouse IgG H&L (Alexa Fluor® 594) (#ab150108, Abcam). 4′,6-Diamidino-2-phenylindole (#D3571, Invitrogen) was used for nuclear staining. The staining images were obtained using a cofocal microscope.

### Cytokines and inhibitors

Normal FLS (5 × 10^4^) were plated into a six-well plate until 95% confluence was reached. The media were replaced with fresh ones containing 1 and 5 ng/ml recombinant human TNF-α (#300-01 A), IL-1β (#200-01B), IL-6 (#200-06), and TGF-β1 (#100-21) purchased from Peprotech, respectively. After cultured for 8 and 24 h, the cells were collected to extract total RNA for qRT-PCR analysis.

In the inhibitor-blocking experiments, RA FLS with 95% confluence in six-well plates were incubated for 1 h in the absence or presence of 100 μM/ml ly294002, GSK690693, and Stattic, respectively, followed by additional incubation for 8 h without (Control) or with 1 ng/ml IL-6. The total RNA was extracted from the collected cells for the qRT-PCR analysis of KIAA1199 mRNA.

### IL-6R blocking

RA FLS (5 × 10^4^) were seeded into each well of a six-well plate until they grew to 90% confluence, replaced the media with fresh ones containing 1 μg/ml HMW-HA (MW = 1100 kDa, Zztandard, Shanghai) together with 1 μg/ml IL-6R blocking mAb (BMS135, eBioscience), 1 μg/ml mouse IgG (Sigma-Aldrich), or medium alone (Control). Fresh aliquots of antibodies were supplemented at the same concentration every 2 days until the tenth day. The cells were then collected for the detection of KIAA1199 expression and the medium for the determination of IL-6 and LMW-HA levels. Thereafter, fresh aliquots of antibodies were supplemented at the same concentration every 2 days until the tenth day. The cells were collected for detecting KIAA1199 expression and the medium for determining the levels of IL-6 and LMW-HA.

### Statistical analysis

The Shapiro–Wilk and Levene methods were used to determine whether the data were normally distributed and the homogeneity of variance, respectively. Differences between two groups of data, which met the normal distribution and homogeneity of variance, were analyzed using the unpaired, two-tailed Student’s *t*-tests. One-way analysis of variance test with the post hoc test (Turkey’s method) was applied to investigate the differences of multiple groups. Graphing was completed using GraphPad Prism version 8.0 (GraphPad Software, Inc., La Jolla, CA) and the data were presented as the mean ± SD.

## Supplementary information

Supplementary Figures and tables

Identified protein by liquid chromatography-tandem mass spectrometry

## Supplementary information

**Supplementary Information** The online version contains supplementary material available at (10.1038/s41419-021-03393-5).
