# Supplementary material for: Secreted KIAA1199 promotes the progression of rheumatoid arthritis by mediating hyaluronic acid degradation in an ANXA1-dependent manner
Source: Cell Death Dis. 2021 Jan 20;12(1):102. doi: 10.1038/s41419-021-03393-5 (PMC7817834; doi:10.1038/s41419-021-03393-5)
Supplement: Supplementary file 1 — Supplementary Figures and tables [file 41419_2021_3393_MOESM1_ESM.docx]

**Supplementary Figures and tables:**


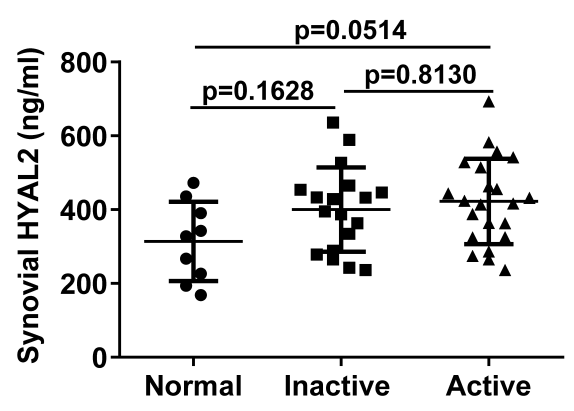


**Figure S1.** The levels of HYAL2 in the synovial fluid of normal subjects (n=9), inactive RA patients (n=18) and active RA patients (n=22).





**Figure S2.** Macroscopic images of paw swelling and X-ray images of toe joints destruction in interphalangeal joints of CIA mice on day 50 before being sacrificed.

**
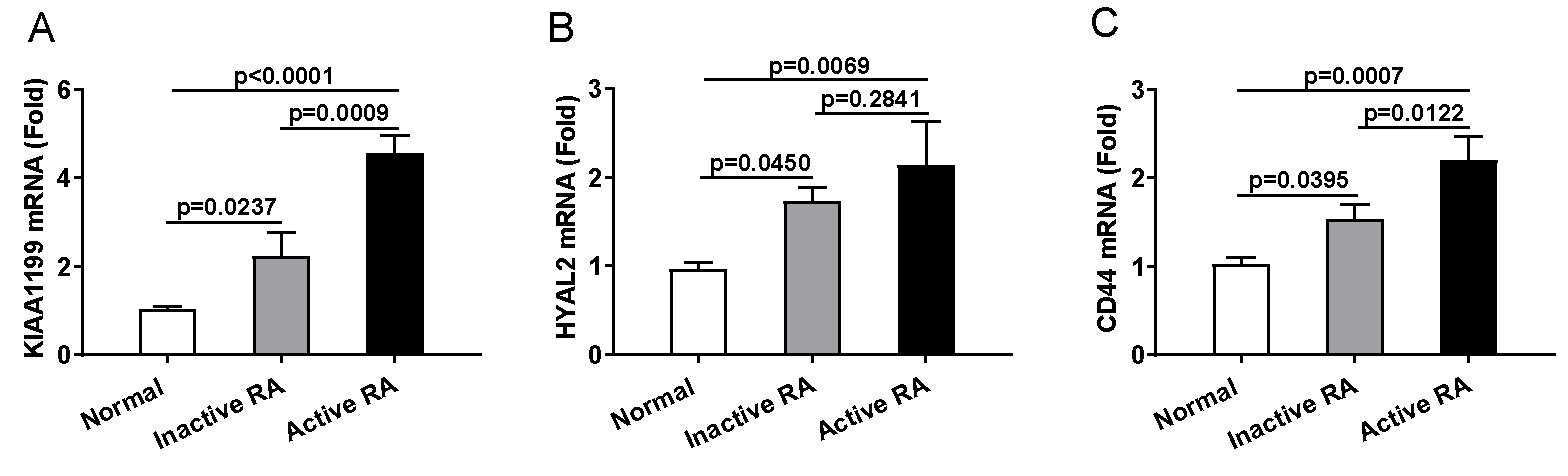
**

**Figure S3.** Relative mRNA expression of *KIAA1199* (A), *HYAL2* (B) and *CD44* (C) genes in synovial tissues from normal subjects (n=5), inactive (n=5) and active RA patients (n=5). GAPDH was used as an internal reference gene for normalization.


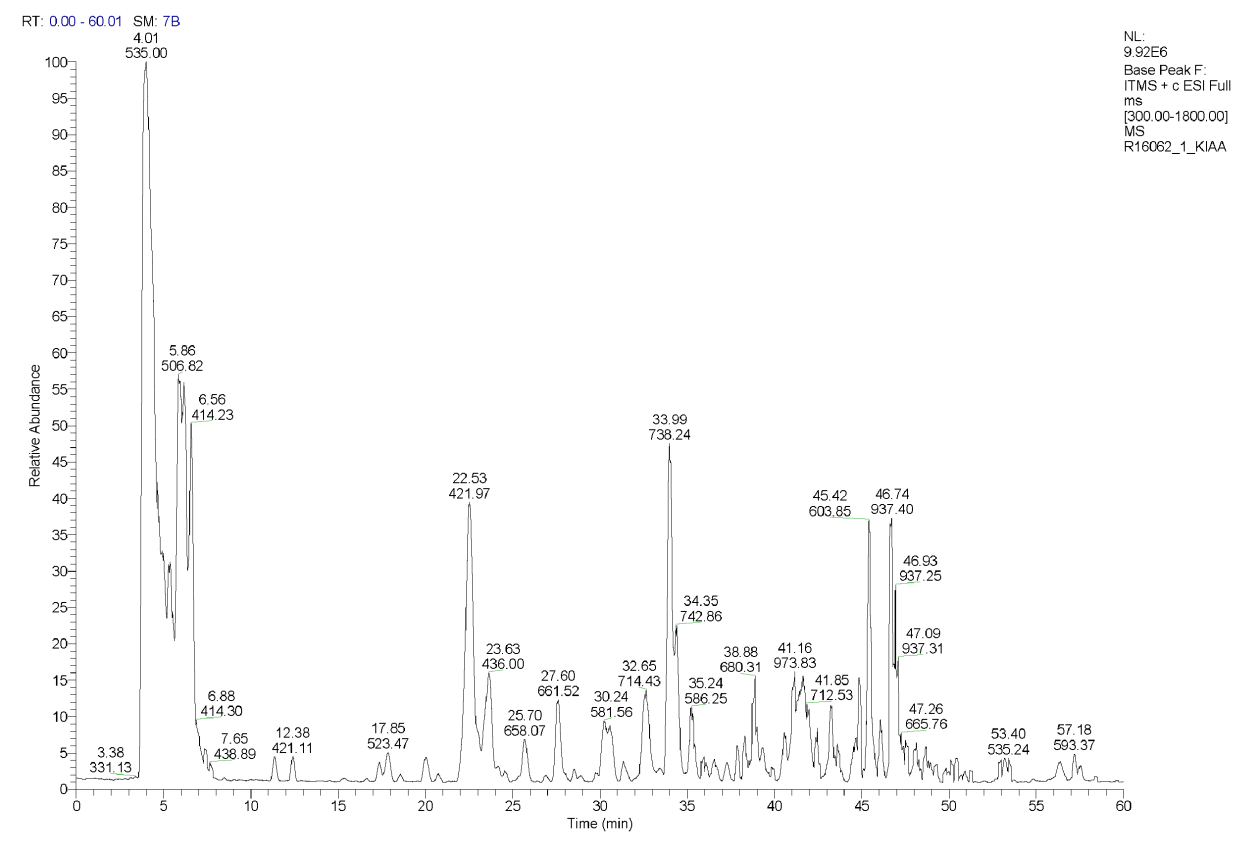


**Figure S4.** Basepeak chromatography from LC-MS/MS analysis of the proteins separated by SDS-PAGE after immunoprecipitation (IP) using KIAA1199 mAb with the membrane protein extracts of RA FLS.


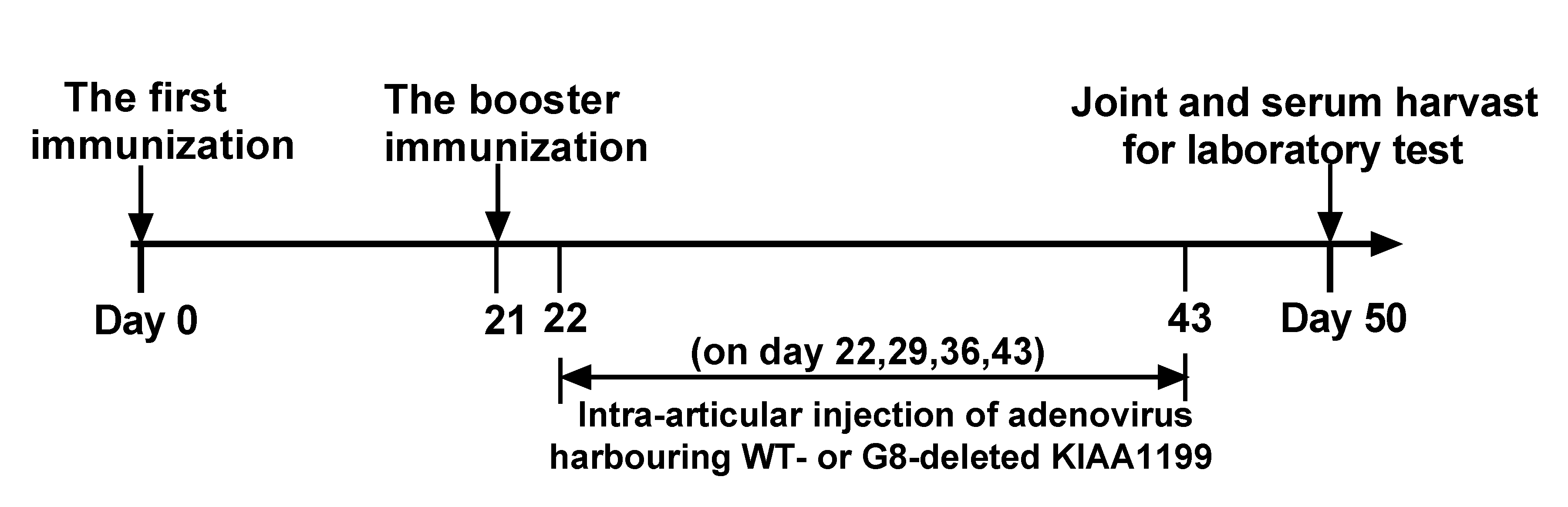


**Figure S5.** Timeline of *Kiaa1199*-KO mice immunization and treatment with adenovirus-coated vectors containing WT-KIAA1199 and ΔG8-KIAA1199 cDNAs.


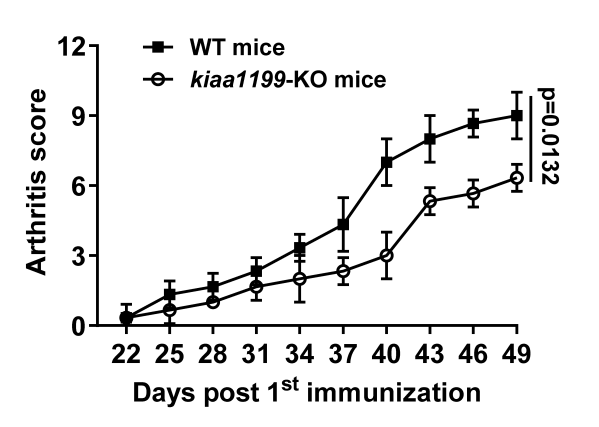


**Figure S6.** The effect of KIAA1199 deficiency on arthritis induction. Compared with WT mice, the arthritis scores of *Kiaa1199*-KO mice were significantly reduced, suggesting an important role of KIAA1199 in the pathological development of arthritis.


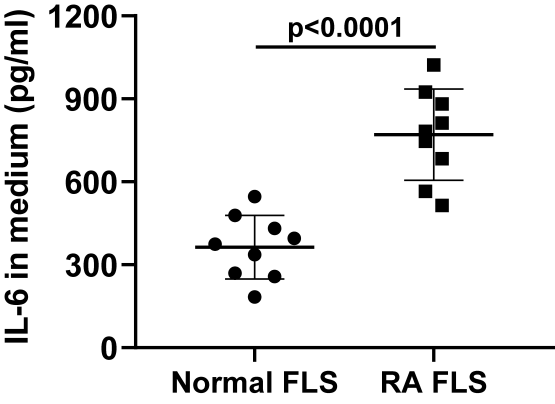


**Figure S7.** Comparison of IL-6 levels in the media of normal FLS and RA FLS cultured for 10 days. Primary FLS were isolated from synovial tissues of healthy controls (n=3) and RA patients (n=3), respectively.

Table S1. Demographic and clinical features of serum samples from normal subjects and RA patients.

|  | **Normal** | **Inactive RA [DNS28<3.2]** | **Active RA**  **[DNS28>3.2]** |
| --- | --- | --- | --- |
| Number, n | 18 | 27 | 33 |
| Age [years] | 52 [21,72] | 58 [38,75] | 56 [29,76] |
| Male/female, n | 6/12 | 5/22 | 9/24 |
| CRP [mg/L] | NA | 4.47 [0.21, 29.6] | 32 [4.23, 140.1] |
| DAS28 score | NA | 2.56 [1.94, 3.01] | 5.02 [3.22, 7.32] |
| ESR [mm/h]  RF positive, n [%]  CCP positive, n [%] | NA  NA  NA | 37.4 [8.4, 105.1]  22 [81]  23 [85] | 46.3 [6.2, 94.9]  27 [82]  29 [88] |
| Duration of disease [years] | NA | 5 [0, 28] | 8 [0, 51] |
| Non-steroidal anti-inflammatory drug usage [%] | NA | NA | NA |
| Disease-modifying anti-rheumatic drug usage [%] | NA | NA | NA |

Values are expressed as median [minimum, maximum] unless stated otherwise.

Abbreviations：RA, rheumatoid arthritis; CRP, C-reactive protein; ESR, erythrocyte sedimentation rate; RF, rheumatoid factor; CCP, [cyclic citrullinated peptide](http://www.baidu.com/link?url=HlsrjSNxCxFmY9q9zv0C1dSUjFWUM6lAHy6aRLVYuLMYtootAsLzAmY-6WF-0ZqTyhrHiXYTQN9ZYLgamlISfNJHuR7EFtklgv14Ra41M6tuXQL180B1Sa8H_UV3N8yk8fAUK7AMlN8ctmyQxL_u4a); NA, not applicable.

Table S2. Demographic and clinical features of synovial fluid from normal subjects and RA patients.

|  | **Normal** | **Inactive RA [DNS28 < 3.2]** | **Active RA**  **[DNS28 > 3.2]** |
| --- | --- | --- | --- |
| Number, n | 9 | 18 | 22 |
| Age [years] | 51 [19,71] | 54 [31, 70] | 57 [21, 72] |
| Male/female, n | 3/6 | 4/14 | 5/17 |
| CRP [mg/L] | NA | 4.08 [0.17, 22.9] | 49 [5.21, 162.4] |
| DAS28 score | NA | 2.73 [1.22, 3.17] | 6.62 [3.21, 6.95] |
| ESR [mm/h]  RF positive, n [%]  CCP positive, n [%] | NA  NA  NA | 28.3 [18.4, 111.7]  14 [78]  15 [83] | 53.8 [19.1, 109.3]  18 [82]  19 [86] |
| Duration of disease [years] | NA | 7 [0, 25] | 9 [0, 45] |
| Non-steroidal Anti-inflammatory drug usage,[%] | NA | NA | NA |
| Disease-modifying anti-rheumatic drug usage, [%] | NA | NA | NA |

Values are expressed as median [minimum, maximum] unless stated otherwise.

Abbreviations：RA, rheumatoid arthritis; CRP, C-reactive protein; ESR, erythrocyte sedimentation rate; RF, rheumatoid factor; CCP, [cyclic citrullinated peptide](http://www.baidu.com/link?url=HlsrjSNxCxFmY9q9zv0C1dSUjFWUM6lAHy6aRLVYuLMYtootAsLzAmY-6WF-0ZqTyhrHiXYTQN9ZYLgamlISfNJHuR7EFtklgv14Ra41M6tuXQL180B1Sa8H_UV3N8yk8fAUK7AMlN8ctmyQxL_u4a); NA, not applicable.

.

Table S3. Sequences of qRT-PCR primers, cDNA primers and shRNA-targeting genes.

| **qRT-PCR** | **Forward (5’→3’)** | **Reverse (5’→3’)** |
| --- | --- | --- |
| *KIAA1199* | GAACCCGGCACATCCTGATT | GATCCGGCTGAATACCTTCATC |
| *HYAL2* | TTCTACCGCGACCGTCTAGG | TGTCCGAATGTAGTGCTCCAC |
| *CD44* | ATGGACAAGTTTTGGTGGCA | AGCGGCCTCCGTCCGAGAGA |
| *ANXA1* | CTAAGCGAAACAATGCACAGC | CCTCCTCAAGGTGACCTGTAA |
| *β-ACTIN* | AGTCATTCCAAATATGAGATGC | TGCTATCACCTCCCCTGTGT |

| **cDNA** | **Forward (5’→3’)** | **Reverse (5’→3’)** |
| --- | --- | --- |
| WT- KIAA1199 | ATGGGAGCTGCTGGGAGGCAGG | CAACTTCTTCTTCTTCACCACAGG |
| ΔC1-KIAA1199 | ATGGGAGCTGCTGGGAGGCAGG | CGTCCCCACGTTGCCACTCTCG |
| ΔC2-KIAA1199 | ATGGGAGCTGCTGGGAGGCAGG | CACGTCTTGAACCCACTCACTGG |
| ΔC3-KIAA1199 | ATGGGAGCTGCTGGGAGGCAGG | AAAATAGCCTCCTTCTGCCAT |
| ΔN1-KIAA1199 | ACCCTTCACCCAGGTGGCATGG | CAACTTCTTCTTCTTCACCACAGG |
| ΔN2-KIAA1199 | AGGCCCAAACTCACAGTCACCAT | CAACTTCTTCTTCTTCACCACAGG |
| ΔN3-KIAA1199 | AGTGGCAACGTGGGGACGGAA | CAACTTCTTCTTCTTCACCACAGG |
| WT-ANXA1 | ATGGCAATGGTATCAGAATTCC | GTTTCCTCCACAAAGAGCCACC |

| **shRNA** | **Target gene sequence (5’→3’)** |
| --- | --- |
| HYAL2 shRNA1 | GCTTAGTGAGATGGACCTCAT |
| HYAL2 shRNA2 | GCATAGTCAAACAGGCACAAT |
| CD44 shRNA1 | GCCCTATTAGTGATTTCCAAA |
| CD44 shRNA2 | CGGAAGTGCTACTTCAGACAA |
| ANXA1 shRNA1 | GCAACCATCATTGACATTCTA |
| ANXA1 shRNA2 | GCCTTGTATGAAGCAGGAGAA |
