# Supplementary material for: Secreted KIAA1199 promotes the progression of rheumatoid arthritis by mediating hyaluronic acid degradation in an ANXA1-dependent manner
Source: Cell Death Dis. 2021 Jan 20;12(1):102. doi: 10.1038/s41419-021-03393-5 (PMC7817834; doi:10.1038/s41419-021-03393-5)
Supplement: Supplementary file 2 — Identified protein by liquid chromatography-tandem mass spectrometry [file 41419_2021_3393_MOESM2_ESM.pdf]

SI. LC-MS/MS identification of proteins isolated from membrane protein extracts of RA FLS by immunoprecipitation using anti-KIAA1199 antibody.

	Reference	PepCount	UniquePepCount	CoverPercent	MW	PI	IdentifiedName							
	File, Scan(s)	Sequence	MH+	Diff(MH+)	Charge	Rank	XC	DeltaCn	Sp	RSp	Ions	PI	GroupCount	ProteinCount
$1-1		30	11	34.81%	38604.07	7.57	sp|P07355|ANXA2_HUMAN Annexin A2 OS=Homo sapiens GN=ANXA2 PE=1 SV=2							
$1-2		30	11	34.81%	38604.07	7.57	tr|A0A024R5Z7|A0A024R5Z7_HUMAN Annexin OS=Homo sapiens GN=ANXA2 PE=3 SV=1							
$1-3		30	11	34.81%	38576.02	7.57	tr|V9HW65|V9HW65_HUMAN Annexin OS=Homo sapiens GN=HEL-S-270 PE=2 SV=1							
	R16062_1_KIAA,13942	K.ALLYLCGGDD.-	1097.1939	0.6879	1	1	1.9013	0.3076	748	1	11|18	3.89	1	3
	R16062_1_KIAA,8110	K.AYTNFDAER.D	1087.1242	-1.6758	2	1	2.268	0.2961	430.9	1	11|16	4.37	1	3
	R16062_1_KIAA,11816	K.DIISDTSGDFR.K	1226.275	-0.646	2	1	3.1576	0.3734	832	1	14|20	3.93	1	3
	R16062_1_KIAA,11828	K.DIISDTSGDFR.K	1226.275	-0.752	2	1	3.1305	0.4622	859	1	14|20	3.93	1	3
	R16062_1_KIAA,12014	K.DIISDTSGDFR.K	1226.275	0.962	2	1	2.3881	0.3634	950.2	1	14|20	3.93	1	3
	R16062_1_KIAA,17845	K.GLGTDEDSLIEIICSR.T	1778.9331	-1.3739	2	1	3.8729	0.4989	674.4	1	15|30	3.92	1	3
	R16062_1_KIAA,17856	K.GLGTDEDSLIEIICSR.T	1778.9331	-1.6389	2	1	3.8725	0.4986	650.9	1	14|30	3.92	1	3
	R16062_1_KIAA,17899	K.GLGTDEDSLIEIICSR.T	1778.9331	0.5091	2	1	2.5057	0.3859	1162.9	1	18|30	3.92	1	3
	R16062_1_KIAA,17910	K.GLGTDEDSLIEIICSR.T	1778.9331	-0.6069	2	1	3.8163	0.5738	1600.7	1	20|30	3.92	1	3
	R16062_1_KIAA,17932	K.GLGTDEDSLIEIICSR.T	1778.9331	0.5531	2	1	2.7768	0.4418	1336.4	1	20|30	3.92	1	3
	R16062_1_KIAA,17943	K.GLGTDEDSLIEIICSR.T	1778.9331	0.7111	2	1	2.5817	0.305	802.4	1	15|30	3.92	1	3
	R16062_1_KIAA,17804	K.GVDEVTIVNILTNR.S	1543.7474	0.7804	2	1	3.2549	0.4453	1744.9	1	19|26	4.37	1	3
	R16062_1_KIAA,17812	K.GVDEVTIVNILTNR.S	1543.7474	0.0924	2	1	2.6743	0.3706	851.4	1	15|26	4.37	1	3
	R16062_1_KIAA,17836	K.GVDEVTIVNILTNR.S	1543.7474	1.3014	2	1	2.8155	0.4081	989.8	1	17|26	4.37	1	3
	R16062_1_KIAA,17880	K.GVDEVTIVNILTNR.S	1543.7474	0.3204	3	1	4.1475	0.4096	1653.2	1	28|52	4.37	1	3
	R16062_1_KIAA,17	K.GVDEVTIVNILTNR.S	1543.7474	1.9384	2	1	3.1321	0.4616	1645.9	1	19|26	4.37	1	3

	907															
	R16062_1_KIAA,12907	K.SLYYYIQQDTK.G		1422.5639	-0.9931	2	1		2.3264	0.3449	1104	1	14|20	5.55	1	3
	R16062_1_KIAA,12916	K.SLYYYIQQDTK.G		1422.5639	0.4649	2	1		2.367	0.4125	640.5	1	13|20	5.55	1	3
	R16062_1_KIAA,12927	K.SLYYYIQQDTK.G		1422.5639	0.4079	2	1		2.8713	0.4141	910.4	1	14|20	5.55	1	3
	R16062_1_KIAA,13882	R.AEDGSVIDYELIDQDAR.D		1909.9855	-0.7465	2	1		2.751	0.4331	519.3	1	15|32	3.66	1	3
	R16062_1_KIAA,13907	R.AEDGSVIDYELIDQDAR.D		1909.9855	-0.2795	2	1		3.2203	0.5254	816.7	1	19|32	3.66	1	3
	R16062_1_KIAA,13918	R.AEDGSVIDYELIDQDAR.D		1909.9855	1.3425	2	1		3.1439	0.4932	830	1	19|32	3.66	1	3
	R16062_1_KIAA,13931	R.AEDGSVIDYELIDQDAR.D		1909.9855	1.2355	2	1		3.4189	0.4991	1711.7	1	22|32	3.66	1	3
	R16062_1_KIAA,12171	R.DALNIETAIK.T		1088.2367	0.3977	2	1		2.7248	0.3407	850.2	1	13|18	4.37	1	3
	R16062_1_KIAA,12308	R.DALNIETAIK.T		1088.2367	2.0377	2	1		2.3828	0.341	890.7	1	14|18	4.37	1	3
	R16062_1_KIAA,10430	R.QDIAFAYQR.R		1112.22	0.926	2	1		2.2605	0.3212	519.2	1	12|16	5.84	1	3
	R16062_1_KIAA,13087	R.RAEDGSVIDYELIDQDAR.D		2066.1718	0.9038	2	1		2.6999	0.4013	497.8	1	16|34	3.96	1	3
	R16062_1_KIAA,13134	R.RAEDGSVIDYELIDQDAR.D		2066.1718	1.2268	2	1		2.6453	0.3437	680.5	1	17|34	3.96	1	3
	R16062_1_KIAA,7570	R.TNQELQEINR.V		1245.3247	0.7747	2	1		2.6509	0.2728	1332.5	1	15|18	4.53	1	3
	R16062_1_KIAA,7581	R.TNQELQEINR.V		1245.3247	0.7117	2	1		2.6962	0.2478	1197.8	1	15|18	4.53	1	3
$2-1			25	9	37.81%	35936.83	4.94	sp|P08758|ANXA5_HUMAN Annexin A5 OS=Homo sapiens GN=ANXA5 PE=1 SV=2								
$2-2			25	9	37.81%	35936.83	4.94	tr|V9HWE0|V9HWE0_HUMAN Annexin OS=Homo sapiens GN=HEL-S-7 PE=2 SV=1								
	R16062_1_KIAA,15102 - 15103	K.FITIFGTR.S		955.1352	0.0882	2	1		2.3288	0.305	380.9	12	10|14	9.75	1	2
	R16062_1_KIAA,15111	K.FITIFGTR.S		955.1352	0.0352	2	1		2.4588	0.2339	655.1	4	12|14	9.75	1	2
	R16062_1_KIAA,19682	K.GLGTDEESILTLLTSR.S		1705.8887	0.3327	2	1		2.6333	0.4899	611.1	1	13|30	4.14	1	2
	R16062_1_KIAA,19692	K.GLGTDEESILTLLTSR.S		1705.8887	0.5297	2	1		3.4593	0.5461	1284.2	1	18|30	4.14	1	2

R16062_1_KIAA,19760	K.GLGTDEESILTLLTSR.S	1705.8887	2.0777	2	1	3.5778	0.5109	1731.9	1 23|30	4.14	1	2
R16062_1_KIAA,15030	K.NFATSLYSM*IK.G	1291.4984	0.3774	2	1	2.5078	0.1523	1191.1	1 15|20	8.59	1	2
R16062_1_KIAA,15080	K.NFATSLYSM*IK.G	1291.4984	0.4904	2	1	2.4604	0.239	1083.7	1 14|20	8.59	1	2
R16062_1_KIAA,15085	K.NFATSLYSM*IK.G	1291.4984	0.3154	2	1	2.2482	0.2837	720.2	1 12|20	8.59	1	2
R16062_1_KIAA,10788	K.VLTEIIASR.T	1002.1901	0.9011	2	1	2.6054	0.3803	1383.3	1 14|16	5.97	1	2
R16062_1_KIAA,10795	K.VLTEIIASR.T	1002.1901	0.8571	2	1	2.6476	0.318	1293.5	1 14|16	5.97	1	2
R16062_1_KIAA,19046	R.DLLDDLKSELTGK.F	1447.6132	0.6192	2	1	2.6604	0.3643	1284.5	1 17|24	4.23	1	2
R16062_1_KIAA,19061	R.DLLDDLKSELTGK.F	1447.6132	-0.4418	2	1	2.695	0.3463	616.1	1 14|24	4.23	1	2
R16062_1_KIAA,19071	R.DLLDDLKSELTGK.F	1447.6132	0.8272	2	1	2.5829	0.3049	1109	1 17|24	4.23	1	2
R16062_1_KIAA,18936	R.DPDAGIDEAQVEQDAQALFQAGELK.W	2659.8007	1.1797	3	1	4.6434	0.3989	1411	1 32|96	3.62	1	2
R16062_1_KIAA,18947	R.DPDAGIDEAQVEQDAQALFQAGELK.W	2659.8007	-0.7993	3	1	4.8615	0.5447	1131.9	1 30|96	3.62	1	2
R16062_1_KIAA,18950	R.DPDAGIDEAQVEQDAQALFQAGELK.W	2659.8007	1.0667	3	1	4.1575	0.4387	1226.4	1 31|96	3.62	1	2
R16062_1_KIAA,19000	R.DPDAGIDEAQVEQDAQALFQAGELK.W	2659.8007	0.2617	2	1	4.5825	0.6553	1949.4	1 25|48	3.62	1	2
R16062_1_KIAA,19016	R.DPDAGIDEAQVEQDAQALFQAGELK.W	2659.8007	1.1667	2	1	2.9861	0.5305	1076.4	1 21|48	3.62	1	2
R16062_1_KIAA,20298	R.ETSGNLEQLLLAVVK.S	1614.8655	0.3715	2	1	2.9096	0.4155	606.5	1 16|28	4.53	1	2
R16062_1_KIAA,20309	R.ETSGNLEQLLLAVVK.S	1614.8655	0.6735	2	1	2.8967	0.3955	905.3	1 15|28	4.53	1	2
R16062_1_KIAA,20471	R.ETSGNLEQLLLAVVK.S	1614.8655	2.4365	2	1	2.8476	0.4	794.1	1 15|28	4.53	1	2
R16062_1_KIAA,15711	R.SEIDLFNIR.K	1107.2417	0.0697	2	1	2.5099	0.1854	983.2	4 13|16	4.37	1	2
R16062_1_KIAA,15721	R.SEIDLFNIR.K	1107.2417	0.2717	2	1	2.5782	0.1865	1063	3 14|16	4.37	1	2
R16062_1_KIAA,18256	R.SIPAYLAETLYYAM*K.G	1751.0361	-0.2039	2	1	2.7881	0.4688	577.4	1 16|28	5.72	1	2
R16062_1_KIAA,18	R.SIPAYLAETLYYAM*K.G	1751.0361	-0.1329	2	1	2.7931	0.5509	423.1	1 16|28	5.72	1	2

265

$3-1	38	8	36.67%	36105.84	8.46	sp|P01857|IGHG1_HUMAN Ig gamma-1 chain C region OS=Homo sapiens GN=IGHG1 PE=1 SV=1
$3-2	38	8	25.47%	52426.11	8.45	tr|A0A087WYC5|A0A087WYC5_HUMAN Ig gamma-1 chain C region OS=Homo sapiens GN=IGHG1 PE=1 SV=1
$3-3	38	8	25.58%	51912.59	8.33	tr|A0A087X079|A0A087X079_HUMAN Ig gamma-1 chain C region OS=Homo sapiens GN=IGHG1 PE=1 SV=1
$3-4	38	8	25.97%	51153.84	7.49	tr|A0A087WV47|A0A087WV47_HUMAN Ig gamma-1 chain C region OS=Homo sapiens GN=IGHG1 PE=1 SV=1
$3-5	38	8	30.33%	43911.84	6.55	tr|A0A0A0MS08|A0A0A0MS08_HUMAN Ig gamma-1 chain C region (Fragment) OS=Homo sapiens GN=IGHG1 PE=1 SV=1
$3-6	38	8	26.19%	50488.11	8.7	tr|A0A087X1C7|A0A087X1C7_HUMAN Ig gamma-1 chain C region OS=Homo sapiens GN=IGHG1 PE=1 SV=1
$3-7	38	8	25.64%	51596.34	8.44	tr|A8K008|A8K008_HUMAN cDNA FLJ78387 OS=Homo sapiens PE=1 SV=1
$3-8	38	8	26.02%	51082.9	8.91	tr|Q6GMX6|Q6GMX6_HUMAN IGH@ protein OS=Homo sapiens GN=IGH@ PE=1 SV=1
$3-9	38	8	25.47%	52362.37	8.64	tr|Q5EFE5|Q5EFE5_HUMAN Anti-RhD monoclonal T125 gamma1 heavy chain OS=Homo sapiens PE=2 SV=1
$3-10	38	8	25.64%	51724.53	8.14	tr|Q6N089|Q6N089_HUMAN Putative uncharacterized protein DKFZp686P15220 OS=Homo sapiens GN=DKFZp686P15220 PE=1 SV=1
$3-11	38	8	25.97%	50870.45	7.89	tr|S6B291|S6B291_HUMAN IgG H chain OS=Homo sapiens PE=1 SV=1
$3-12	38	8	25.74%	51715.46	7.88	tr|V9HW68|V9HW68_HUMAN Epididymis luminal protein 214 OS=Homo sapiens GN=HEL-214 PE=2 SV=1
$3-13	38	8	34.77%	38162.08	8.27	tr|Q6PYX1|Q6PYX1_HUMAN Hepatitis B virus receptor binding protein (Fragment) OS=Homo sapiens PE=1 SV=1
$3-14	38	8	25.10%	52852	8.75	tr|Q7Z351|Q7Z351_HUMAN Putative uncharacterized protein DKFZp686N02209 OS=Homo sapiens GN=DKFZp686N02209 PE=2 SV=1
$3-15	38	8	25.21%	52612.6	8.46	tr|Q6N094|Q6N094_HUMAN Putative uncharacterized protein DKFZp686O01196 OS=Homo sapiens GN=DKFZp686O01196 PE=2 SV=1
$3-16	38	8	26.08%	50822.56	8.45	tr|A0A087X010|A0A087X010_HUMAN Ig gamma-1 chain C region OS=Homo sapiens GN=IGHG1 PE=1 SV=1
$3-17	38	8	25.47%	52109.86	7.49	tr|A0A087WYE1|A0A087WYE1_HUMAN Ig gamma-1 chain C region OS=Homo sapiens GN=IGHG1 PE=1 SV=1
$3-18	38	8	25.47%	52042.86	8.31	tr|Q6MZQ6|Q6MZQ6_HUMAN Putative uncharacterized protein DKFZp686G11190 OS=Homo sapiens GN=DKFZp686G11190 PE=2 SV=1

$3-19			38	8	25.16%	52758.77	8.75	tr|Q6N097|Q6N097_HUMAN Putative uncharacterized protein DKFZp686H20196 OS=Homo sapiens GN=DKFZp686H20196 PE=2 SV=1							
$3-20			38	8	26.08%	50915.61	8.13	tr|A0A0G2JNK4|A0A0G2JNK4_HUMAN Ig gamma-1 chain C region OS=Homo sapiens GN=IGHG1 PE=1 SV=1							
$3-21			38	8	25.97%	50926.64	8.33	tr|Q6N096|Q6N096_HUMAN Putative uncharacterized protein DKFZp686I15196 OS=Homo sapiens GN=DKFZp686I15196 PE=2 SV=1							
$3-22			38	8	25.58%	52120.99	7.51	tr|Q6MZV7|Q6MZV7_HUMAN Putative uncharacterized protein DKFZp686C11235 OS=Homo sapiens GN=DKFZp686C11235 PE=2 SV=1							
	R16062_1_KIAA,13032	K.FNWYVDGVEVHNAK.T		1678.8284	0.0084	2	1		2.4796	0.361	762.4	1 16|26	5.32	1	22
	R16062_1_KIAA,13037	K.FNWYVDGVEVHNAK.T		1678.8284	0.3344	2	1		2.5702	0.4037	960.2	1 17|26	5.32	1	22
	R16062_1_KIAA,13079	K.FNWYVDGVEVHNAK.T		1678.8284	2.0254	2	1		2.3047	0.4172	489.5	1 13|26	5.32	1	22
	R16062_1_KIAA,13434	K.FNWYVDGVEVHNAK.T		1678.8284	1.4904	2	1		2.6719	0.4252	865.7	1 16|26	5.32	1	22
	R16062_1_KIAA,13450	K.FNWYVDGVEVHNAK.T		1678.8284	-0.5846	2	1		3.5811	0.5896	1005.1	1 16|26	5.32	1	22
	R16062_1_KIAA,13471	K.FNWYVDGVEVHNAK.T		1678.8284	0.6224	2	1		3.0847	0.4418	959.7	1 17|26	5.32	1	22
	R16062_1_KIAA,13499	K.FNWYVDGVEVHNAK.T		1678.8284	0.4734	2	1		2.6174	0.4358	586.3	1 13|26	5.32	1	22
	R16062_1_KIAA,15732	K.GFYPSDIAVEWESNGQPENNYK.T		2545.6579	-0.8321	2	1		3.237	0.3584	1188.7	1 21|42	4	3	26
	R16062_1_KIAA,15744	K.GFYPSDIAVEWESNGQPENNYK.T		2545.6579	0.1459	2	1		3.2157	0.376	694.7	1 19|42	4	3	26
	R16062_1_KIAA,15820	K.GFYPSDIAVEWESNGQPENNYK.T		2545.6579	1.1829	2	1		4.0896	0.5161	1541.9	1 23|42	4	3	26
	R16062_1_KIAA,15886	K.GFYPSDIAVEWESNGQPENNYK.T		2545.6579	-2.8691	2	1		3.3734	0.4336	1416.4	1 20|42	4	3	26
	R16062_1_KIAA,15897	K.GFYPSDIAVEWESNGQPENNYK.T		2545.6579	1.2169	2	1		3.1724	0.4151	814	1 20|42	4	3	26
	R16062_1_KIAA,15997	K.GFYPSDIAVEWESNGQPENNYK.T		2545.6579	-2.8691	2	1		2.9126	0.2407	646.2	1 17|42	4	3	26
	R16062_1_KIAA,16239	K.GFYPSDIAVEWESNGQPENNYK.T		2545.6579	-1.8471	2	1		2.6677	0.46	736.1	1 20|42	4	3	26
	R16062_1_KIAA,12489	K.NQVSLTCLVK.G		1162.3554	1.1304	2	1		2.3778	0.3172	753.4	1 12|18	8.22	5	34

R16062_1_KIAA,11035	K.STSGGTAALGCLVK.D	1322.484	0.516	2	1	2.6657	0.4955	841.2	1 15|26	7.94	3	30
R16062_1_KIAA,11046	K.STSGGTAALGCLVK.D	1322.484	0.393	2	1	2.4749	0.4182	1320.9	1 19|26	7.94	3	30
R16062_1_KIAA,17998	K.THTCPPCPAPELLGGPSVFLFPPK.P	2621.0136	-0.2134	3	1	4.3027	0.4413	1646.7	1 36|92	6.41	2	23
R16062_1_KIAA,18001	K.THTCPPCPAPELLGGPSVFLFPPK.P	2621.0136	2.4746	3	1	4.4616	0.4912	1449.2	1 36|92	6.41	2	23
R16062_1_KIAA,18155	K.THTCPPCPAPELLGGPSVFLFPPK.P	2621.0136	2.1636	3	1	4.1152	0.5475	1058.3	1 30|92	6.41	2	23
R16062_1_KIAA,18274	K.THTCPPCPAPELLGGPSVFLFPPK.P	2621.0136	1.4206	3	1	4.5413	0.3761	1462.8	1 36|92	6.41	2	23
R16062_1_KIAA,16712	K.THTCPPCPAPELLGGPSVFLFPPKPK.D	2846.3022	2.8522	3	1	5.2416	0.5911	1083.5	1 39|100	7.76	2	23
R16062_1_KIAA,16756	K.THTCPPCPAPELLGGPSVFLFPPKPK.D	2846.3022	2.3592	3	1	3.8943	0.4899	1037.1	1 35|100	7.76	2	23
R16062_1_KIAA,16865	K.THTCPPCPAPELLGGPSVFLFPPKPK.D	2846.3022	-2.3138	3	1	4.4267	0.5224	706	1 31|100	7.76	2	23
R16062_1_KIAA,12985	R.TPEVTCVVVDVSHEDPEVK.F	2140.3272	-0.5408	2	1	2.367	0.3959	560.2	1 15|36	4.17	2	23
R16062_1_KIAA,12995	R.TPEVTCVVVDVSHEDPEVK.F	2140.3272	1.0042	2	1	2.4457	0.3006	555.8	1 15|36	4.17	2	23
R16062_1_KIAA,13007	R.TPEVTCVVVDVSHEDPEVK.F	2140.3272	-1.3858	2	1	2.6724	0.403	851.8	1 17|36	4.17	2	23
R16062_1_KIAA,13013	R.TPEVTCVVVDVSHEDPEVK.F	2140.3272	1.3762	2	1	2.9498	0.5184	1221.1	1 21|36	4.17	2	23
R16062_1_KIAA,13066	R.TPEVTCVVVDVSHEDPEVK.F	2140.3272	2.6872	3	1	3.7958	0.4648	1593.5	1 34|72	4.17	2	23
R16062_1_KIAA,13440	R.TPEVTCVVVDVSHEDPEVK.F	2140.3272	1.0192	2	1	3.0336	0.569	1086.9	1 20|36	4.17	2	23
R16062_1_KIAA,18132	R.VVSVLTVLHQDWLNGK.E	1809.1018	1.1018	2	1	2.4514	0.4444	961.7	1 16|30	6.71	3	31
R16062_1_KIAA,18153	R.VVSVLTVLHQDWLNGK.E	1809.1018	0.7948	2	1	2.5641	0.4073	1008.1	1 17|30	6.71	3	31
R16062_1_KIAA,18221	R.VVSVLTVLHQDWLNGK.E	1809.1018	1.9848	2	1	3.2725	0.5713	1812.9	1 21|30	6.71	3	31
R16062_1_KIAA,18258	R.VVSVLTVLHQDWLNGK.E	1809.1018	2.1968	2	1	3.2131	0.5276	1326.1	1 19|30	6.71	3	31
R16062_1_KIAA,18273	R.VVSVLTVLHQDWLNGK.E	1809.1018	-2.5362	2	1	4.0174	0.5921	1429.8	1 20|30	6.71	3	31
R16062_1_KIAA,18	R.VVSVLTVLHQDWLNGK.E	1809.1018	-2.4042	2	1	3.7172	0.4354	994.5	1 18|30	6.71	3	31

	295														
	R16062_1_KIAA,18570	R.VVSVLTVLHQDWLNGK.E		1809.1018	-1.3862	2	1		2.8304	0.5929	1186.7	1 18|30	6.71	3	31
	R16062_1_KIAA,18605	R.VVSVLTVLHQDWLNGK.E		1809.1018	-1.1742	2	1		3.1467	0.5284	1147	1 18|30	6.71	3	31
$4-1			16	8	31.79%	38714.23	6.57	sp|P04083|ANXA1_HUMAN Annexin A1 OS=Homo sapiens GN=ANXA1 PE=1 SV=2							
$4-2			16	8	31.79%	38714.23	6.57	tr|Q5TZZ9|Q5TZZ9_HUMAN Annexin OS=Homo sapiens GN=ANXA1 PE=2 SV=1							
	R16062_1_KIAA,11072	K.AAYLQETGKPLDETLK.K		1777.9965	0.7785	2	1		2.2334	0.2431	407.4	1 12|30	4.68	1	2
	R16062_1_KIAA,19230	K.GLGTDEDTLIEILASR.T		1703.8728	-0.4642	2	1		3.0458	0.3822	2358.2	1 21|30	3.92	1	2
	R16062_1_KIAA,19244	K.GLGTDEDTLIEILASR.T		1703.8728	-1.5622	2	1		4.663	0.4276	2287.6	1 24|30	3.92	1	2
	R16062_1_KIAA,19263	K.GLGTDEDTLIEILASR.T		1703.8728	0.3618	2	1		3.1859	0.4203	2091.8	1 22|30	3.92	1	2
	R16062_1_KIAA,19274	K.GLGTDEDTLIEILASR.T		1703.8728	1.0718	2	1		3.6598	0.4439	2714.9	1 24|30	3.92	1	2
	R16062_1_KIAA,19410	K.GLGTDEDTLIEILASR.T		1703.8728	2.0948	2	1		3.1506	0.4943	2114.5	1 21|30	3.92	1	2
	R16062_1_KIAA,16632	K.GTDVNVFNTILTTR.S		1551.7263	-1.3467	2	1		3.1184	0.4131	1079.1	1 16|26	5.84	1	2
	R16062_1_KIAA,16637	K.GTDVNVFNTILTTR.S		1551.7263	-0.0637	2	1		3.1364	0.5202	858.9	1 16|26	5.84	1	2
	R16062_1_KIAA,16658	K.GTDVNVFNTILTTR.S		1551.7263	-1.5257	2	1		2.7999	0.2563	739.8	1 15|26	5.84	1	2
	R16062_1_KIAA,16823	K.GTDVNVFNTILTTR.S		1551.7263	-0.3867	2	1		3.1829	0.3685	921.1	1 18|26	5.84	1	2
	R16062_1_KIAA,16846	K.GTDVNVFNTILTTR.S		1551.7263	-0.4627	2	1		3.3369	0.5122	847	1 14|26	5.84	1	2
	R16062_1_KIAA,18607	K.GVDEATIIDILTK.R		1388.5889	0.7019	2	1		2.4661	0.4782	627	1 14|24	4.03	1	2
	R16062_1_KIAA,12033	K.ILVALCGGN.-		917.0787	0.1937	2	1		2.2145	0.3543	685.1	1 12|16	5.52	1	2
	R16062_1_KIAA,16365	K.M*YGISLCQAILDETK.G		1759.0084	-0.4496	2	1		3.682	0.5894	1091.1	1 18|28	4.37	1	2
	R16062_1_KIAA,10521	K.TPAQFDADELR.A		1263.3385	2.0065	2	1		2.3454	0.4779	968.8	1 14|20	4.03	1	2
	R16062_1_KIAA,11168	R.SEDFGVNEDLADSDAR.A		1740.7205	0.6615	2	1		3.011	0.4607	1450.8	1 20|30	3.66	1	2

$5-1			12	8	70.75%	15998.35	6.74	sp|P68871|HBB_HUMAN Hemoglobin subunit beta OS=Homo sapiens GN=HBB PE=1 SV=2							
$5-2			12	8	70.75%	15998.35	6.74	tr|D9YZU5|D9YZU5_HUMAN Hemoglobin, beta OS=Homo sapiens GN=HBB PE=3 SV=1							
	R16062_1_KIAA,10178	K.EFTPPVQAAYQK.V		1379.5423	-0.5657	2	1		2.2229	0.1504	464.9	1 14|22	6.1	1	2
	R16062_1_KIAA,11116	K.GTFATLSELHCDK.L		1479.6104	-1.6406	2	1		2.3194	0.3027	305.5	2 10|24	5.32	3	4
	R16062_1_KIAA,11123	K.GTFATLSELHCDK.L		1479.6104	0.3594	2	1		2.3893	0.4349	434.2	1 12|24	5.32	3	4
	R16062_1_KIAA,12498	K.SAVTALWGK.V		933.0864	0.8684	2	1		2.5905	0.4231	810.7	1 13|16	8.47	3	4
	R16062_1_KIAA,12521	K.SAVTALWGK.V		933.0864	0.7164	2	1		2.3857	0.3995	1095.1	1 14|16	8.47	3	4
	R16062_1_KIAA,14892	K.VLGAFSDGLAHLDNLK.G		1670.8908	-0.3942	2	1		2.7099	0.1644	1055.4	1 19|30	5.21	3	5
	R16062_1_KIAA,10277	K.VNVDEVGGEALGR.L		1315.415	0.762	2	1		2.9846	0.1859	951.5	1 15|24	4.14	2	3
	R16062_1_KIAA,10707	K.VNVDEVGGEALGR.L		1315.415	0.086	2	1		3.2653	0.2788	1280.3	1 18|24	4.14	2	3
	R16062_1_KIAA,10734	K.VNVDEVGGEALGR.L		1315.415	1.099	2	1		2.6907	0.1598	1094.6	1 17|24	4.14	2	3
	R16062_1_KIAA,8549	K.VVAGVANALAHK.Y		1150.3561	0.2381	2	1		2.3422	0.4164	674.9	1 13|22	8.73	2	4
	R16062_1_KIAA,15017	R.FFESFGDLSTPDAVM*GNPK.V		2076.2717	-2.1273	2	1		2.478	0.3888	294.4	1 13|36	4.03	3	4
	R16062_1_KIAA,15697	R.LLVVYPWTQR.F		1275.5235	0.4625	2	1		2.2234	0.3052	665.8	1 11|18	8.75	4	6
$6-1			32	6	41.00%	26234.51	8.24	tr|Q8TCD0|Q8TCD0_HUMAN Uncharacterized protein OS=Homo sapiens PE=1 SV=1							
$6-2			32	6	44.75%	24029.93	8.29	tr|Q0KKI6|Q0KKI6_HUMAN Immunoblobulin light chain (Fragment) OS=Homo sapiens PE=1 SV=1							
	R16062_1_KIAA,16535	-.TVAAPSVFIFPPSDEQLK.S		1947.22	-1.248	2	1		2.5905	0.4133	292.4	1 13|34	4.37	4	8
	R16062_1_KIAA,16557	-.TVAAPSVFIFPPSDEQLK.S		1947.22	-1.181	2	1		2.2549	0.2349	229.1	1 13|34	4.37	4	8
	R16062_1_KIAA,16777	-.TVAAPSVFIFPPSDEQLK.S		1947.22	0.782	2	1		2.4042	0.3821	524.6	1 19|34	4.37	4	8
	R16062_1_KIAA,16788	-.TVAAPSVFIFPPSDEQLK.S		1947.22	0.82	2	1		2.4522	0.5211	388.3	1 16|34	4.37	4	8
	R16062_1_KIAA,17	-.TVAAPSVFIFPPSDEQLK.S		1947.22	0.531	2	1		2.9491	0.4563	756.2	1 21|34	4.37	4	8

195 - 17196												
R16062_1_KIAA,17206	-.TVAAPSVFIFPPSDEQLK.S	1947.22	0.674	2	1	2.3807	0.3829	237.9	1 13|34	4.37	4	8
R16062_1_KIAA,17485	-.TVAAPSVFIFPPSDEQLK.S	1947.22	1.737	2	1	2.6139	0.4106	390	1 16|34	4.37	4	8
R16062_1_KIAA,13302	K.DSTYSLSSTLTLSK.A	1503.6336	0.9196	2	1	2.7028	0.4304	903.4	1 16|26	5.84	3	6
R16062_1_KIAA,13312	K.DSTYSLSSTLTLSK.A	1503.6336	0.2906	2	1	2.6194	0.4473	718.5	1 15|26	5.84	3	6
R16062_1_KIAA,16001	K.SGTASVVCLLNNFYPR.E	1799.0141	0.3261	2	1	2.9192	0.5415	550.6	1 13|30	7.94	4	8
R16062_1_KIAA,16033	K.SGTASVVCLLNNFYPR.E	1799.0141	0.1381	2	1	3.312	0.4871	1199.2	1 18|30	7.94	4	8
R16062_1_KIAA,17303	K.SGTASVVCLLNNFYPR.E	1799.0141	-0.3619	2	1	2.4085	0.4512	580.2	1 14|30	7.94	4	8
R16062_1_KIAA,17350	K.SGTASVVCLLNNFYPR.E	1799.0141	-0.1429	2	1	2.8188	0.4831	927.2	1 15|30	7.94	4	8
R16062_1_KIAA,18804	K.SGTASVVCLLNNFYPR.E	1799.0141	1.6411	2	1	2.9725	0.5376	1033.7	1 17|30	7.94	4	8
R16062_1_KIAA,18845	K.SGTASVVCLLNNFYPR.E	1799.0141	0.5641	2	1	2.4368	0.4778	1164.4	1 18|30	7.94	4	8
R16062_1_KIAA,18876	K.SGTASVVCLLNNFYPR.E	1799.0141	2.0111	2	1	2.9342	0.4613	1195.6	1 18|30	7.94	4	8
R16062_1_KIAA,18922	K.SGTASVVCLLNNFYPR.E	1799.0141	-2.5899	2	1	3.8096	0.4571	1228.7	1 17|30	7.94	4	8
R16062_1_KIAA,19029	K.SGTASVVCLLNNFYPR.E	1799.0141	-2.6549	2	1	3.1649	0.3967	843.2	1 15|30	7.94	4	8
R16062_1_KIAA,19259	K.SGTASVVCLLNNFYPR.E	1799.0141	0.8761	2	1	2.7348	0.4147	845.6	1 15|30	7.94	4	8
R16062_1_KIAA,19266	K.SGTASVVCLLNNFYPR.E	1799.0141	-0.5639	2	1	3.5693	0.5112	830.8	1 16|30	7.94	4	8
R16062_1_KIAA,19342	K.SGTASVVCLLNNFYPR.E	1799.0141	0.4391	2	1	3.2765	0.4663	1053.3	1 17|30	7.94	4	8
R16062_1_KIAA,19358	K.SGTASVVCLLNNFYPR.E	1799.0141	0.4801	2	1	3.4313	0.5099	953.8	1 17|30	7.94	4	8
R16062_1_KIAA,7086	K.VDNALQSGNSQESVTEQDSK.D	2137.1621	1.8861	3	1	3.9621	0.5102	1709.3	1 33|76	3.92	3	6
R16062_1_KIAA,7295	K.VDNALQSGNSQESVTEQDSK.D	2137.1621	-0.2969	2	1	4.4397	0.5979	1523.1	1 23|38	3.92	3	6
R16062_1_KIAA,7298	K.VDNALQSGNSQESVTEQDSK.D	2137.1621	1.3441	2	1	2.5383	0.4696	1108.3	1 20|38	3.92	3	6

	R16062_1_KIAA,7309	K.VDNALQSGNSQESVTEQDSK.D		2137.1621	-0.7909	2	1		4.1369	0.6214	1858.9	1 22|38	3.92	3	6
	R16062_1_KIAA,7311	K.VDNALQSGNSQESVTEQDSK.D		2137.1621	0.5991	2	1		2.7977	0.4484	450.8	1 17|38	3.92	3	6
	R16062_1_KIAA,9132	K.VYACEVTHQGLSSPVTK.S		1877.0816	-0.5874	2	1		2.836	0.5254	690.8	1 15|32	6.71	3	6
	R16062_1_KIAA,9134	K.VYACEVTHQGLSSPVTK.S		1877.0816	1.3236	2	1		3.0585	0.5121	845.1	1 18|32	6.71	3	6
	R16062_1_KIAA,9143	K.VYACEVTHQGLSSPVTK.S		1877.0816	0.5556	2	1		2.9064	0.4781	923.3	1 17|32	6.71	3	6
	R16062_1_KIAA,10696	R.FSGSGSGTDFTLK.I		1304.3874	-1.4876	2	1		3.2111	0.2331	1179.5	1 17|24	5.84	1	2
	R16062_1_KIAA,10705	R.FSGSGSGTDFTLK.I		1304.3874	-1.2226	2	1		3.4803	0.2547	1142.3	1 16|24	5.84	1	2
$7-1			31	6	42.98%	25645.94	6.15	tr|V9HW34|V9HW34_HUMAN Epididymis luminal protein 213 OS=Homo sapiens GN=HEL-213 PE=2 SV=1							
$7-2			31	6	42.80%	25833.95	6.14	tr|Q6PIL8|Q6PIL8_HUMAN IGK@ protein OS=Homo sapiens GN=IGK@ PE=1 SV=1							
$7-3			31	6	42.98%	25520.68	6.14	tr|Q6PJF2|Q6PJF2_HUMAN IGK@ protein OS=Homo sapiens GN=IGK@ PE=1 SV=1							
	R16062_1_KIAA,16535	-.TVAAPSVFIFPPSDEQLK.S		1947.22	-1.248	2	1		2.5905	0.4133	292.4	1 13|34	4.37	4	8
	R16062_1_KIAA,16557	-.TVAAPSVFIFPPSDEQLK.S		1947.22	-1.181	2	1		2.2549	0.2349	229.1	1 13|34	4.37	4	8
	R16062_1_KIAA,16777	-.TVAAPSVFIFPPSDEQLK.S		1947.22	0.782	2	1		2.4042	0.3821	524.6	1 19|34	4.37	4	8
	R16062_1_KIAA,16788	-.TVAAPSVFIFPPSDEQLK.S		1947.22	0.82	2	1		2.4522	0.5211	388.3	1 16|34	4.37	4	8
	R16062_1_KIAA,17195 - 17196	-.TVAAPSVFIFPPSDEQLK.S		1947.22	0.531	2	1		2.9491	0.4563	756.2	1 21|34	4.37	4	8
	R16062_1_KIAA,17206	-.TVAAPSVFIFPPSDEQLK.S		1947.22	0.674	2	1		2.3807	0.3829	237.9	1 13|34	4.37	4	8
	R16062_1_KIAA,17485	-.TVAAPSVFIFPPSDEQLK.S		1947.22	1.737	2	1		2.6139	0.4106	390	1 16|34	4.37	4	8
	R16062_1_KIAA,13302	K.DSTYSLSSTLTLSK.A		1503.6336	0.9196	2	1		2.7028	0.4304	903.4	1 16|26	5.84	3	6
	R16062_1_KIAA,13312	K.DSTYSLSSTLTLSK.A		1503.6336	0.2906	2	1		2.6194	0.4473	718.5	1 15|26	5.84	3	6
	R16062_1_KIAA,16001	K.SGTASVVCLLNNFYPR.E		1799.0141	0.3261	2	1		2.9192	0.5415	550.6	1 13|30	7.94	4	8
	R16062_1_KIAA,16	K.SGTASVVCLLNNFYPR.E		1799.0141	0.1381	2	1		3.312	0.4871	1199.2	1 18|30	7.94	4	8

033												
R16062_1_KIAA,17303	K.SGTASVVCLLNNFYPR.E	1799.0141	-0.3619	2	1	2.4085	0.4512	580.2	1 14|30	7.94	4	8
R16062_1_KIAA,17350	K.SGTASVVCLLNNFYPR.E	1799.0141	-0.1429	2	1	2.8188	0.4831	927.2	1 15|30	7.94	4	8
R16062_1_KIAA,18804	K.SGTASVVCLLNNFYPR.E	1799.0141	1.6411	2	1	2.9725	0.5376	1033.7	1 17|30	7.94	4	8
R16062_1_KIAA,18845	K.SGTASVVCLLNNFYPR.E	1799.0141	0.5641	2	1	2.4368	0.4778	1164.4	1 18|30	7.94	4	8
R16062_1_KIAA,18876	K.SGTASVVCLLNNFYPR.E	1799.0141	2.0111	2	1	2.9342	0.4613	1195.6	1 18|30	7.94	4	8
R16062_1_KIAA,18922	K.SGTASVVCLLNNFYPR.E	1799.0141	-2.5899	2	1	3.8096	0.4571	1228.7	1 17|30	7.94	4	8
R16062_1_KIAA,19029	K.SGTASVVCLLNNFYPR.E	1799.0141	-2.6549	2	1	3.1649	0.3967	843.2	1 15|30	7.94	4	8
R16062_1_KIAA,19259	K.SGTASVVCLLNNFYPR.E	1799.0141	0.8761	2	1	2.7348	0.4147	845.6	1 15|30	7.94	4	8
R16062_1_KIAA,19266	K.SGTASVVCLLNNFYPR.E	1799.0141	-0.5639	2	1	3.5693	0.5112	830.8	1 16|30	7.94	4	8
R16062_1_KIAA,19342	K.SGTASVVCLLNNFYPR.E	1799.0141	0.4391	2	1	3.2765	0.4663	1053.3	1 17|30	7.94	4	8
R16062_1_KIAA,19358	K.SGTASVVCLLNNFYPR.E	1799.0141	0.4801	2	1	3.4313	0.5099	953.8	1 17|30	7.94	4	8
R16062_1_KIAA,7086	K.VDNALQSGNSQESVTEQDSK.D	2137.1621	1.8861	3	1	3.9621	0.5102	1709.3	1 33|76	3.92	3	6
R16062_1_KIAA,7295	K.VDNALQSGNSQESVTEQDSK.D	2137.1621	-0.2969	2	1	4.4397	0.5979	1523.1	1 23|38	3.92	3	6
R16062_1_KIAA,7298	K.VDNALQSGNSQESVTEQDSK.D	2137.1621	1.3441	2	1	2.5383	0.4696	1108.3	1 20|38	3.92	3	6
R16062_1_KIAA,7309	K.VDNALQSGNSQESVTEQDSK.D	2137.1621	-0.7909	2	1	4.1369	0.6214	1858.9	1 22|38	3.92	3	6
R16062_1_KIAA,7311	K.VDNALQSGNSQESVTEQDSK.D	2137.1621	0.5991	2	1	2.7977	0.4484	450.8	1 17|38	3.92	3	6
R16062_1_KIAA,9132	K.VYACEVTHQGLSSPVTK.S	1877.0816	-0.5874	2	1	2.836	0.5254	690.8	1 15|32	6.71	3	6
R16062_1_KIAA,9134	K.VYACEVTHQGLSSPVTK.S	1877.0816	1.3236	2	1	3.0585	0.5121	845.1	1 18|32	6.71	3	6
R16062_1_KIAA,9143	K.VYACEVTHQGLSSPVTK.S	1877.0816	0.5556	2	1	2.9064	0.4781	923.3	1 17|32	6.71	3	6
R16062_1_KIAA,13188	R.FSGSGSGTDFTLTISR.L	1633.7411	-0.1499	2	1	2.4785	0.4793	434.5	1 12|30	5.84	2	8

$8-1			31	6	40.34%	25600.71	5.75	tr|A0A087WZW8|A0A087WZW8_HUMAN Protein IGKV3-11 OS=Homo sapiens GN=IGKV3-11 PE=4 SV=1							
	R16062_1_KIAA,16535	-.TVAAPSVFIFPPSDEQLK.S		1947.22	-1.248	2	1		2.5905	0.4133	292.4	1 13|34	4.37	4	8
	R16062_1_KIAA,16557	-.TVAAPSVFIFPPSDEQLK.S		1947.22	-1.181	2	1		2.2549	0.2349	229.1	1 13|34	4.37	4	8
	R16062_1_KIAA,16777	-.TVAAPSVFIFPPSDEQLK.S		1947.22	0.782	2	1		2.4042	0.3821	524.6	1 19|34	4.37	4	8
	R16062_1_KIAA,16788	-.TVAAPSVFIFPPSDEQLK.S		1947.22	0.82	2	1		2.4522	0.5211	388.3	1 16|34	4.37	4	8
	R16062_1_KIAA,17195 - 17196	-.TVAAPSVFIFPPSDEQLK.S		1947.22	0.531	2	1		2.9491	0.4563	756.2	1 21|34	4.37	4	8
	R16062_1_KIAA,17206	-.TVAAPSVFIFPPSDEQLK.S		1947.22	0.674	2	1		2.3807	0.3829	237.9	1 13|34	4.37	4	8
	R16062_1_KIAA,17485	-.TVAAPSVFIFPPSDEQLK.S		1947.22	1.737	2	1		2.6139	0.4106	390	1 16|34	4.37	4	8
	R16062_1_KIAA,13302	K.DSTYSLSSTLTLSK.A		1503.6336	0.9196	2	1		2.7028	0.4304	903.4	1 16|26	5.84	3	6
	R16062_1_KIAA,13312	K.DSTYSLSSTLTLSK.A		1503.6336	0.2906	2	1		2.6194	0.4473	718.5	1 15|26	5.84	3	6
	R16062_1_KIAA,16001	K.SGTASVVCLLNNFYPR.E		1799.0141	0.3261	2	1		2.9192	0.5415	550.6	1 13|30	7.94	4	8
	R16062_1_KIAA,16033	K.SGTASVVCLLNNFYPR.E		1799.0141	0.1381	2	1		3.312	0.4871	1199.2	1 18|30	7.94	4	8
	R16062_1_KIAA,17303	K.SGTASVVCLLNNFYPR.E		1799.0141	-0.3619	2	1		2.4085	0.4512	580.2	1 14|30	7.94	4	8
	R16062_1_KIAA,17350	K.SGTASVVCLLNNFYPR.E		1799.0141	-0.1429	2	1		2.8188	0.4831	927.2	1 15|30	7.94	4	8
	R16062_1_KIAA,18804	K.SGTASVVCLLNNFYPR.E		1799.0141	1.6411	2	1		2.9725	0.5376	1033.7	1 17|30	7.94	4	8
	R16062_1_KIAA,18845	K.SGTASVVCLLNNFYPR.E		1799.0141	0.5641	2	1		2.4368	0.4778	1164.4	1 18|30	7.94	4	8
	R16062_1_KIAA,18876	K.SGTASVVCLLNNFYPR.E		1799.0141	2.0111	2	1		2.9342	0.4613	1195.6	1 18|30	7.94	4	8
	R16062_1_KIAA,18922	K.SGTASVVCLLNNFYPR.E		1799.0141	-2.5899	2	1		3.8096	0.4571	1228.7	1 17|30	7.94	4	8
	R16062_1_KIAA,19029	K.SGTASVVCLLNNFYPR.E		1799.0141	-2.6549	2	1		3.1649	0.3967	843.2	1 15|30	7.94	4	8
	R16062_1_KIAA,19259	K.SGTASVVCLLNNFYPR.E		1799.0141	0.8761	2	1		2.7348	0.4147	845.6	1 15|30	7.94	4	8
	R16062_1_KIAA,19	K.SGTASVVCLLNNFYPR.E		1799.0141	-0.5639	2	1		3.5693	0.5112	830.8	1 16|30	7.94	4	8

	266														
	R16062_1_KIAA,19342	K.SGTASVVCLLNNFYPR.E		1799.0141	0.4391	2	1		3.2765	0.4663	1053.3	1 17|30	7.94	4	8
	R16062_1_KIAA,19358	K.SGTASVVCLLNNFYPR.E		1799.0141	0.4801	2	1		3.4313	0.5099	953.8	1 17|30	7.94	4	8
	R16062_1_KIAA,7086	K.VDNALQSGNSQESVTEQDSK.D		2137.1621	1.8861	3	1		3.9621	0.5102	1709.3	1 33|76	3.92	3	6
	R16062_1_KIAA,7295	K.VDNALQSGNSQESVTEQDSK.D		2137.1621	-0.2969	2	1		4.4397	0.5979	1523.1	1 23|38	3.92	3	6
	R16062_1_KIAA,7298	K.VDNALQSGNSQESVTEQDSK.D		2137.1621	1.3441	2	1		2.5383	0.4696	1108.3	1 20|38	3.92	3	6
	R16062_1_KIAA,7309	K.VDNALQSGNSQESVTEQDSK.D		2137.1621	-0.7909	2	1		4.1369	0.6214	1858.9	1 22|38	3.92	3	6
	R16062_1_KIAA,7311	K.VDNALQSGNSQESVTEQDSK.D		2137.1621	0.5991	2	1		2.7977	0.4484	450.8	1 17|38	3.92	3	6
	R16062_1_KIAA,9132	K.VYACEVTHQGLSSPVTK.S		1877.0816	-0.5874	2	1		2.836	0.5254	690.8	1 15|32	6.71	3	6
	R16062_1_KIAA,9134	K.VYACEVTHQGLSSPVTK.S		1877.0816	1.3236	2	1		3.0585	0.5121	845.1	1 18|32	6.71	3	6
	R16062_1_KIAA,9143	K.VYACEVTHQGLSSPVTK.S		1877.0816	0.5556	2	1		2.9064	0.4781	923.3	1 17|32	6.71	3	6
	R16062_1_KIAA,10532	R.LLIYDASNR.A		1065.2048	1.1808	2	1		2.2253	0.3247	948.4	1 14|16	5.84	2	2
$9-1			18	6	21.75%	41286.96	8.23	sp|P01860|IGHG3_HUMAN Ig gamma-3 chain C region OS=Homo sapiens GN=IGHG3 PE=1 SV=2							
$9-2			18	6	15.86%	56889.48	8.61	tr|A0A087WXL8|A0A087WXL8_HUMAN Ig gamma-3 chain C region OS=Homo sapiens GN=IGHG3 PE=1 SV=1							
$9-3			18	6	21.75%	41215.88	8.23	tr|A0A075B6N8|A0A075B6N8_HUMAN Ig gamma-3 chain C region (Fragment) OS=Homo sapiens GN=IGHG3 PE=1 SV=1							
$9-4			18	6	15.74%	57156.04	8.46	tr|A0A087WVW2|A0A087WVW2_HUMAN Ig gamma-3 chain C region OS=Homo sapiens GN=IGHG3 PE=1 SV=1							
$9-5			18	6	15.80%	56813.18	6.43	tr|Q5EBM2|Q5EBM2_HUMAN Uncharacterized protein OS=Homo sapiens PE=1 SV=1							
$9-6			18	6	15.83%	57019.5	8.39	tr|Q6N030|Q6N030_HUMAN Putative uncharacterized protein DKFZp686I15212 OS=Homo sapiens GN=DKFZp686I15212 PE=1 SV=1							
$9-7			18	6	16.11%	56110.96	7.8	tr|Q8NF17|Q8NF17_HUMAN FLJ00385 protein (Fragment) OS=Homo sapiens GN=FLJ00385 PE=1 SV=1							
	R16062_1_KIAA,12489	K.NQVSLTCLVK.G		1162.3554	1.1304	2	1		2.3778	0.3172	753.4	1 12|18	8.22	5	34
	R16062_1_KIAA,50	K.SCDTPPPCPR.C		1187.2721	-1.7879	2	1		2.9314	0.4783	661.3	1 14|18	5.55	1	7

	96														
	R16062_1_KIAA,5348	K.SCDTPPPCPR.C		1187.2721	0.0731	2	1		2.4387	0.4028	597.3	1 13|18	5.55	1	7
	R16062_1_KIAA,5351	K.SCDTPPPCPR.C		1187.2721	2.0861	2	1		2.3361	0.5009	650.7	1 13|18	5.55	1	7
	R16062_1_KIAA,11035	K.STSGGTAALGCLVK.D		1322.484	0.516	2	1		2.6657	0.4955	841.2	1 15|26	7.94	3	30
	R16062_1_KIAA,11046	K.STSGGTAALGCLVK.D		1322.484	0.393	2	1		2.4749	0.4182	1320.9	1 19|26	7.94	3	30
	R16062_1_KIAA,5690	K.TPLGDTTHTCPR.C		1356.4605	0.2985	2	1		2.6451	0.5175	662.8	1 14|22	6.4	1	7
	R16062_1_KIAA,17394	R.CPAPELLGGPSVFLFPPKPK.D		2152.5571	1.2401	3	1		4.5673	0.53	1249.6	1 31|76	8.2	1	7
	R16062_1_KIAA,17405	R.CPAPELLGGPSVFLFPPKPK.D		2152.5571	0.7301	3	1		4.5417	0.6115	1398.2	1 34|76	8.2	1	7
	R16062_1_KIAA,17483	R.CPAPELLGGPSVFLFPPKPK.D		2152.5571	2.5651	3	1		4.389	0.6011	792.3	1 32|76	8.2	1	7
	R16062_1_KIAA,18132	R.VVSVLTVLHQDWLNGK.E		1809.1018	1.1018	2	1		2.4514	0.4444	961.7	1 16|30	6.71	3	31
	R16062_1_KIAA,18153	R.VVSVLTVLHQDWLNGK.E		1809.1018	0.7948	2	1		2.5641	0.4073	1008.1	1 17|30	6.71	3	31
	R16062_1_KIAA,18221	R.VVSVLTVLHQDWLNGK.E		1809.1018	1.9848	2	1		3.2725	0.5713	1812.9	1 21|30	6.71	3	31
	R16062_1_KIAA,18258	R.VVSVLTVLHQDWLNGK.E		1809.1018	2.1968	2	1		3.2131	0.5276	1326.1	1 19|30	6.71	3	31
	R16062_1_KIAA,18273	R.VVSVLTVLHQDWLNGK.E		1809.1018	-2.5362	2	1		4.0174	0.5921	1429.8	1 20|30	6.71	3	31
	R16062_1_KIAA,18295	R.VVSVLTVLHQDWLNGK.E		1809.1018	-2.4042	2	1		3.7172	0.4354	994.5	1 18|30	6.71	3	31
	R16062_1_KIAA,18570	R.VVSVLTVLHQDWLNGK.E		1809.1018	-1.3862	2	1		2.8304	0.5929	1186.7	1 18|30	6.71	3	31
	R16062_1_KIAA,18605	R.VVSVLTVLHQDWLNGK.E		1809.1018	-1.1742	2	1		3.1467	0.5284	1147	1 18|30	6.71	3	31
$10-1			12	6	12.48%	69366.9	5.92	sp|P02768|ALBU_HUMAN Serum albumin OS=Homo sapiens GN=ALB PE=1 SV=2							
$10-2			12	6	12.48%	69084.56	5.85	tr|Q56G89|Q56G89_HUMAN Serum albumin OS=Homo sapiens PE=2 SV=1							
$10-3			12	6	12.50%	69267.8	5.91	tr|A8K9P0|A8K9P0_HUMAN cDNA FLJ78413, highly similar to Homo sapiens albumin, mRNA OS=Homo sapiens PE=2 SV=1							
$10-4			12	6	12.30%	70363.15	5.78	tr|B4DPP6|B4DPP6_HUMAN cDNA FLJ54371, highly similar to Serum albumin OS=Homo sapiens PE=2 SV=1							

<table>
<tr><th></th><th></th><th></th><th></th><th></th><th></th><th></th><th></th><th></th><th></th><th></th><th></th><th></th><th></th><th></th><th></th></tr>
<tr><td>$10-5</td><td></td><td></td><td>12</td><td>6</td><td>12.99%</td><td>66531.48</td><td>5.73</td><td>tr|F6KPG5|F6KPG5_HUMAN Albumin (Fragment) OS=Homo sapiens PE=2 SV=1</td><td></td><td></td><td></td><td></td><td></td><td></td><td></td></tr>
<tr><td></td><td>R16062_1_KIAA,19562</td><td>K.DVFLGM*FLYEYAR.R</td><td></td><td>1640.8834</td><td>-0.2876</td><td>2</td><td>1</td><td></td><td>2.8167</td><td>0.4499</td><td>1155.5</td><td>1 17|24</td><td>4.37</td><td>1</td><td>5</td></tr>
<tr><td></td><td>R16062_1_KIAA,19588</td><td>K.DVFLGM*FLYEYAR.R</td><td></td><td>1640.8834</td><td>-0.7836</td><td>2</td><td>1</td><td></td><td>3.1188</td><td>0.4894</td><td>1171.5</td><td>1 17|24</td><td>4.37</td><td>1</td><td>5</td></tr>
<tr><td></td><td>R16062_1_KIAA,19594</td><td>K.DVFLGM*FLYEYAR.R</td><td></td><td>1640.8834</td><td>1.4894</td><td>2</td><td>1</td><td></td><td>2.6249</td><td>0.3842</td><td>1113.3</td><td>1 17|24</td><td>4.37</td><td>1</td><td>5</td></tr>
<tr><td></td><td>R16062_1_KIAA,19604</td><td>K.DVFLGM*FLYEYAR.R</td><td></td><td>1640.8834</td><td>0.3984</td><td>2</td><td>1</td><td></td><td>2.7954</td><td>0.5144</td><td>693.7</td><td>1 15|24</td><td>4.37</td><td>1</td><td>5</td></tr>
<tr><td></td><td>R16062_1_KIAA,13493</td><td>K.LVAASQAALGL.-</td><td></td><td>1014.2009</td><td>0.5919</td><td>2</td><td>1</td><td></td><td>2.3672</td><td>0.3638</td><td>925.9</td><td>1 15|20</td><td>5.53</td><td>1</td><td>5</td></tr>
<tr><td></td><td>R16062_1_KIAA,13500</td><td>K.LVAASQAALGL.-</td><td></td><td>1014.2009</td><td>0.5219</td><td>2</td><td>1</td><td></td><td>2.9673</td><td>0.4726</td><td>1505</td><td>1 16|20</td><td>5.53</td><td>1</td><td>5</td></tr>
<tr><td></td><td>R16062_1_KIAA,11158</td><td>K.LVNEVTEFAK.T</td><td></td><td>1150.3065</td><td>1.0375</td><td>2</td><td>1</td><td></td><td>2.2167</td><td>0.4809</td><td>562.3</td><td>1 13|18</td><td>4.53</td><td>1</td><td>5</td></tr>
<tr><td></td><td>R16062_1_KIAA,14569</td><td>K.QNCELFEQLGEYK.F</td><td></td><td>1658.784</td><td>0.67</td><td>2</td><td>1</td><td></td><td>2.4194</td><td>0.349</td><td>812.1</td><td>1 13|24</td><td>4.25</td><td>1</td><td>5</td></tr>
<tr><td></td><td>R16062_1_KIAA,14649</td><td>K.QNCELFEQLGEYK.F</td><td></td><td>1658.784</td><td>1.449</td><td>2</td><td>1</td><td></td><td>3.1369</td><td>0.4445</td><td>660.3</td><td>1 13|24</td><td>4.25</td><td>1</td><td>5</td></tr>
<tr><td></td><td>R16062_1_KIAA,16144</td><td>K.VFDEFKPLVEEPQNLIK.Q</td><td></td><td>2046.3515</td><td>0.6605</td><td>2</td><td>1</td><td></td><td>2.7895</td><td>0.4551</td><td>493.2</td><td>1 16|32</td><td>4.41</td><td>1</td><td>5</td></tr>
<tr><td></td><td>R16062_1_KIAA,16173</td><td>K.VFDEFKPLVEEPQNLIK.Q</td><td></td><td>2046.3515</td><td>0.6185</td><td>2</td><td>1</td><td></td><td>2.5734</td><td>0.2411</td><td>365.8</td><td>1 14|32</td><td>4.41</td><td>1</td><td>5</td></tr>
<tr><td></td><td>R16062_1_KIAA,6878</td><td>K.YICENQDSISSK.L</td><td></td><td>1444.5197</td><td>0.6307</td><td>2</td><td>1</td><td></td><td>2.5816</td><td>0.2575</td><td>624.9</td><td>1 14|22</td><td>4.37</td><td>1</td><td>5</td></tr>
<tr><td>$11-1</td><td></td><td></td><td>11</td><td>6</td><td>5.41%</td><td>226532.68</td><td>5.5</td><td>sp|P35579|MYH9_HUMAN Myosin-9 OS=Homo sapiens GN=MYH9 PE=1 SV=4</td><td></td><td></td><td></td><td></td><td></td><td></td><td></td></tr>
<tr><td>$11-2</td><td></td><td></td><td>11</td><td>6</td><td>5.41%</td><td>226532.68</td><td>5.5</td><td>tr|A0A024R1N1|A0A024R1N1_HUMAN Myosin, heavy polypeptide 9, non-muscle, isoform CRA_a OS=Homo sapiens GN=MYH9 PE=4 SV=1</td><td></td><td></td><td></td><td></td><td></td><td></td><td></td></tr>
<tr><td></td><td>R16062_1_KIAA,14607</td><td>K.ANLQIDQINTDLNLER.S</td><td></td><td>1871.042</td><td>0.459</td><td>2</td><td>1</td><td></td><td>3.8066</td><td>0.5363</td><td>1787.4</td><td>1 21|30</td><td>4.03</td><td>1</td><td>2</td></tr>
<tr><td></td><td>R16062_1_KIAA,18427</td><td>K.DFSALESQLQDTQELLQEENR.Q</td><td></td><td>2494.6107</td><td>0.4327</td><td>2</td><td>1</td><td></td><td>2.9801</td><td>0.3968</td><td>937.5</td><td>1 20|40</td><td>3.77</td><td>1</td><td>2</td></tr>
<tr><td></td><td>R16062_1_KIAA,18474</td><td>K.DFSALESQLQDTQELLQEENR.Q</td><td></td><td>2494.6107</td><td>1.1187</td><td>2</td><td>1</td><td></td><td>2.8666</td><td>0.4489</td><td>550.5</td><td>1 17|40</td><td>3.77</td><td>1</td><td>2</td></tr>
<tr><td></td><td>R16062_1_KIAA,10646</td><td>K.IAQLEEQLDNETK.E</td><td></td><td>1531.6471</td><td>0.9501</td><td>2</td><td>1</td><td></td><td>2.2806</td><td>0.4333</td><td>448.9</td><td>1 12|24</td><td>4</td><td>1</td><td>2</td></tr>
<tr><td></td><td>R16062_1_KIAA,10698</td><td>K.IAQLEEQLDNETK.E</td><td></td><td>1531.6471</td><td>0.3341</td><td>2</td><td>1</td><td></td><td>3.2559</td><td>0.4593</td><td>1307.7</td><td>1 16|24</td><td>4</td><td>1</td><td>2</td></tr>
</table>

	R16062_1_KIAA,16136	K.LQVELDNVTGLLSQSDSK.S		1947.1335	1.4715	2	1		2.616	0.4715	1127.4	1 18|34	4.03	1	2
	R16062_1_KIAA,16141	K.LQVELDNVTGLLSQSDSK.S		1947.1335	1.0245	2	1		2.7876	0.4715	546.4	1 15|34	4.03	1	2
	R16062_1_KIAA,14562	R.IAQLEEELEEEQGNTELINDR.L		2473.5898	0.9478	2	1		2.5673	0.3678	417.1	1 16|40	3.72	1	2
	R16062_1_KIAA,16114	R.TQLEELEDELQATEDAK.L		1963.0435	0.2285	2	1		3.8254	0.5727	1495.2	1 20|32	3.71	1	2
	R16062_1_KIAA,16134	R.TQLEELEDELQATEDAK.L		1963.0435	-0.3655	2	1		2.9517	0.3391	984.5	1 17|32	3.71	1	2
	R16062_1_KIAA,16142	R.TQLEELEDELQATEDAK.L		1963.0435	1.2855	2	1		3.1629	0.3846	1313.6	1 18|32	3.71	1	2
$12-1			10	6	79.21%	11022.47	6.03	tr|Q4TZM4|Q4TZM4_HUMAN Hemoglobin beta chain (Fragment) OS=Homo sapiens GN=HBB PE=3 SV=1							
	R16062_1_KIAA,11116	K.GTFATLSELHCDK.L		1479.6104	-1.6406	2	1		2.3194	0.3027	305.5	2 10|24	5.32	3	4
	R16062_1_KIAA,11123	K.GTFATLSELHCDK.L		1479.6104	0.3594	2	1		2.3893	0.4349	434.2	1 12|24	5.32	3	4
	R16062_1_KIAA,12498	K.SAVTALWGK.V		933.0864	0.8684	2	1		2.5905	0.4231	810.7	1 13|16	8.47	3	4
	R16062_1_KIAA,12521	K.SAVTALWGK.V		933.0864	0.7164	2	1		2.3857	0.3995	1095.1	1 14|16	8.47	3	4
	R16062_1_KIAA,15538	K.VLGAFSNGLAHLDNLK.G		1669.9061	1.8091	2	1		2.632	0.1932	587.5	1 16|30	6.71	1	1
	R16062_1_KIAA,10277	K.VNVDEVGGEALGR.L		1315.415	0.762	2	1		2.9846	0.1859	951.5	1 15|24	4.14	2	3
	R16062_1_KIAA,10707	K.VNVDEVGGEALGR.L		1315.415	0.086	2	1		3.2653	0.2788	1280.3	1 18|24	4.14	2	3
	R16062_1_KIAA,10734	K.VNVDEVGGEALGR.L		1315.415	1.099	2	1		2.6907	0.1598	1094.6	1 17|24	4.14	2	3
	R16062_1_KIAA,15017	R.FFESFGDLSTPDAVM*GNPK.V		2076.2717	-2.1273	2	1		2.478	0.3888	294.4	1 13|36	4.03	3	4
	R16062_1_KIAA,15697	R.LLVVYPWTQR.F		1275.5235	0.4625	2	1		2.2234	0.3052	665.8	1 11|18	8.75	4	6
$13-1			10	6	17.00%	51511.61	5.37	sp|P02679|FIBG_HUMAN Fibrinogen gamma chain OS=Homo sapiens GN=FGG PE=1 SV=3							
$13-2			10	6	16.70%	52337.57	5.37	tr|C9JC84|C9JC84_HUMAN Fibrinogen gamma chain OS=Homo sapiens GN=FGG PE=1 SV=1							
$13-3			10	6	17.30%	50322.41	5.71	tr|C9JEU5|C9JEU5_HUMAN Fibrinogen gamma chain OS=Homo sapiens GN=FGG PE=1 SV=1							
	R16062_1_KIAA,67	K.VAQLEAQCQEPCK.D		1561.7064	0.3294	2	1		2.3324	0.4243	1109.7	1 17|24	4.53	1	3

	36														
	R16062_1_KIAA,10174	K.YEASILTHDSSIR.Y		1492.6158	0.3358	2	1		2.4177	0.5063	682.7	1 13|24	5.32	1	3
	R16062_1_KIAA,9148	R.DNCCILDER.F		1195.2494	0.1004	2	1		2.7335	0.3705	977.2	1 12|16	4.03	1	3
	R16062_1_KIAA,17937	R.FGSYCPTTCGIADFLSTYQTK.V		2418.6275	-0.7605	2	1		2.3592	0.4753	298.9	1 13|40	5.82	1	3
	R16062_1_KIAA,17981	R.FGSYCPTTCGIADFLSTYQTK.V		2418.6275	0.4135	2	1		3.3801	0.6243	1035.7	1 20|40	5.82	1	3
	R16062_1_KIAA,10756	R.VELEDWNGR.T		1118.1814	0.2764	2	1		2.2222	0.3235	956.5	1 12|16	4.14	1	3
	R16062_1_KIAA,9215	R.YLQEIYNSNNQK.I		1514.6214	-1.0356	2	1		2.7723	0.2965	1595.8	1 17|22	6	1	3
	R16062_1_KIAA,9222	R.YLQEIYNSNNQK.I		1514.6214	0.2644	2	1		2.3331	0.3241	774.7	1 13|22	6	1	3
	R16062_1_KIAA,9240	R.YLQEIYNSNNQK.I		1514.6214	-0.7756	2	1		2.4073	0.3286	707.3	1 13|22	6	1	3
	R16062_1_KIAA,9258	R.YLQEIYNSNNQK.I		1514.6214	0.2874	2	1		2.6105	0.1463	503.8	1 11|22	6	1	3
$14-1			9	6	76.19%	11446.04	6.17	tr|Q6J1Z7|Q6J1Z7_HUMAN Hemoglobin beta (Fragment) OS=Homo sapiens GN=HBB PE=3 SV=1							
	R16062_1_KIAA,11116	K.GTFATLSELHCDK.L		1479.6104	-1.6406	2	1		2.3194	0.3027	305.5	2 10|24	5.32	3	4
	R16062_1_KIAA,11123	K.GTFATLSELHCDK.L		1479.6104	0.3594	2	1		2.3893	0.4349	434.2	1 12|24	5.32	3	4
	R16062_1_KIAA,12498	K.SAVTALWGK.V		933.0864	0.8684	2	1		2.5905	0.4231	810.7	1 13|16	8.47	3	4
	R16062_1_KIAA,12521	K.SAVTALWGK.V		933.0864	0.7164	2	1		2.3857	0.3995	1095.1	1 14|16	8.47	3	4
	R16062_1_KIAA,14892	K.VLGAFSDGLAHLDNLK.G		1670.8908	-0.3942	2	1		2.7099	0.1644	1055.4	1 19|30	5.21	3	5
	R16062_1_KIAA,10149	K.VNVDAVGGEALGR.L		1257.3787	0.4827	2	1		2.798	0.5541	497.2	1 14|24	4.37	2	3
	R16062_1_KIAA,10181	K.VNVDAVGGEALGR.L		1257.3787	1.1287	2	1		3.1365	0.5075	1321.3	1 17|24	4.37	2	3
	R16062_1_KIAA,15017	R.FFESFGDLSTPDAVM*GNPK.V		2076.2717	-2.1273	2	1		2.478	0.3888	294.4	1 13|36	4.03	3	4
	R16062_1_KIAA,15697	R.LLVVYPWTQR.F		1275.5235	0.4625	2	1		2.2234	0.3052	665.8	1 11|18	8.75	4	6
$15-1			9	6	2.61%	343669.73	6.26	sp|P12111|CO6A3_HUMAN Collagen alpha-3(VI) chain OS=Homo sapiens GN=COL6A3 PE=1 SV=5							

<table>
<tr><td>$15-2</td><td></td><td></td><td>9</td><td>6</td><td>3.23%</td><td>278118.62</td><td>8.42</td><td>tr|E7ENL6|E7ENL6_HUMAN Collagen alpha-3(VI) chain OS=Homo sapiens GN=COL6A3 PE=1 SV=2</td><td></td><td></td><td></td><td></td><td></td><td></td><td></td></tr>
<tr><td>$15-3</td><td></td><td></td><td>9</td><td>6</td><td>2.61%</td><td>343669.73</td><td>6.26</td><td>tr|D9ZGF2|D9ZGF2_HUMAN Collagen, type VI, alpha 3 OS=Homo sapiens GN=COL6A3 PE=4 SV=1</td><td></td><td></td><td></td><td></td><td></td><td></td><td></td></tr>
<tr><td>$15-4</td><td></td><td></td><td>9</td><td>6</td><td>3.23%</td><td>278211.77</td><td>8.4</td><td>tr|B7ZW00|B7ZW00_HUMAN COL6A3 protein OS=Homo sapiens GN=COL6A3 PE=2 SV=1</td><td></td><td></td><td></td><td></td><td></td><td></td><td></td></tr>
<tr><td></td><td>R16062_1_KIAA,14601</td><td>K.LLTPITTLTSEQIQK.L</td><td></td><td>1686.9716</td><td>0.6756</td><td>2</td><td>1</td><td></td><td>2.4492</td><td>0.334</td><td>481.4</td><td>1 13|28</td><td>6</td><td>1</td><td>4</td></tr>
<tr><td></td><td>R16062_1_KIAA,14657</td><td>K.LLTPITTLTSEQIQK.L</td><td></td><td>1686.9716</td><td>0.5346</td><td>2</td><td>1</td><td></td><td>2.4323</td><td>0.3844</td><td>593.7</td><td>1 17|28</td><td>6</td><td>1</td><td>4</td></tr>
<tr><td></td><td>R16062_1_KIAA,9095</td><td>R.IAVAQYSDDVK.V</td><td></td><td>1209.3308</td><td>0.5008</td><td>2</td><td>1</td><td></td><td>2.2047</td><td>0.4266</td><td>777</td><td>1 13|20</td><td>4.21</td><td>1</td><td>4</td></tr>
<tr><td></td><td>R16062_1_KIAA,12688</td><td>R.LQPVLQPLPSPGVGGK.R</td><td></td><td>1587.8881</td><td>1.0221</td><td>2</td><td>1</td><td></td><td>2.5104</td><td>0.4524</td><td>831.8</td><td>1 17|30</td><td>8.75</td><td>1</td><td>4</td></tr>
<tr><td></td><td>R16062_1_KIAA,12700</td><td>R.LQPVLQPLPSPGVGGK.R</td><td></td><td>1587.8881</td><td>0.8671</td><td>2</td><td>1</td><td></td><td>2.3391</td><td>0.4187</td><td>402.8</td><td>1 14|30</td><td>8.75</td><td>1</td><td>4</td></tr>
<tr><td></td><td>R16062_1_KIAA,10860</td><td>R.NNLFTSSAGYR.A</td><td></td><td>1230.3118</td><td>0.2868</td><td>2</td><td>1</td><td></td><td>2.2869</td><td>0.2323</td><td>561.8</td><td>3 11|20</td><td>8.75</td><td>1</td><td>4</td></tr>
<tr><td></td><td>R16062_1_KIAA,14431</td><td>R.QINVGNALEYVSR.N</td><td></td><td>1463.6208</td><td>-1.0942</td><td>2</td><td>1</td><td></td><td>2.2498</td><td>0.3571</td><td>444.3</td><td>1 12|24</td><td>6</td><td>1</td><td>4</td></tr>
<tr><td></td><td>R16062_1_KIAA,14501</td><td>R.QINVGNALEYVSR.N</td><td></td><td>1463.6208</td><td>0.4128</td><td>2</td><td>1</td><td></td><td>2.376</td><td>0.3774</td><td>402.1</td><td>1 12|24</td><td>6</td><td>1</td><td>4</td></tr>
<tr><td></td><td>R16062_1_KIAA,19187</td><td>R.VGVVQFSNDVFPEFYLK.T</td><td></td><td>1989.2585</td><td>0.0495</td><td>2</td><td>1</td><td></td><td>3.4892</td><td>0.5813</td><td>1004.1</td><td>1 20|32</td><td>4.37</td><td>1</td><td>4</td></tr>
<tr><td>$16-1</td><td></td><td></td><td>18</td><td>5</td><td>24.16%</td><td>35940.53</td><td>7.18</td><td>sp|P01861|IGHG4_HUMAN Ig gamma-4 chain C region OS=Homo sapiens GN=IGHG4 PE=1 SV=1</td><td></td><td></td><td></td><td></td><td></td><td></td><td></td></tr>
<tr><td>$16-2</td><td></td><td></td><td>18</td><td>5</td><td>24.16%</td><td>35940.53</td><td>7.18</td><td>tr|A0A0G2JPD4|A0A0G2JPD4_HUMAN Ig gamma-4 chain C region (Fragment) OS=Homo sapiens GN=IGHG4 PE=1 SV=2</td><td></td><td></td><td></td><td></td><td></td><td></td><td></td></tr>
<tr><td></td><td>R16062_1_KIAA,15732</td><td>K.GFYPSDIAVEWESNGQPENNYK.T</td><td></td><td>2545.6579</td><td>-0.8321</td><td>2</td><td>1</td><td></td><td>3.237</td><td>0.3584</td><td>1188.7</td><td>1 21|42</td><td>4</td><td>3</td><td>26</td></tr>
<tr><td></td><td>R16062_1_KIAA,15744</td><td>K.GFYPSDIAVEWESNGQPENNYK.T</td><td></td><td>2545.6579</td><td>0.1459</td><td>2</td><td>1</td><td></td><td>3.2157</td><td>0.376</td><td>694.7</td><td>1 19|42</td><td>4</td><td>3</td><td>26</td></tr>
<tr><td></td><td>R16062_1_KIAA,15820</td><td>K.GFYPSDIAVEWESNGQPENNYK.T</td><td></td><td>2545.6579</td><td>1.1829</td><td>2</td><td>1</td><td></td><td>4.0896</td><td>0.5161</td><td>1541.9</td><td>1 23|42</td><td>4</td><td>3</td><td>26</td></tr>
<tr><td></td><td>R16062_1_KIAA,15886</td><td>K.GFYPSDIAVEWESNGQPENNYK.T</td><td></td><td>2545.6579</td><td>-2.8691</td><td>2</td><td>1</td><td></td><td>3.3734</td><td>0.4336</td><td>1416.4</td><td>1 20|42</td><td>4</td><td>3</td><td>26</td></tr>
<tr><td></td><td>R16062_1_KIAA,15897</td><td>K.GFYPSDIAVEWESNGQPENNYK.T</td><td></td><td>2545.6579</td><td>1.2169</td><td>2</td><td>1</td><td></td><td>3.1724</td><td>0.4151</td><td>814</td><td>1 20|42</td><td>4</td><td>3</td><td>26</td></tr>
<tr><td></td><td>R16062_1_KIAA,15997</td><td>K.GFYPSDIAVEWESNGQPENNYK.T</td><td></td><td>2545.6579</td><td>-2.8691</td><td>2</td><td>1</td><td></td><td>2.9126</td><td>0.2407</td><td>646.2</td><td>1 17|42</td><td>4</td><td>3</td><td>26</td></tr>
<tr><td></td><td>R16062_1_KIAA,16</td><td>K.GFYPSDIAVEWESNGQPENNYK.</td><td></td><td>2545.6579</td><td>-1.8471</td><td>2</td><td>1</td><td></td><td>2.6677</td><td>0.46</td><td>736.1</td><td>1 20|42</td><td>4</td><td>3</td><td>26</td></tr>
</table>

	239	T														
	R16062_1_KIAA,12489	K.NQVSLTCLVK.G		1162.3554	1.1304	2	1		2.3778	0.3172	753.4	1	12|18	8.22	5	34
	R16062_1_KIAA,16471	K.TTPPVLDSDGSFFLYSR.L		1903.0809	-0.3811	2	1		2.2123	0.493	93	3	14|32	4.21	2	3
	R16062_1_KIAA,11521	R.STSESTAALGCLVK.D		1424.5731	-2.5969	2	1		3.363	0.2639	1125.9	1	19|26	5.72	4	6
	R16062_1_KIAA,18132	R.VVSVLTVLHQDWLNGK.E		1809.1018	1.1018	2	1		2.4514	0.4444	961.7	1	16|30	6.71	3	31
	R16062_1_KIAA,18153	R.VVSVLTVLHQDWLNGK.E		1809.1018	0.7948	2	1		2.5641	0.4073	1008.1	1	17|30	6.71	3	31
	R16062_1_KIAA,18221	R.VVSVLTVLHQDWLNGK.E		1809.1018	1.9848	2	1		3.2725	0.5713	1812.9	1	21|30	6.71	3	31
	R16062_1_KIAA,18258	R.VVSVLTVLHQDWLNGK.E		1809.1018	2.1968	2	1		3.2131	0.5276	1326.1	1	19|30	6.71	3	31
	R16062_1_KIAA,18273	R.VVSVLTVLHQDWLNGK.E		1809.1018	-2.5362	2	1		4.0174	0.5921	1429.8	1	20|30	6.71	3	31
	R16062_1_KIAA,18295	R.VVSVLTVLHQDWLNGK.E		1809.1018	-2.4042	2	1		3.7172	0.4354	994.5	1	18|30	6.71	3	31
	R16062_1_KIAA,18570	R.VVSVLTVLHQDWLNGK.E		1809.1018	-1.3862	2	1		2.8304	0.5929	1186.7	1	18|30	6.71	3	31
	R16062_1_KIAA,18605	R.VVSVLTVLHQDWLNGK.E		1809.1018	-1.1742	2	1		3.1467	0.5284	1147	1	18|30	6.71	3	31
$17-1			16	5	23.67%	32181.58	8.03	tr|S6BGE0|S6BGE0_HUMAN IgG H chain OS=Homo sapiens PE=2 SV=1								
	R16062_1_KIAA,11035	K.STSGGTAALGCLVK.D		1322.484	0.516	2	1		2.6657	0.4955	841.2	1	15|26	7.94	3	30
	R16062_1_KIAA,11046	K.STSGGTAALGCLVK.D		1322.484	0.393	2	1		2.4749	0.4182	1320.9	1	19|26	7.94	3	30
	R16062_1_KIAA,17998	K.THTCPPCPAPELLGGPSVFLFPPK.P		2621.0136	-0.2134	3	1		4.3027	0.4413	1646.7	1	36|92	6.41	2	23
	R16062_1_KIAA,18001	K.THTCPPCPAPELLGGPSVFLFPPK.P		2621.0136	2.4746	3	1		4.4616	0.4912	1449.2	1	36|92	6.41	2	23
	R16062_1_KIAA,18155	K.THTCPPCPAPELLGGPSVFLFPPK.P		2621.0136	2.1636	3	1		4.1152	0.5475	1058.3	1	30|92	6.41	2	23
	R16062_1_KIAA,18274	K.THTCPPCPAPELLGGPSVFLFPPK.P		2621.0136	1.4206	3	1		4.5413	0.3761	1462.8	1	36|92	6.41	2	23
	R16062_1_KIAA,16712	K.THTCPPCPAPELLGGPSVFLFPPKPK.D		2846.3022	2.8522	3	1		5.2416	0.5911	1083.5	1	39|100	7.76	2	23
	R16062_1_KIAA,16756	K.THTCPPCPAPELLGGPSVFLFPPKPK.D		2846.3022	2.3592	3	1		3.8943	0.4899	1037.1	1	35|100	7.76	2	23

	R16062_1_KIAA,16865	K.THTCPPCPAPELLGGPSVFLFPPKPK.D		2846.3022	-2.3138	3	1		4.4267	0.5224	706	1 31|100	7.76	2	23
	R16062_1_KIAA,7637	R.GTLVTVSSASTK.G		1151.2928	0.2358	2	1		2.7321	0.4169	836.4	1 17|22	8.75	2	2
	R16062_1_KIAA,12985	R.TPEVTCVVVDVSHEDPEVK.F		2140.3272	-0.5408	2	1		2.367	0.3959	560.2	1 15|36	4.17	2	23
	R16062_1_KIAA,12995	R.TPEVTCVVVDVSHEDPEVK.F		2140.3272	1.0042	2	1		2.4457	0.3006	555.8	1 15|36	4.17	2	23
	R16062_1_KIAA,13007	R.TPEVTCVVVDVSHEDPEVK.F		2140.3272	-1.3858	2	1		2.6724	0.403	851.8	1 17|36	4.17	2	23
	R16062_1_KIAA,13013	R.TPEVTCVVVDVSHEDPEVK.F		2140.3272	1.3762	2	1		2.9498	0.5184	1221.1	1 21|36	4.17	2	23
	R16062_1_KIAA,13066	R.TPEVTCVVVDVSHEDPEVK.F		2140.3272	2.6872	3	1		3.7958	0.4648	1593.5	1 34|72	4.17	2	23
	R16062_1_KIAA,13440	R.TPEVTCVVVDVSHEDPEVK.F		2140.3272	1.0192	2	1		3.0336	0.569	1086.9	1 20|36	4.17	2	23
$18-1			11	5	16.15%	37654.64	6.08	sp|P01876|IGHA1_HUMAN Ig alpha-1 chain C region OS=Homo sapiens GN=IGHA1 PE=1 SV=2							
$18-2			11	5	11.54%	53088.19	6.46	tr|Q96K68|Q96K68_HUMAN cDNA FLJ14473 fis, clone MAMMA1001080, highly similar to Homo sapiens SNC73 protein (SNC73) mRNA OS=Homo sapiens PE=2 SV=1							
$18-3			11	5	11.26%	54388.6	6.15	tr|Q6N090|Q6N090_HUMAN Putative uncharacterized protein DKFZp686G21220 (Fragment) OS=Homo sapiens GN=DKFZp686G21220 PE=2 SV=1							
$18-4			11	5	14.84%	40947.28	5.76	tr|Q9UP60|Q9UP60_HUMAN SNC73 protein OS=Homo sapiens GN=SNC73 PE=2 SV=1							
$18-5			11	5	11.26%	54459.59	6.31	tr|Q6MZW0|Q6MZW0_HUMAN Putative uncharacterized protein DKFZp686J11235 (Fragment) OS=Homo sapiens GN=DKFZp686J11235 PE=1 SV=1							
$18-6			11	5	11.56%	53224.24	6.08	tr|Q8NCL6|Q8NCL6_HUMAN cDNA FLJ90170 fis, clone MAMMA1000370, highly similar to Ig alpha-1 chain C region OS=Homo sapiens PE=2 SV=1							
	R16062_1_KIAA,4933	K.SAVQGPPER.D		941.0238	-0.0222	2	1		2.3424	0.3363	804.7	1 13|16	5.72	1	6
	R16062_1_KIAA,8252	K.TFTCTAAYPESK.T		1376.487	0.339	2	1		2.5445	0.4546	662.3	1 14|22	5.66	1	6
	R16062_1_KIAA,8263 - 8264	K.TFTCTAAYPESK.T		1376.487	0.043	2	1		3.094	0.4182	853	1 15|22	5.66	1	6
	R16062_1_KIAA,8274	K.TFTCTAAYPESK.T		1376.487	0.352	2	1		2.4841	0.5144	700.5	1 15|22	5.66	1	6
	R16062_1_KIAA,90	K.TPLTATLSK.S		932.0967	-0.9583	2	1		2.3835	0.3329	763.9	1 13|16	8.41	1	6

	13														
	R16062_1_KIAA,15132	R.QEPSQGTTTFAVTSILR.V		1837.024	0.624	2	1		2.3027	0.3858	466.5	1 16|32	6	1	6
	R16062_1_KIAA,15141	R.QEPSQGTTTFAVTSILR.V		1837.024	0.428	2	1		2.2782	0.4184	173.2	3 13|32	6	1	6
	R16062_1_KIAA,10773	R.WLQGSQELPR.E		1214.3555	-1.6745	2	1		3.2398	0.4129	1207.8	1 14|18	6	1	6
	R16062_1_KIAA,10839	R.WLQGSQELPR.E		1214.3555	-1.6205	2	1		2.2884	0.1936	964.6	1 15|18	6	1	6
	R16062_1_KIAA,10882	R.WLQGSQELPR.E		1214.3555	-0.4285	2	1		2.751	0.3897	906.3	1 14|18	6	1	6
	R16062_1_KIAA,10904	R.WLQGSQELPR.E		1214.3555	-0.5825	2	1		2.8667	0.3996	889.1	1 12|18	6	1	6
$19-1			9	5	22.09%	36053.05	8.57	sp|P04406|G3P_HUMAN Glyceraldehyde-3-phosphate dehydrogenase OS=Homo sapiens GN=GAPDH PE=1 SV=3							
$19-2			9	5	22.09%	36053.05	8.57	tr|V9HVZ4|V9HVZ4_HUMAN Glyceraldehyde-3-phosphate dehydrogenase OS=Homo sapiens GN=HEL-S-162eP PE=2 SV=1							
$19-3			9	5	22.09%	36049.05	8.57	tr|Q2TSD0|Q2TSD0_HUMAN Glyceraldehyde-3-phosphate dehydrogenase OS=Homo sapiens PE=2 SV=1							
	R16062_1_KIAA,10948	K.IISNASCTTNCLAPLAK.V		1835.0796	0.7436	2	1		2.472	0.3564	1180.8	1 18|32	8.06	1	3
	R16062_1_KIAA,11008	K.IISNASCTTNCLAPLAK.V		1835.0796	-2.2574	2	1		2.3589	0.3568	797.8	1 17|32	8.06	1	3
	R16062_1_KIAA,15409	K.LISWYDNEFGYSNR.V		1764.8749	0.5189	2	1		2.3639	0.3931	796.8	1 16|26	4.37	1	3
	R16062_1_KIAA,15580	K.LISWYDNEFGYSNR.V		1764.8749	1.6709	2	1		2.3024	0.3148	363.8	1 14|26	4.37	1	3
	R16062_1_KIAA,16615	K.LVINGNPITIFQER.D		1614.8704	0.0734	2	1		2.5766	0.4238	670.6	1 15|26	6	1	3
	R16062_1_KIAA,17037	K.LVINGNPITIFQER.D		1614.8704	-0.9176	2	1		2.3524	0.424	414.6	1 13|26	6	1	3
	R16062_1_KIAA,12074	R.GALQNIIPASTGAAK.A		1412.6169	2.8959	2	1		2.4496	0.3603	450.1	1 16|28	8.75	1	3
	R16062_1_KIAA,12279	R.VPTANVSVVDLTCR.L		1531.73	0.507	2	1		2.9187	0.5296	853.8	1 15|26	5.8	1	3
	R16062_1_KIAA,12291	R.VPTANVSVVDLTCR.L		1531.73	0.412	2	1		3.041	0.5333	823.8	1 18|26	5.8	1	3
$20-1			7	5	24.72%	30777.91	5.56	sp|P02647|APOA1_HUMAN Apolipoprotein A-I OS=Homo sapiens GN=APOA1 PE=1 SV=1							
$20-2			7	5	24.72%	30777.91	5.56	tr|A0A024R3E3|A0A024R3E3_HUMAN Apolipoprotein A-I,							

								isoform CRA_a OS=Homo sapiens GN=APOA1 PE=4 SV=1							
	R16062_1_KIAA,15634	K.LLDNWDSVTSTFSK.L		1613.7498	0.3198	2	1		2.8614	0.5166	1081	1 17|26	4.21	1	2
	R16062_1_KIAA,18887	K.VSFLSALEEYTK.K		1387.5595	1.3285	2	1		2.3388	0.4546	609.4	1 14|22	4.53	1	2
	R16062_1_KIAA,13743 - 13744	R.DYVSQFEGSALGK.Q		1401.5031	-1.5779	2	1		2.2299	0.3537	497.9	1 12|24	4.37	1	2
	R16062_1_KIAA,17562	R.EQLGPVTQEFWDNLEK.E		1934.095	-0.133	2	1		3.0549	0.5699	546	1 16|30	4	1	2
	R16062_1_KIAA,17578	R.EQLGPVTQEFWDNLEK.E		1934.095	1.783	2	1		2.2553	0.3705	282.8	1 11|30	4	1	2
	R16062_1_KIAA,17606	R.EQLGPVTQEFWDNLEK.E		1934.095	1.397	2	1		2.348	0.3938	469.1	1 13|30	4	1	2
	R16062_1_KIAA,17888	R.QGLLPVLESFK.V		1231.4658	0.4218	2	1		3.0087	0.3553	590.4	1 15|20	6	1	2
$21-1			6	5	17.73%	47650.89	5.33	sp|P01011|AACT_HUMAN Alpha-1-antichymotrypsin OS=Homo sapiens GN=SERPINA3 PE=1 SV=2							
$21-2			6	5	17.73%	47650.89	5.33	tr|A0A024R6P0|A0A024R6P0_HUMAN Serpin peptidase inhibitor, clade A (Alpha-1 antiproteinase, antitrypsin), member 3, isoform CRA_c OS=Homo sapiens GN=SERPINA3 PE=3 SV=1							
$21-3			6	5	16.74%	50628.29	5.42	tr|B3KS79|B3KS79_HUMAN cDNA FLJ35730 fis, clone TESTI2003131, highly similar to ALPHA-1-ANTICHYMOTRYPSIN OS=Homo sapiens PE=2 SV=1							
	R16062_1_KIAA,14973	K.AVLDVFEEGTEASAATAVK.I		1909.0838	0.4238	2	1		2.4444	0.3758	647.4	1 15|36	4	1	3
	R16062_1_KIAA,23457	K.DLDSQTMMVLVNYIFFK.A		2065.4418	1.0578	2	1		2.3677	0.5001	630.4	1 16|32	4.21	1	3
	R16062_1_KIAA,23458	K.DLDSQTMMVLVNYIFFK.A		2065.4418	-2.3392	2	1		2.7406	0.4321	817.5	1 16|32	4.21	1	3
	R16062_1_KIAA,17477	K.ITLLSALVETR.T		1216.4527	0.3427	2	1		2.8412	0.3932	571.4	1 12|20	6	1	3
	R16062_1_KIAA,13749	R.LYGSEAFATDFQDSAAAK.K		1892.9998	0.6538	2	1		2.3008	0.2794	691	1 16|34	4.03	1	3
	R16062_1_KIAA,7870	R.NLAVSQVVHK.A		1095.2773	-0.0577	2	1		2.5378	0.36	633.4	2 12|18	8.76	1	3
$22-1			6	5	3.10%	259210.67	5.49	tr|A0A024R462|A0A024R462_HUMAN Fibronectin 1, isoform CRA_n OS=Homo sapiens GN=FN1 PE=4 SV=1							
$22-2			6	5	3.26%	246697.8	5.72	tr|B7ZLE5|B7ZLE5_HUMAN FN1 protein OS=Homo sapiens GN=FN1 PE=2 SV=1							
	R16062_1_KIAA,12966	R.EESPLLIGQQSTVSDVPR.D		1956.1437	0.6427	2	1		2.4067	0.5093	614.8	1 18|34	4.14	1	2

	R16062_1_KIAA,13006	R.EESPLLIGQQSTVSDVPR.D		1956.1437	0.4687	2	1		2.9904	0.4207	797.7	1 19|34	4.14	1	2
	R16062_1_KIAA,16346	R.GATYNIIVEALK.D		1292.506	0.445	2	1		2.6761	0.3172	970.1	1 16|22	6	1	2
	R16062_1_KIAA,6034	R.ISCTIANR.C		935.0541	0.0671	2	1		2.2885	0.3043	754.7	1 11|14	8.25	1	2
	R16062_1_KIAA,17078	R.NTFAEVTGLSPGVTYYFK.V		1995.22	0.794	2	1		2.7369	0.4965	1317.3	1 20|34	6	1	2
	R16062_1_KIAA,19683	R.VTWAPPPSIDLTNFLVR.Y		1927.2351	-0.4959	2	1		2.2002	0.2988	194	1 16|32	5.81	1	2
$23-1			5	5	18.12%	33922.3	5.46	sp|P20774|MIME_HUMAN Mimecan OS=Homo sapiens GN=OGN PE=1 SV=1							
$23-2			5	5	18.12%	33922.3	5.46	tr|A8K0R3|A8K0R3_HUMAN Osteoglycin (Osteoinductive factor, mimecan), isoform CRA_a OS=Homo sapiens GN=OGN PE=2 SV=1							
$23-3			5	5	18.12%	33922.26	5.33	tr|Q7Z532|Q7Z532_HUMAN Osteoglycin OG OS=Homo sapiens PE=2 SV=1							
$23-4			5	5	15.17%	40553.09	8.08	tr|B4DI63|B4DI63_HUMAN cDNA FLJ59205, highly similar to Mimecan OS=Homo sapiens PE=2 SV=1							
	R16062_1_KIAA,13445	K.DFADIPNLR.R		1061.173	0.072	2	1		2.4127	0.4117	612.4	1 12|16	4.21	1	4
	R16062_1_KIAA,21658	K.LSLLEELSLAENQLLK.L		1814.1134	-0.2886	2	1		2.7343	0.365	940.8	1 18|30	4.25	1	4
	R16062_1_KIAA,20364	R.LDFTGNLIEDIEDGTFSK.L		2015.1632	0.2552	2	1		2.2627	0.3392	301.9	1 13|34	3.77	1	4
	R16062_1_KIAA,10551	R.LEGNPIVLGK.H		1040.2384	-0.9096	2	1		2.3185	0.285	839.3	1 15|18	6	1	4
	R16062_1_KIAA,19531	R.RLDFTGNLIEDIEDGTFSK.L		2171.3496	0.1756	3	1		3.9994	0.4778	1405.4	1 28|72	4.11	1	4
$24-1			12	4	14.87%	46060.9	7.63	tr|Q6N093|Q6N093_HUMAN Putative uncharacterized protein DKFZp686I04196 (Fragment) OS=Homo sapiens GN=DKFZp686I04196 PE=2 SV=1							
$24-2			12	4	13.19%	51536.19	7.51	tr|Q68CN4|Q68CN4_HUMAN Putative uncharacterized protein DKFZp686E23209 OS=Homo sapiens GN=DKFZp686E23209 PE=1 SV=2							
	R16062_1_KIAA,15732	K.GFYPSDIAVEWESNGQPENNYK.T		2545.6579	-0.8321	2	1		3.237	0.3584	1188.7	1 21|42	4	3	26
	R16062_1_KIAA,15744	K.GFYPSDIAVEWESNGQPENNYK.T		2545.6579	0.1459	2	1		3.2157	0.376	694.7	1 19|42	4	3	26
	R16062_1_KIAA,15820	K.GFYPSDIAVEWESNGQPENNYK.T		2545.6579	1.1829	2	1		4.0896	0.5161	1541.9	1 23|42	4	3	26

	R16062_1_KIAA,15886	K.GFYPSDIAVEWESNGQPENNYK.T		2545.6579	-2.8691	2	1		3.3734	0.4336	1416.4	1 20|42	4	3	26
	R16062_1_KIAA,15897	K.GFYPSDIAVEWESNGQPENNYK.T		2545.6579	1.2169	2	1		3.1724	0.4151	814	1 20|42	4	3	26
	R16062_1_KIAA,15997	K.GFYPSDIAVEWESNGQPENNYK.T		2545.6579	-2.8691	2	1		2.9126	0.2407	646.2	1 17|42	4	3	26
	R16062_1_KIAA,16239	K.GFYPSDIAVEWESNGQPENNYK.T		2545.6579	-1.8471	2	1		2.6677	0.46	736.1	1 20|42	4	3	26
	R16062_1_KIAA,12489	K.NQVSLTCLVK.G		1162.3554	1.1304	2	1		2.3778	0.3172	753.4	1 12|18	8.22	5	34
	R16062_1_KIAA,11521	R.STSESTAALGCLVK.D		1424.5731	-2.5969	2	1		3.363	0.2639	1125.9	1 19|26	5.72	4	6
	R16062_1_KIAA,16496	R.VVSVLTVVHQDWLNGK.E		1795.0751	1.2221	2	1		2.6009	0.4588	568.3	1 14|30	6.71	2	3
	R16062_1_KIAA,16926	R.VVSVLTVVHQDWLNGK.E		1795.0751	-0.3519	2	1		2.591	0.4766	567.2	1 13|30	6.71	2	3
	R16062_1_KIAA,16934	R.VVSVLTVVHQDWLNGK.E		1795.0751	0.3891	2	1		2.6677	0.44	654.6	1 14|30	6.71	2	3
$25-1			6	4	23.93%	24792.57	5.93	tr|Q8N355|Q8N355_HUMAN IGL@ protein OS=Homo sapiens GN=IGL@ PE=1 SV=1							
$25-2			6	4	23.93%	24909.77	6.19	tr|Q6GMV8|Q6GMV8_HUMAN Uncharacterized protein OS=Homo sapiens PE=2 SV=1							
	R16062_1_KIAA,4539	K.AGVETTTPSK.Q		991.0778	0.8108	2	1		2.8256	0.4209	1285.7	1 16|18	6.05	1	2
	R16062_1_KIAA,15316	K.YAASSYLSLTPEQWK.S		1744.925	-1.278	2	1		3.2108	0.4816	536.1	1 14|28	6	1	2
	R16062_1_KIAA,15328	K.YAASSYLSLTPEQWK.S		1744.925	-1.378	2	1		3.7386	0.576	535.3	1 15|28	6	1	2
	R16062_1_KIAA,9262	R.FSGSNSGNTATLTISR.V		1613.7114	0.7294	2	1		2.2685	0.4248	953.3	1 16|30	9.75	1	2
	R16062_1_KIAA,9273	R.FSGSNSGNTATLTISR.V		1613.7114	0.8554	2	1		2.8972	0.4659	976.7	1 17|30	9.75	1	2
	R16062_1_KIAA,6153	R.SYSCQVTHEGSTVEK.T		1712.7901	0.5821	2	1		2.3043	0.3286	530	1 14|28	5.38	1	2
$26-1			6	4	13.25%	36688.6	8.44	sp|P00338|LDHA_HUMAN L-lactate dehydrogenase A chain OS=Homo sapiens GN=LDHA PE=1 SV=2							
$26-2			6	4	13.25%	36688.6	8.44	tr|V9HWB9|V9HWB9_HUMAN L-lactate dehydrogenase OS=Homo sapiens GN=HEL-S-133P PE=2 SV=1							
	R16062_1_KIAA,19463	K.DLADELALVDVIEDK.L		1658.8288	0.4848	2	1		3.1949	0.4604	613.3	1 14|28	3.66	1	2
	R16062_1_KIAA,19	K.DLADELALVDVIEDK.L		1658.8288	0.8008	2	1		3.3013	0.4745	1907.5	1 21|28	3.66	1	2

	479															
	R16062_1_KIAA,16096	K.DQLIYNLLK.E		1120.3235	1.0495	2	1		2.2851	0.1518	795.4	2	12|16	5.84	1	2
	R16062_1_KIAA,16108	K.DQLIYNLLK.E		1120.3235	0.4725	2	1		2.2406	0.2249	893.8	1	13|16	5.84	1	2
	R16062_1_KIAA,12257	K.SADTLWGIQK.E		1119.2525	0.3505	2	1		2.8334	0.2829	841.5	1	13|18	5.55	1	2
	R16062_1_KIAA,7011	K.VTLTSEEEAR.L		1135.2071	0.8421	2	1		2.2665	0.3242	1022.1	1	15|18	4.25	1	2
$27-1			6	4	9.01%	53651.88	5.06	sp|P08670|VIME_HUMAN Vimentin OS=Homo sapiens GN=VIM PE=1 SV=4								
$27-2			6	4	9.74%	49653.61	5.19	tr|B0YJC4|B0YJC4_HUMAN Vimentin OS=Homo sapiens GN=VIM PE=1 SV=1								
$27-3			6	4	9.01%	53683.94	5.06	tr|Q53HU8|Q53HU8_HUMAN Vimentin variant (Fragment) OS=Homo sapiens PE=2 SV=1								
$27-4			6	4	9.01%	53651.88	5.06	tr|V9HWE1|V9HWE1_HUMAN Epididymis luminal protein 113 OS=Homo sapiens GN=HEL113 PE=2 SV=1								
$27-5			6	4	10.32%	46976.58	4.94	tr|B3KRK8|B3KRK8_HUMAN cDNA FLJ34494 fis, clone HLUNG2005030, highly similar to VIMENTIN OS=Homo sapiens PE=2 SV=1								
	R16062_1_KIAA,16131	K.ILLAELEQLK.G		1170.424	0.576	2	1		2.3539	0.288	856.9	1	14|18	4.53	1	5
	R16062_1_KIAA,16139	K.ILLAELEQLK.G		1170.424	0.057	2	1		2.922	0.2326	1329.4	1	15|18	4.53	1	5
	R16062_1_KIAA,9457	K.VELQELNDR.F		1116.207	0.125	2	1		2.6025	0.3081	1154.8	1	14|16	4.14	1	5
	R16062_1_KIAA,6031	R.QDVDNASLAR.L		1089.1416	0.3066	2	1		2.2279	0.3755	843.7	1	12|18	4.21	1	5
	R16062_1_KIAA,6041	R.QDVDNASLAR.L		1089.1416	1.0676	2	1		2.3224	0.288	653.7	1	12|18	4.21	1	5
	R16062_1_KIAA,12556	R.QVQSLTCEVDALK.G		1491.6627	0.9617	2	1		2.3639	0.2717	743.1	1	15|24	4.37	1	5
$28-1			6	4	11.97%	52420.44	7.89	tr|Q6MZX7|Q6MZX7_HUMAN Putative uncharacterized protein DKFZp686M24218 OS=Homo sapiens GN=DKFZp686M24218 PE=2 SV=1								
	R16062_1_KIAA,12489	K.NQVSLTCLVK.G		1162.3554	1.1304	2	1		2.3778	0.3172	753.4	1	12|18	8.22	5	34
	R16062_1_KIAA,16471	K.TTPPVLDSDGSFFLYSR.L		1903.0809	-0.3811	2	1		2.2123	0.493	93	3	14|32	4.21	2	3
	R16062_1_KIAA,11521	R.STSESTAALGCLVK.D		1424.5731	-2.5969	2	1		3.363	0.2639	1125.9	1	19|26	5.72	4	6

	R16062_1_KIAA,16496	R.VVSVLTVVHQDWLNGK.E		1795.0751	1.2221	2	1		2.6009	0.4588	568.3	1 14|30	6.71	2	3
	R16062_1_KIAA,16926	R.VVSVLTVVHQDWLNGK.E		1795.0751	-0.3519	2	1		2.591	0.4766	567.2	1 13|30	6.71	2	3
	R16062_1_KIAA,16934	R.VVSVLTVVHQDWLNGK.E		1795.0751	0.3891	2	1		2.6677	0.44	654.6	1 14|30	6.71	2	3
$29-1			5	4	4.34%	151076.53	9.53	sp|Q92954|PRG4_HUMAN Proteoglycan 4 OS=Homo sapiens GN=PRG4 PE=1 SV=2							
$29-2			5	4	4.34%	151076.53	9.53	tr|A0A024R930|A0A024R930_HUMAN Proteoglycan 4, isoform CRA_a OS=Homo sapiens GN=PRG4 PE=4 SV=1							
$29-3			5	4	12.35%	55494.25	9.92	tr|B3KQ20|B3KQ20_HUMAN cDNA FLJ32635 fis, clone SYNOV2000178, highly similar to Proteoglycan-4 OS=Homo sapiens PE=2 SV=1							
	R16062_1_KIAA,11060	K.DQYYNIDVPSR.T		1370.4491	0.4891	2	1		2.4448	0.2052	439.8	2 12|20	4.21	1	3
	R16062_1_KIAA,19272	K.GFGGLTGQIVAALSTAK.Y		1591.8336	-0.1364	2	1		2.6401	0.4145	1031.1	1 17|32	8.75	1	3
	R16062_1_KIAA,16847	R.GLPNVVTSAISLPNIR.K		1651.9322	0.3212	2	1		2.4166	0.4219	512.5	1 14|30	9.75	1	3
	R16062_1_KIAA,19536	R.ITEVWGIPSPIDTVFTR.C		1932.2085	1.4455	2	1		2.2221	0.4241	214.7	1 12|32	4.37	1	3
	R16062_1_KIAA,19540	R.ITEVWGIPSPIDTVFTR.C		1932.2085	0.4525	2	1		2.646	0.4173	362.3	1 17|32	4.37	1	3
$30-1			5	4	34.69%	16055.42	7.84	sp|P02042|HBD_HUMAN Hemoglobin subunit delta OS=Homo sapiens GN=HBD PE=1 SV=2							
$30-2			5	4	34.69%	16055.42	7.84	tr|A0N071|A0N071_HUMAN Delta globin OS=Homo sapiens GN=HBD PE=3 SV=1							
	R16062_1_KIAA,14892	K.VLGAFSDGLAHLDNLK.G		1670.8908	-0.3942	2	1		2.7099	0.1644	1055.4	1 19|30	5.21	3	5
	R16062_1_KIAA,10149	K.VNVDAVGGEALGR.L		1257.3787	0.4827	2	1		2.798	0.5541	497.2	1 14|24	4.37	2	3
	R16062_1_KIAA,10181	K.VNVDAVGGEALGR.L		1257.3787	1.1287	2	1		3.1365	0.5075	1321.3	1 17|24	4.37	2	3
	R16062_1_KIAA,8549	K.VVAGVANALAHK.Y		1150.3561	0.2381	2	1		2.3422	0.4164	674.9	1 13|22	8.73	2	4
	R16062_1_KIAA,15697	R.LLVVYPWTQR.F		1275.5235	0.4625	2	1		2.2234	0.3052	665.8	1 11|18	8.75	4	6
$31-1			22	3	23.37%	20314.89	6.22	tr|S6C4R6|S6C4R6_HUMAN IgG L chain OS=Homo sapiens PE=2 SV=1							
$31-2			22	3	23.37%	20372.93	5.37	tr|S6B2A1|S6B2A1_HUMAN IgG L chain OS=Homo sapiens PE=2 SV=1							

R16062_1_KIAA,16535	-.TVAAPSVFIFPPSDEQLK.S	1947.22	-1.248	2	1	2.5905	0.4133	292.4	1 13|34	4.37	4	8
R16062_1_KIAA,16557	-.TVAAPSVFIFPPSDEQLK.S	1947.22	-1.181	2	1	2.2549	0.2349	229.1	1 13|34	4.37	4	8
R16062_1_KIAA,16777	-.TVAAPSVFIFPPSDEQLK.S	1947.22	0.782	2	1	2.4042	0.3821	524.6	1 19|34	4.37	4	8
R16062_1_KIAA,16788	-.TVAAPSVFIFPPSDEQLK.S	1947.22	0.82	2	1	2.4522	0.5211	388.3	1 16|34	4.37	4	8
R16062_1_KIAA,17195 - 17196	-.TVAAPSVFIFPPSDEQLK.S	1947.22	0.531	2	1	2.9491	0.4563	756.2	1 21|34	4.37	4	8
R16062_1_KIAA,17206	-.TVAAPSVFIFPPSDEQLK.S	1947.22	0.674	2	1	2.3807	0.3829	237.9	1 13|34	4.37	4	8
R16062_1_KIAA,17485	-.TVAAPSVFIFPPSDEQLK.S	1947.22	1.737	2	1	2.6139	0.4106	390	1 16|34	4.37	4	8
R16062_1_KIAA,15188	K.LLIYWASTR.E	1123.3291	0.4591	2	1	2.2762	0.1216	807.9	2 12|16	8.75	2	3
R16062_1_KIAA,15220	K.LLIYWASTR.E	1123.3291	-0.2649	2	1	2.3459	0.3074	757.3	2 12|16	8.75	2	3
R16062_1_KIAA,16001	K.SGTASVVCLLNNFYPR.E	1799.0141	0.3261	2	1	2.9192	0.5415	550.6	1 13|30	7.94	4	8
R16062_1_KIAA,16033	K.SGTASVVCLLNNFYPR.E	1799.0141	0.1381	2	1	3.312	0.4871	1199.2	1 18|30	7.94	4	8
R16062_1_KIAA,17303	K.SGTASVVCLLNNFYPR.E	1799.0141	-0.3619	2	1	2.4085	0.4512	580.2	1 14|30	7.94	4	8
R16062_1_KIAA,17350	K.SGTASVVCLLNNFYPR.E	1799.0141	-0.1429	2	1	2.8188	0.4831	927.2	1 15|30	7.94	4	8
R16062_1_KIAA,18804	K.SGTASVVCLLNNFYPR.E	1799.0141	1.6411	2	1	2.9725	0.5376	1033.7	1 17|30	7.94	4	8
R16062_1_KIAA,18845	K.SGTASVVCLLNNFYPR.E	1799.0141	0.5641	2	1	2.4368	0.4778	1164.4	1 18|30	7.94	4	8
R16062_1_KIAA,18876	K.SGTASVVCLLNNFYPR.E	1799.0141	2.0111	2	1	2.9342	0.4613	1195.6	1 18|30	7.94	4	8
R16062_1_KIAA,18922	K.SGTASVVCLLNNFYPR.E	1799.0141	-2.5899	2	1	3.8096	0.4571	1228.7	1 17|30	7.94	4	8
R16062_1_KIAA,19029	K.SGTASVVCLLNNFYPR.E	1799.0141	-2.6549	2	1	3.1649	0.3967	843.2	1 15|30	7.94	4	8
R16062_1_KIAA,19259	K.SGTASVVCLLNNFYPR.E	1799.0141	0.8761	2	1	2.7348	0.4147	845.6	1 15|30	7.94	4	8
R16062_1_KIAA,19266	K.SGTASVVCLLNNFYPR.E	1799.0141	-0.5639	2	1	3.5693	0.5112	830.8	1 16|30	7.94	4	8
R16062_1_KIAA,19	K.SGTASVVCLLNNFYPR.E	1799.0141	0.4391	2	1	3.2765	0.4663	1053.3	1 17|30	7.94	4	8

	342														
	R16062_1_KIAA,19358	K.SGTASVVCLLNNFYPR.E		1799.0141	0.4801	2	1		3.4313	0.5099	953.8	1 17|30	7.94	4	8
$32-1			12	3	12.53%	41736.77	5.29	sp|P60709|ACTB_HUMAN Actin, cytoplasmic 1 OS=Homo sapiens GN=ACTB PE=1 SV=1							
$32-2			12	3	12.53%	41792.89	5.31	sp|P63261|ACTG_HUMAN Actin, cytoplasmic 2 OS=Homo sapiens GN=ACTG1 PE=1 SV=1							
$32-3			12	3	12.53%	41736.77	5.29	tr|Q1KLZ0|Q1KLZ0_HUMAN HCG15971, isoform CRA_a OS=Homo sapiens GN=PS1TP5BP1 PE=2 SV=1							
$32-4			12	3	18.58%	28211.41	5.2	tr|B3KWQ3|B3KWQ3_HUMAN cDNA FLJ43573 fis, clone RECTM2001691, highly similar to Actin, cytoplasmic 2 OS=Homo sapiens PE=2 SV=1							
$32-5			12	3	12.95%	40503.41	5.78	tr|Q8WVW5|Q8WVW5_HUMAN Putative uncharacterized protein (Fragment) OS=Homo sapiens PE=2 SV=1							
$32-6			12	3	15.99%	33101.04	5.43	tr|B7ZAP6|B7ZAP6_HUMAN cDNA, FLJ79260, highly similar to Actin, cytoplasmic 2 OS=Homo sapiens PE=2 SV=1							
$32-7			12	3	21.08%	25035.79	5.45	tr|V9HVZ7|V9HVZ7_HUMAN Epididymis luminal protein 176 OS=Homo sapiens GN=HEL-176 PE=2 SV=1							
$32-8			12	3	12.53%	41720.73	5.29	tr|Q53G76|Q53G76_HUMAN Beta actin variant (Fragment) OS=Homo sapiens PE=2 SV=1							
$32-9			12	3	13.39%	39225.97	5.39	tr|B4E335|B4E335_HUMAN cDNA FLJ52842, highly similar to Actin, cytoplasmic 1 OS=Homo sapiens PE=2 SV=1							
$32-10			12	3	12.53%	41764.83	5.38	tr|Q53G99|Q53G99_HUMAN Beta actin variant (Fragment) OS=Homo sapiens PE=2 SV=1							
$32-11			12	3	12.53%	41722.74	5.29	tr|Q53GK6|Q53GK6_HUMAN Beta actin variant (Fragment) OS=Homo sapiens PE=2 SV=1							
$32-12			12	3	17.87%	29411.82	5.5	tr|Q6PJ43|Q6PJ43_HUMAN ACTG1 protein (Fragment) OS=Homo sapiens GN=ACTG1 PE=2 SV=1							
$32-13			12	3	14.11%	37348.89	5.49	tr|B4DVQ0|B4DVQ0_HUMAN cDNA FLJ58286, highly similar to Actin, cytoplasmic 2 OS=Homo sapiens PE=2 SV=1							
$32-14			12	3	13.54%	38633.27	5.19	tr|B4DW52|B4DW52_HUMAN cDNA FLJ55253, highly similar to Actin, cytoplasmic 1 OS=Homo sapiens PE=2 SV=1							
$32-15			12	3	13.20%	39799.53	5.23	tr|B4E3A4|B4E3A4_HUMAN cDNA FLJ57283, highly similar to Actin, cytoplasmic 2 OS=Homo sapiens PE=2 SV=1							
	R16062_1_KIAA,14101	K.DLYANTVLSGGTTM*YPGIADR.M		2232.4564	-0.1196	2	1		2.2525	0.2719	306.9	1 13|40	4.21	1	15
	R16062_1_KIAA,14108	K.DLYANTVLSGGTTM*YPGIADR.M		2232.4564	1.5714	2	1		3.0306	0.5417	730.9	1 18|40	4.21	1	15
	R16062_1_KIAA,14115	K.DLYANTVLSGGTTM*YPGIADR.M		2232.4564	0.7294	2	1		3.2451	0.5259	1274.1	1 21|40	4.21	1	15
	R16062_1_KIAA,14	K.SYELPDGQVITIGNER.F		1791.9401	-1.4889	2	1		3.7726	0.5288	1004.6	1 18|30	4.14	1	15

	580														
	R16062_1_KIAA,14622	K.SYELPDGQVITIGNER.F		1791.9401	-1.3449	2	1		4.0839	0.568	1218	1 21|30	4.14	1	15
	R16062_1_KIAA,14786	K.SYELPDGQVITIGNER.F		1791.9401	0.7301	2	1		3.1956	0.5274	1333.5	1 21|30	4.14	1	15
	R16062_1_KIAA,14799	K.SYELPDGQVITIGNER.F		1791.9401	0.5661	2	1		2.7716	0.4033	987.7	1 17|30	4.14	1	15
	R16062_1_KIAA,8970	R.GYSFTTTAER.E		1133.1928	-1.7252	2	1		2.5308	0.483	1011.3	1 14|18	6	1	15
	R16062_1_KIAA,8979	R.GYSFTTTAER.E		1133.1928	-1.8072	2	1		2.4341	0.426	599.3	1 13|18	6	1	15
	R16062_1_KIAA,8989	R.GYSFTTTAER.E		1133.1928	0.4608	2	1		2.4407	0.272	799.1	1 15|18	6	1	15
	R16062_1_KIAA,9000	R.GYSFTTTAER.E		1133.1928	0.2688	2	1		2.2674	0.274	761.2	1 15|18	6	1	15
	R16062_1_KIAA,9014	R.GYSFTTTAER.E		1133.1928	1.7658	2	1		2.3727	0.4271	709.9	1 14|18	6	1	15
$33-1			8	3	5.25%	83264.38	4.97	sp|P08238|HS90B_HUMAN Heat shock protein HSP 90-beta OS=Homo sapiens GN=HSP90AB1 PE=1 SV=4							
$33-2			8	3	5.25%	83264.38	4.97	tr|A0A024RD80|A0A024RD80_HUMAN Heat shock protein 90kDa alpha (Cytosolic), class B member 1, isoform CRA_a OS=Homo sapiens GN=HSP90AB1 PE=3 SV=1							
$33-3			8	3	5.32%	82170.14	4.98	tr|B4DGL0|B4DGL0_HUMAN cDNA FLJ53619, highly similar to Heat shock protein HSP 90-beta OS=Homo sapiens PE=2 SV=1							
$33-4			8	3	5.54%	79194.98	4.95	tr|B4DMA2|B4DMA2_HUMAN cDNA FLJ54023, highly similar to Heat shock protein HSP 90-beta OS=Homo sapiens PE=2 SV=1							
	R16062_1_KIAA,13398	K.ADLINNLGTIAK.F		1243.435	-0.281	2	1		2.7053	0.3757	934.2	1 15|22	5.88	1	4
	R16062_1_KIAA,10131	R.ELISNASDALDK.I		1276.3755	-0.8505	2	1		2.2283	0.3378	582	2 12|22	4.03	2	10
	R16062_1_KIAA,10138	R.ELISNASDALDK.I		1276.3755	0.3215	2	1		2.204	0.3956	426.3	1 11|22	4.03	2	10
	R16062_1_KIAA,10145	R.ELISNASDALDK.I		1276.3755	-0.7615	2	1		2.6399	0.2994	692.9	1 13|22	4.03	2	10
	R16062_1_KIAA,10156	R.ELISNASDALDK.I		1276.3755	0.5355	2	1		2.5547	0.3664	861.5	1 14|22	4.03	2	10
	R16062_1_KIAA,13314	R.GVVDSEDIPLNLSR.E ! R.GVVDSEDLPLNISR.E		1514.6629	0.1039	2	1		2.2198	0.1368	391.8	1 15|26	4.03	1	4
	R16062_1_KIAA,13335	R.GVVDSEDIPLNLSR.E ! R.GVVDSEDLPLNISR.E		1514.6629	-0.0371	2	1		2.4371	0.2774	326.4	2 14|26	4.03	1	4
	R16062_1_KIAA,13	R.GVVDSEDIPLNLSR.E !		1514.6629	1.1269	2	1		2.637	0.4432	589.9	1 14|26	4.03	1	4

	356	R.GVVDSEDLPLNISR.E													
$34-1			6	3	6.08%	70374.95	5.76	sp|P34931|HS71L_HUMAN Heat shock 70 kDa protein 1-like OS=Homo sapiens GN=HSPA1L PE=1 SV=2							
$34-2			6	3	6.08%	70404.09	5.95	tr|Q53FA3|Q53FA3_HUMAN Heat shock 70 kDa protein 1-like (Fragment) OS=Homo sapiens GN=HSPA1L PE=1 SV=1							
$34-3			6	3	6.08%	70375.9	5.6	tr|B2RCQ9|B2RCQ9_HUMAN cDNA, FLJ96225, highly similar to Homo sapiens heat shock 70kDa protein 1-like (HSPA1L), mRNA OS=Homo sapiens PE=2 SV=1							
$34-4			6	3	5.53%	77561.33	8.2	tr|B4DI54|B4DI54_HUMAN cDNA FLJ56386, highly similar to Heat shock 70 kDa protein 1L OS=Homo sapiens PE=2 SV=1							
$34-5			6	3	7.33%	58332.38	6.09	tr|B4DXY3|B4DXY3_HUMAN cDNA FLJ56517, highly similar to Heat shock 70 kDa protein 1L OS=Homo sapiens PE=2 SV=1							
	R16062_1_KIAA,14993	K.DAGVIAGLNVLR.I		1198.3975	-0.0415	2	1		2.4553	0.1356	723.4	1 14|22	5.84	1	5
	R16062_1_KIAA,14463	R.IINEPTAAAIAYGLDK.R		1660.8927	0.3337	2	1		2.5215	0.2627	761.7	1 16|30	4.37	1	5
	R16062_1_KIAA,14479	R.IINEPTAAAIAYGLDK.R		1660.8927	0.4237	2	1		2.5849	0.3976	708.9	1 15|30	4.37	1	5
	R16062_1_KIAA,14545	R.IINEPTAAAIAYGLDK.R		1660.8927	1.6197	2	1		2.879	0.1941	960.8	1 17|30	4.37	1	5
	R16062_1_KIAA,7322	R.VEIIANDQGNR.I ! R.VEILANDQGNR.T		1229.3253	0.1733	2	1		2.7257	0.3648	810.8	1 15|20	4.37	1	5
	R16062_1_KIAA,7330	R.VEIIANDQGNR.I ! R.VEILANDQGNR.T		1229.3253	0.1453	2	1		2.4316	0.3158	793.9	1 13|20	4.37	1	5
$35-1			6	3	24.57%	20019.72	5.51	sp|P02792|FRIL_HUMAN Ferritin light chain OS=Homo sapiens GN=FTL PE=1 SV=2							
	R16062_1_KIAA,14207	K.LNQALLDLHALGSAR.T		1592.8248	0.6578	2	1		2.2796	0.5239	210.1	1 14|28	6.74	1	1
	R16062_1_KIAA,15900	R.DDVALEGVSHFFR.E		1492.6175	0.3775	2	1		2.6014	0.4884	1196.9	1 16|24	4.54	1	1
	R16062_1_KIAA,15913	R.DDVALEGVSHFFR.E		1492.6175	0.6375	2	1		2.6544	0.5104	1411.8	1 17|24	4.54	1	1
	R16062_1_KIAA,16428	R.LGGPEAGLGEYLFER.L		1608.7764	-1.7066	2	1		2.39	0.2371	556.7	1 13|28	4.25	1	1
	R16062_1_KIAA,16481	R.LGGPEAGLGEYLFER.L		1608.7764	-0.2406	2	1		2.7376	0.4981	1140.2	1 18|28	4.25	1	1
	R16062_1_KIAA,16579	R.LGGPEAGLGEYLFER.L		1608.7764	0.7424	2	1		2.722	0.4727	646.6	1 14|28	4.25	1	1
$36-1			4	3	5.26%	122205.24	5.44	sp|P00450|CERU_HUMAN Ceruloplasmin OS=Homo sapiens GN=CP PE=1 SV=1							
$36-2			4	3	5.26%	122205.24	5.44	tr|A5PL27|A5PL27_HUMAN CP protein OS=Homo sapiens							

								GN=CP PE=2 SV=1								
$36-3			4	3	5.57%	115471.73	5.43	tr|Q1L857|Q1L857_HUMAN Ceruloplasmin (Fragment) OS=Homo sapiens PE=4 SV=1								
	R16062_1_KIAA,17706	K.DIASGLIGPLIICK.K		1470.7729	0.6789	2	1		2.538	0.4937	749.2	1	15|26	5.83	1	3
	R16062_1_KIAA,17725	K.DIASGLIGPLIICK.K		1470.7729	0.5129	2	1		2.3486	0.2545	602.2	1	14|26	5.83	1	3
	R16062_1_KIAA,20422	K.GDSVVWYLFSAGNEADVHGIYFSGNTYLWR.G		3425.7085	1.2055	3	1		3.8353	0.5639	854.4	1	32|116	4.54	1	3
	R16062_1_KIAA,20568	K.PVWLGFLGPIIK.A		1340.6803	1.0103	2	1		2.6866	0.5734	948.9	1	15|22	9.18	1	3
$37-1			4	3	9.34%	39420.01	8.3	sp|P04075|ALDOA_HUMAN Fructose-bisphosphate aldolase A OS=Homo sapiens GN=ALDOA PE=1 SV=2								
$37-2			4	3	9.24%	39817.48	8.3	tr|J3KPS3|J3KPS3_HUMAN Fructose-bisphosphate aldolase OS=Homo sapiens GN=ALDOA PE=1 SV=1								
$37-3			4	3	9.34%	39420.01	8.3	tr|V9HWN7|V9HWN7_HUMAN Fructose-bisphosphate aldolase OS=Homo sapiens GN=HEL-S-87p PE=2 SV=1								
	R16062_1_KIAA,9748	K.GILAADESTGSIAK.R		1333.4702	0.1832	2	1		2.4034	0.3983	836.5	1	15|26	4.37	1	3
	R16062_1_KIAA,9759	K.GILAADESTGSIAK.R		1333.4702	0.0662	2	1		2.5632	0.4475	1142.1	1	17|26	4.37	1	3
	R16062_1_KIAA,7290	R.ALANSLACQGK.Y		1133.2741	0.2911	2	1		2.2183	0.3724	800.8	1	13|20	8.27	1	3
	R16062_1_KIAA,10274	R.QLLLTADDR.V		1045.1719	0.0579	2	1		2.2147	0.314	514	3	10|16	4.21	1	3
$38-1			3	3	11.71%	33296.44	9.83	sp|Q99623|PHB2_HUMAN Prohibitin-2 OS=Homo sapiens GN=PHB2 PE=1 SV=2								
$38-2			3	3	11.74%	33239.39	9.83	tr|J3KPX7|J3KPX7_HUMAN Prohibitin-2 OS=Homo sapiens GN=PHB2 PE=1 SV=2								
$38-3			3	3	13.11%	29723.39	9.87	tr|F5GY37|F5GY37_HUMAN Prohibitin-2 OS=Homo sapiens GN=PHB2 PE=1 SV=1								
	R16062_1_KIAA,12017	K.FNASQLITQR.A		1178.3231	0.1011	2	1		2.7911	0.2784	1002.3	1	13|18	9.75	1	3
	R16062_1_KIAA,5683	K.IVQAEGEAEAAK.M		1216.3233	0.4633	2	1		2.2362	0.361	808.8	1	16|22	4.25	1	3
	R16062_1_KIAA,20158	R.IPWFQYPIIYDIR.A		1725.0249	-0.3191	2	1		2.2074	0.3345	487	1	13|24	5.84	1	3
$39-1			6	2	6.11%	55928.06	8.54	sp|P02675|FIBB_HUMAN Fibrinogen beta chain OS=Homo sapiens GN=FGB PE=1 SV=2								
$39-2			6	2	8.72%	39736.44	6.95	tr|D3DP13|D3DP13_HUMAN Fibrinogen beta chain, isoform CRA_e OS=Homo sapiens GN=FGB PE=4 SV=1								

$39-3			6	2	6.11%	55928.06	8.54	tr|V9HVY1|V9HVY1_HUMAN Epididymis secretory sperm binding protein Li 78p OS=Homo sapiens GN=HEL-S-78p PE=2 SV=1								
$39-4			6	2	6.33%	54253.11	8.3	tr|B4E1D3|B4E1D3_HUMAN cDNA FLJ53952, highly similar to Fibrinogen beta chain OS=Homo sapiens PE=2 SV=1								
	R16062_1_KIAA,12455	K.DNENVVNEYSSELEK.H		1769.8018	0.7828	2	1		2.6633	0.3822	813.9	1	15|28	3.91	1	4
	R16062_1_KIAA,12470	K.DNENVVNEYSSELEK.H		1769.8018	-0.4922	2	1		2.8103	0.4837	834.6	1	15|28	3.91	1	4
	R16062_1_KIAA,12484	K.DNENVVNEYSSELEK.H		1769.8018	1.2848	2	1		2.5428	0.4215	1129.1	1	16|28	3.91	1	4
	R16062_1_KIAA,12488	K.DNENVVNEYSSELEK.H		1769.8018	1.0778	2	1		2.9904	0.4712	1535.1	1	19|28	3.91	1	4
	R16062_1_KIAA,10273	R.TPCTVSCNIPVVSGK.E		1619.829	0.649	2	1		2.8426	0.518	852.4	1	18|28	7.74	1	4
	R16062_1_KIAA,10278	R.TPCTVSCNIPVVSGK.E		1619.829	0.723	2	1		2.2383	0.3381	705.6	1	15|28	7.74	1	4
$40-1			5	2	5.27%	57936.87	7.96	sp|P14618|KPYM_HUMAN Pyruvate kinase PKM OS=Homo sapiens GN=PKM PE=1 SV=4								
$40-2			5	2	8.12%	37558	5.97	tr|A0A024R609|A0A024R609_HUMAN Pyruvate kinase OS=Homo sapiens GN=PKM2 PE=3 SV=1								
$40-3			5	2	6.13%	49897.75	7.96	tr|B4DNK4|B4DNK4_HUMAN Pyruvate kinase OS=Homo sapiens GN=PKM PE=1 SV=1								
$40-4			5	2	5.48%	55926.51	7.2	tr|B4DRT3|B4DRT3_HUMAN Pyruvate kinase OS=Homo sapiens PE=2 SV=1								
$40-5			5	2	5.27%	57936.87	7.96	tr|V9HWB8|V9HWB8_HUMAN Pyruvate kinase OS=Homo sapiens GN=HEL-S-30 PE=1 SV=1								
$40-6			5	2	5.27%	58062.05	7.61	tr|A0A024R5Z9|A0A024R5Z9_HUMAN Pyruvate kinase OS=Homo sapiens GN=PKM2 PE=3 SV=1								
$40-7			5	2	5.77%	53045.14	6.39	tr|H3BTN5|H3BTN5_HUMAN Pyruvate kinase (Fragment) OS=Homo sapiens GN=PKM PE=1 SV=1								
$40-8			5	2	9.96%	30720.17	6.46	tr|H3BQ34|H3BQ34_HUMAN Pyruvate kinase OS=Homo sapiens GN=PKM PE=1 SV=1								
	R16062_1_KIAA,13713	K.GVNLPGAAVDLPAVSEK.D		1637.8591	0.8411	2	1		2.4388	0.5428	613.3	1	17|32	4.37	1	8
	R16062_1_KIAA,13733	K.GVNLPGAAVDLPAVSEK.D		1637.8591	1.3791	2	1		2.2368	0.2775	529.4	1	15|32	4.37	1	8
	R16062_1_KIAA,11086	R.LDIDSPPITAR.N		1198.3511	-0.7019	2	1		2.283	0.1771	693.5	2	13|20	4.21	1	8
	R16062_1_KIAA,11096	R.LDIDSPPITAR.N		1198.3511	-0.7219	2	1		2.5744	0.4036	610.3	1	13|20	4.21	1	8
	R16062_1_KIAA,11	R.LDIDSPPITAR.N		1198.3511	0.3391	2	1		2.5576	0.3581	748	1	14|20	4.21	1	8

	105														
$41-1			5	2	3.49%	92469.02	4.76	sp|P14625|ENPL_HUMAN Endoplasmin OS=Homo sapiens GN=HSP90B1 PE=1 SV=1							
$41-2			5	2	8.89%	35449.69	4.62	tr|Q96GW1|Q96GW1_HUMAN Endoplasmin OS=Homo sapiens GN=HSP90B1 PE=1 SV=1							
$41-3			5	2	3.49%	92469.02	4.76	tr|V9HWP2|V9HWP2_HUMAN Epididymis luminal protein 35 OS=Homo sapiens GN=HEL-S-125m PE=2 SV=1							
$41-4			5	2	4.86%	65953.36	5.08	tr|Q59FC6|Q59FC6_HUMAN Tumor rejection antigen (Gp96) 1 variant (Fragment) OS=Homo sapiens PE=2 SV=1							
$41-5			5	2	3.49%	92339.9	4.77	tr|Q5CAQ5|Q5CAQ5_HUMAN Tumor rejection antigen (Gp96) 1 OS=Homo sapiens GN=TRA1 PE=2 SV=1							
$41-6			5	2	17.18%	18552.19	5.11	tr|B4DQX3|B4DQX3_HUMAN cDNA FLJ58927, highly similar to Endoplasmin (Heat shock protein 90 kDa beta member 1) OS=Homo sapiens PE=2 SV=1							
	R16062_1_KIAA,13405	R.EEEAIQLDGLNASQIR.E		1786.9219	0.4709	2	1		2.5173	0.2457	608	1 14|30	4	1	6
	R16062_1_KIAA,10131	R.ELISNASDALDK.I		1276.3755	-0.8505	2	1		2.2283	0.3378	582	2 12|22	4.03	2	10
	R16062_1_KIAA,10138	R.ELISNASDALDK.I		1276.3755	0.3215	2	1		2.204	0.3956	426.3	1 11|22	4.03	2	10
	R16062_1_KIAA,10145	R.ELISNASDALDK.I		1276.3755	-0.7615	2	1		2.6399	0.2994	692.9	1 13|22	4.03	2	10
	R16062_1_KIAA,10156	R.ELISNASDALDK.I		1276.3755	0.5355	2	1		2.5547	0.3664	861.5	1 14|22	4.03	2	10
$42-1			4	2	26.85%	11788.21	8.62	sp|P01598|KV106_HUMAN Ig kappa chain V-I region EU OS=Homo sapiens PE=1 SV=1							
$42-2			4	2	25.00%	12528.03	8.64	tr|A0N5G1|A0N5G1_HUMAN Rheumatoid factor C6 light chain (Fragment) OS=Homo sapiens GN=V<kappa>1 PE=2 SV=1							
	R16062_1_KIAA,9456	-.DIQM*TQSPSTLSASVGDR.V		1910.0536	-0.1154	2	1		3.0937	0.4007	831.5	1 19|34	4.21	1	2
	R16062_1_KIAA,9476	-.DIQM*TQSPSTLSASVGDR.V		1910.0536	0.1326	2	1		2.8247	0.4137	1245.5	1 21|34	4.21	1	2
	R16062_1_KIAA,9508	-.DIQM*TQSPSTLSASVGDR.V		1910.0536	1.1636	2	1		2.6679	0.3079	1031.1	1 19|34	4.21	1	2
	R16062_1_KIAA,6918	K.ASSLESGVPSR.F		1090.1695	0.2295	2	1		2.3561	0.2701	1149.3	1 15|20	6.05	1	2
$43-1			4	2	8.36%	36262.91	9.25	sp|Q13642|FHL1_HUMAN Four and a half LIM domains protein 1 OS=Homo sapiens GN=FHL1 PE=1 SV=4							
$43-2			4	2	10.51%	29160.48	8.66	tr|Q5JXI8|Q5JXI8_HUMAN Four and a half LIM domains protein 1 (Fragment) OS=Homo sapiens GN=FHL1 PE=1 SV=1							
$43-3			4	2	9.64%	31895.62	8.63	tr|Q53FI7|Q53FI7_HUMAN Four and a half LIM domains 1 variant							

								(Fragment) OS=Homo sapiens PE=2 SV=1								
$43-4			4	2	10.51%	29018.24	8.76	tr|B7Z5T3|B7Z5T3_HUMAN cDNA FLJ53644, highly similar to Four and a half LIM domains protein 1 OS=Homo sapiens PE=2 SV=1								
$43-5			4	2	10.38%	29499.96	8.67	tr|B7Z6U8|B7Z6U8_HUMAN cDNA FLJ53665, highly similar to Four and a half LIM domains protein 1 OS=Homo sapiens PE=2 SV=1								
$43-6			4	2	9.82%	31224.76	8.79	tr|B7Z5V0|B7Z5V0_HUMAN cDNA FLJ53647, highly similar to Four and a half LIM domains protein 1 OS=Homo sapiens PE=2 SV=1								
$43-7			4	2	10.89%	28097.35	8.7	tr|B7Z4B7|B7Z4B7_HUMAN cDNA FLJ52561, highly similar to Four and a half LIM domains protein 1 OS=Homo sapiens PE=2 SV=1								
	R16062_1_KIAA,7990	K.AIVAGDQNVEYK.G		1307.4344	1.2014	2	1		2.2763	0.2395	441.4	1	12|22	4.37	1	7
	R16062_1_KIAA,8021	K.AIVAGDQNVEYK.G		1307.4344	1.1744	2	1		2.3419	0.4892	962.4	1	17|22	4.37	1	7
	R16062_1_KIAA,12502	R.FTAVEDQYYCVDCYK.N		1962.0889	1.1589	2	1		2.7645	0.4774	732.7	1	16|28	4.03	1	7
	R16062_1_KIAA,12509	R.FTAVEDQYYCVDCYK.N		1962.0889	0.9699	2	1		2.9381	0.5614	1308.9	1	18|28	4.03	1	7
$44-1			4	2	6.70%	46736.49	5.37	sp|P01009|A1AT_HUMAN Alpha-1-antitrypsin OS=Homo sapiens GN=SERPINA1 PE=1 SV=3								
$44-2			4	2	6.70%	46708.44	5.37	tr|A0A024R6I7|A0A024R6I7_HUMAN Alpha-1-antitrypsin OS=Homo sapiens GN=SERPINA1 PE=1 SV=1								
$44-3			4	2	23.33%	13097.32	8.93	tr|Q9P173|Q9P173_HUMAN PRO2275 OS=Homo sapiens PE=2 SV=1								
$44-4			4	2	6.70%	46736.49	5.37	tr|E9KL23|E9KL23_HUMAN Epididymis secretory sperm binding protein Li 44a OS=Homo sapiens PE=2 SV=1								
$44-5			4	2	57.14%	4998.76	8.09	tr|B2D098|B2D098_HUMAN Alpha-1 antitrypsin Valcamonica variant (Fragment) OS=Homo sapiens PE=4 SV=1								
$44-6			4	2	7.80%	40234.75	5.26	tr|A0A0G2JRN3|A0A0G2JRN3_HUMAN Alpha-1-antitrypsin OS=Homo sapiens GN=SERPINA1 PE=1 SV=1								
	R16062_1_KIAA,13218	K.SVLGQLGITK.V		1016.2168	0.1788	2	1		2.3081	0.3967	477.6	1	12|18	8.47	1	6
	R16062_1_KIAA,12631	K.VFSNGADLSGVTEEAPLK.L		1835.0049	0.3799	2	1		3.02	0.3659	941.6	1	18|34	4.14	1	6
	R16062_1_KIAA,13142	K.VFSNGADLSGVTEEAPLK.L		1835.0049	-0.4871	2	1		2.5149	0.4886	878.1	1	18|34	4.14	1	6
	R16062_1_KIAA,13148	K.VFSNGADLSGVTEEAPLK.L		1835.0049	-0.1531	2	1		2.6291	0.3745	793.2	1	16|34	4.14	1	6

$45-1			3	2	31.48%	11992.34	5.67	sp|P01593|KV101_HUMAN Ig kappa chain V-I region AG OS=Homo sapiens PE=1 SV=1							
$45-2			3	2	31.48%	11842.07	5.11	tr|A0A087WZH9|A0A087WZH9_HUMAN Protein IGKV1-33 OS=Homo sapiens GN=IGKV1D-33 PE=4 SV=1							
	R16062_1_KIAA,9375	-.BIZM*TQSPSSLSASVGDR.V ! -.DIQM*TQSPSSLSASVGDR.V		1896.027	1.249	2	1		2.6941	0.1753	1354.9	1 21|34	5.84	1	2
	R16062_1_KIAA,14075	K.ILIYDASNLETGVPSR.F ! K.LLIYDASNLETGVPSR.F		1748.9584	-0.3256	2	1		2.8772	0.2138	791.8	1 17|30	4.37	1	2
	R16062_1_KIAA,14084	K.ILIYDASNLETGVPSR.F ! K.LLIYDASNLETGVPSR.F		1748.9584	0.5834	2	1		2.6833	0.2237	690.3	1 16|30	4.37	1	2
$46-1			3	2	23.68%	12640.09	7.92	sp|P01625|KV402_HUMAN Ig kappa chain V-IV region Len OS=Homo sapiens PE=1 SV=2							
	R16062_1_KIAA,13591	-.DIVM*TQSPDSLAVSLGER.A		1935.1462	0.5732	2	1		3.0165	0.494	1162.6	1 19|34	4.03	1	1
	R16062_1_KIAA,15188	K.LLIYWASTR.E		1123.3291	0.4591	2	1		2.2762	0.1216	807.9	2 12|16	8.75	2	3
	R16062_1_KIAA,15220	K.LLIYWASTR.E		1123.3291	-0.2649	2	1		2.3459	0.3074	757.3	2 12|16	8.75	2	3
$47-1			3	2	31.19%	11775.11	8.7	sp|P01620|KV302_HUMAN Ig kappa chain V-III region SIE OS=Homo sapiens PE=1 SV=1							
$47-2			3	2	31.19%	11788.19	8.72	sp|P01622|KV304_HUMAN Ig kappa chain V-III region Ti OS=Homo sapiens PE=1 SV=1							
$47-3			3	2	31.19%	11746.15	9.07	sp|P01623|KV305_HUMAN Ig kappa chain V-III region WOL OS=Homo sapiens PE=1 SV=1							
$47-4			3	2	31.19%	11830.35	9.34	sp|P04206|KV307_HUMAN Ig kappa chain V-III region GOL OS=Homo sapiens PE=1 SV=1							
$47-5			3	2	31.19%	11646.05	8.56	tr|Q9UL78|Q9UL78_HUMAN Myosin-reactive immunoglobulin light chain variable region (Fragment) OS=Homo sapiens PE=2 SV=1							
	R16062_1_KIAA,13308	-.EIVLTQSPGTLSLSPGER.A		1885.1086	-1.2734	2	1		3.8376	0.3449	692.7	1 19|34	4.53	1	5
	R16062_1_KIAA,13389	-.EIVLTQSPGTLSLSPGER.A		1885.1086	-0.2174	2	1		4.421	0.1852	998.1	1 22|34	4.53	1	5
	R16062_1_KIAA,13188	R.FSGSGSGTDFTLTISR.L		1633.7411	-0.1499	2	1		2.4785	0.4793	434.5	1 12|30	5.84	2	8
$48-1			3	2	8.42%	41654.09	7.16	sp|P21810|PGS1_HUMAN Biglycan OS=Homo sapiens GN=BGN PE=1 SV=2							
$48-2			3	2	8.42%	41655.04	6.6	tr|Q53HU6|Q53HU6_HUMAN Biglycan preproprotein variant (Fragment) OS=Homo sapiens PE=2 SV=1							
$48-3			3	2	9.28%	37618.39	8.08	tr|B4DDQ2|B4DDQ2_HUMAN cDNA FLJ55187, highly similar to Biglycan OS=Homo sapiens PE=2 SV=1							

$48-4			3	2	8.42%	41669.06	7.16	tr|A8K7E0|A8K7E0_HUMAN cDNA FLJ76911, highly similar to Homo sapiens biglycan (BGN), mRNA OS=Homo sapiens PE=2 SV=1								
$48-5			3	2	8.42%	41605.01	7.16	tr|Q53FI4|Q53FI4_HUMAN Biglycan preproprotein variant (Fragment) OS=Homo sapiens PE=2 SV=1								
$48-6			3	2	10.10%	34875.51	9.05	tr|A6NLG9|A6NLG9_HUMAN cDNA FLJ36740 fis, clone UTERU2013322, highly similar to Biglycan OS=Homo sapiens PE=2 SV=1								
$48-7			3	2	9.01%	39041.93	6.76	tr|B3KS75|B3KS75_HUMAN cDNA FLJ35704 fis, clone SPLEN2020183, highly similar to Biglycan OS=Homo sapiens PE=2 SV=1								
	R16062_1_KIAA,16629	K.EISPDTTLLDLQNNDISELR.K		2287.4669	0.7339	2	1		3.2138	0.5064	976.7	1	21|38	3.77	1	7
	R16062_1_KIAA,17604	K.IQAIELEDLLR.Y		1313.5253	0.7303	2	1		2.2133	0.2391	820.3	1	13|20	4.14	1	7
	R16062_1_KIAA,17615	K.IQAIELEDLLR.Y		1313.5253	0.4903	2	1		3.1041	0.272	1190	1	16|20	4.14	1	7
$49-1			3	2	3.73%	84740.22	5.93	tr|D3DTX7|D3DTX7_HUMAN Collagen, type I, alpha 1, isoform CRA_a OS=Homo sapiens GN=COL1A1 PE=4 SV=1								
	R16062_1_KIAA,5178	K.GESGPSGPAGPTGAR.G		1298.3445	0.1445	2	1		2.2134	0.4859	438.2	1	14|28	6	1	1
	R16062_1_KIAA,8421	R.GETGPAGPAGPVGPVGAR.G		1547.6978	0.1758	2	1		2.4963	0.433	486.6	1	16|34	6	1	1
	R16062_1_KIAA,8452	R.GETGPAGPAGPVGPVGAR.G		1547.6978	-0.2332	2	1		2.7027	0.3684	764.7	1	18|34	6	1	1
$50-1			3	2	8.16%	43417.18	6.61	sp|Q9BXN1|ASPN_HUMAN Asporin OS=Homo sapiens GN=ASPN PE=1 SV=2								
$50-2			3	2	12.76%	27748.79	5.36	tr|Q5TBF2|Q5TBF2_HUMAN Asporin OS=Homo sapiens GN=ASPN PE=1 SV=1								
$50-3			3	2	8.07%	43891.57	6.03	tr|Q6P528|Q6P528_HUMAN ASPN protein OS=Homo sapiens GN=ASPN PE=2 SV=1								
$50-4			3	2	12.86%	27518.61	5.61	tr|B4E2Z7|B4E2Z7_HUMAN cDNA FLJ61562, highly similar to Asporin OS=Homo sapiens PE=2 SV=1								
	R16062_1_KIAA,17415	K.GLTSLYGLILNNNK.L		1520.7554	1.1234	2	1		2.4576	0.197	358.1	10	11|26	8.59	1	4
	R16062_1_KIAA,17476	K.GLTSLYGLILNNNK.L		1520.7554	2.2274	2	1		3.1905	0.4862	1275.5	1	16|26	8.59	1	4
	R16062_1_KIAA,15226	R.LYLSHNQLSEIPLNLPK.S		1980.2963	1.0543	3	1		3.8753	0.4121	1009.4	1	29|64	6.75	1	4
$51-1			3	2	4.98%	50140.69	9.1	sp|P68104|EF1A1_HUMAN Elongation factor 1-alpha 1 OS=Homo sapiens GN=EEF1A1 PE=1 SV=1								

$51-2			3	2	4.98%	50184.85	9.15	sp|Q5VTE0|EF1A3_HUMAN Putative elongation factor 1-alpha-like 3 OS=Homo sapiens GN=EEF1A1P5 PE=5 SV=1									
$51-3			3	2	5.78%	42824.19	8.54	tr|Q16577|Q16577_HUMAN Elongation factor 1-alpha 1 OS=Homo sapiens GN=PTI-1 PE=2 SV=1									
$51-4			3	2	4.98%	50140.69	9.1	tr|Q6IPS9|Q6IPS9_HUMAN Elongation factor 1-alpha OS=Homo sapiens GN=EEF1A1 PE=2 SV=1									
$51-5			3	2	5.40%	46269.13	9.08	tr|Q9NZS6|Q9NZS6_HUMAN Elongation factor 1-alpha (Fragment) OS=Homo sapiens PE=2 SV=1									
$51-6			3	2	4.98%	50122.66	9.1	tr|Q6IPN6|Q6IPN6_HUMAN Elongation factor 1-alpha OS=Homo sapiens GN=EEF1A1 PE=2 SV=1									
$51-7			3	2	4.98%	50200.79	9.1	tr|A8K9C4|A8K9C4_HUMAN Elongation factor 1-alpha OS=Homo sapiens PE=2 SV=1									
$51-8			3	2	5.22%	47883.07	9.12	tr|A0A087WVQ9|A0A087WVQ9_HUMAN Elongation factor 1-alpha 1 OS=Homo sapiens GN=EEF1A1 PE=1 SV=1									
$51-9			3	2	4.98%	50184.73	9.14	tr|Q6IPT9|Q6IPT9_HUMAN Elongation factor 1-alpha OS=Homo sapiens GN=EEF1A1 PE=2 SV=1									
$51-10			3	2	4.98%	50112.64	9.1	tr|Q53G85|Q53G85_HUMAN Elongation factor 1-alpha (Fragment) OS=Homo sapiens PE=2 SV=1									
$51-11			3	2	5.78%	43024.46	8.94	tr|Q96RE1|Q96RE1_HUMAN Elongation factor 1-alpha OS=Homo sapiens GN=EEF1A1L14 PE=2 SV=1									
$51-12			3	2	4.98%	50110.67	9.1	tr|Q53GA1|Q53GA1_HUMAN Elongation factor 1-alpha (Fragment) OS=Homo sapiens PE=2 SV=1									
$51-13			3	2	4.98%	50110.67	9.1	tr|Q53GE9|Q53GE9_HUMAN Elongation factor 1-alpha (Fragment) OS=Homo sapiens PE=2 SV=1									
$51-14			3	2	4.98%	50124.65	9.1	tr|Q53HR5|Q53HR5_HUMAN Elongation factor 1-alpha (Fragment) OS=Homo sapiens PE=2 SV=1									
$51-15			3	2	4.98%	50198.73	9.04	tr|Q53HQ7|Q53HQ7_HUMAN Elongation factor 1-alpha (Fragment) OS=Homo sapiens PE=2 SV=1									
$51-16			3	2	5.82%	42596.1	9.12	tr|B4DNE0|B4DNE0_HUMAN cDNA FLJ52573, highly similar to Elongation factor 1-alpha 1 OS=Homo sapiens PE=2 SV=1									
$51-17			3	2	4.98%	50141.64	8.98	tr|Q53HM9|Q53HM9_HUMAN Elongation factor 1-alpha (Fragment) OS=Homo sapiens PE=2 SV=1									
	R16062_1_KIAA,9916	K.IGGIGTVPVGR.V		1026.2149	-0.8151	2	1		2.3601	0.3378	696.1	1	14|20	9.75	1	17
	R16062_1_KIAA,9939	K.IGGIGTVPVGR.V		1026.2149	0.6569	2	1		2.5936	0.4662	904	1	14|20	9.75	1	17
	R16062_1_KIAA,11618	K.YYVTIIDAPGHR.D		1405.5829	0.7269	2	1		2.2387	0.2733	660.2	1	12|22	6.74	1	17
$52-1			3	2	13.17%	22782.62	5.98	sp|P04792|HSPB1_HUMAN Heat shock protein beta-1 OS=Homo sapiens GN=HSPB1 PE=1 SV=2									
$52-2			3	2	13.17%	22782.62	5.98	tr|V9HW43|V9HW43_HUMAN Epididymis secretory protein Li 102									

								OS=Homo sapiens GN=HEL-S-102 PE=2 SV=1							
	R16062_1_KIAA,14494	K.LATQSNEITIPVTFESR.A		1907.1142	0.8632	2	1		2.4414	0.4678	307.9	1 14|32	4.53	1	2
	R16062_1_KIAA,14891	R.LFDQAFGLPR.L		1164.3381	0.3911	2	1		2.5776	0.3708	998.4	1 14|18	5.84	1	2
	R16062_1_KIAA,14900	R.LFDQAFGLPR.L		1164.3381	0.8031	2	1		2.8849	0.2327	1136.8	1 15|18	5.84	1	2
$53-1			3	2	12.78%	25565.39	9.15	sp|Q9UIJ7|KAD3_HUMAN GTP:AMP phosphotransferase AK3, mitochondrial OS=Homo sapiens GN=AK3 PE=1 SV=4							
$53-2			3	2	13.49%	24311.82	8.65	tr|Q7Z531|Q7Z531_HUMAN GTP:AMP phosphotransferase AK3, mitochondrial OS=Homo sapiens GN=AK3 PE=2 SV=1							
$53-3			3	2	12.78%	25620.47	9.3	tr|Q7Z4Y4|Q7Z4Y4_HUMAN GTP:AMP phosphotransferase AK3, mitochondrial OS=Homo sapiens GN=AK3 PE=2 SV=1							
$53-4			3	2	18.47%	18177.75	7.88	tr|B3KWD9|B3KWD9_HUMAN cDNA FLJ42834 fis, clone BRCAN2019002, highly similar to GTP:AMP phosphotransferase mitochondrial (EC 2.7.4.10) OS=Homo sapiens PE=2 SV=1							
	R16062_1_KIAA,19403	K.NLTQYSWLLDGFPR.T		1710.9135	0.6785	2	1		3.1747	0.431	1196.4	1 16|26	5.84	1	4
	R16062_1_KIAA,14383	K.TVGIDDLTGEPLIQR.E		1627.8211	0.2361	2	1		2.5787	0.3256	298.8	2 12|28	4.03	1	4
	R16062_1_KIAA,14436	K.TVGIDDLTGEPLIQR.E		1627.8211	1.4961	2	1		2.5752	0.4191	595.3	1 17|28	4.03	1	4
$54-1			3	2	5.10%	56559.96	5.26	sp|P06576|ATPB_HUMAN ATP synthase subunit beta, mitochondrial OS=Homo sapiens GN=ATP5B PE=1 SV=3							
$54-2			3	2	7.46%	38138.71	5.37	tr|H0YH81|H0YH81_HUMAN ATP synthase subunit beta (Fragment) OS=Homo sapiens GN=ATP5B PE=1 SV=1							
$54-3			3	2	10.00%	28392.5	5.85	tr|F8W0P7|F8W0P7_HUMAN ATP synthase subunit beta, mitochondrial (Fragment) OS=Homo sapiens GN=ATP5B PE=1 SV=2							
$54-4			3	2	6.07%	48113.19	4.95	tr|Q0QEN7|Q0QEN7_HUMAN ATP synthase subunit beta (Fragment) OS=Homo sapiens GN=ATP5B PE=2 SV=1							
$54-5			3	2	5.10%	56559.96	5.26	tr|V9HW31|V9HW31_HUMAN ATP synthase subunit beta OS=Homo sapiens GN=HEL-S-271 PE=1 SV=1							
	R16062_1_KIAA,14249	R.FTQAGSEVSALLGR.I		1436.5953	0.4393	2	1		3.3736	0.515	952.2	1 15|26	6	1	5
	R16062_1_KIAA,14258	R.FTQAGSEVSALLGR.I		1436.5953	0.7043	2	1		2.6827	0.5096	952.6	1 15|26	6	1	5
	R16062_1_KIAA,17352	R.VALTGLTVAEYFR.D		1440.6687	0.4877	2	1		2.7325	0.4673	664.7	1 13|24	5.97	1	5
$55-1			3	2	1.10%	280738.8	5.7	sp|P21333|FLNA_HUMAN Filamin-A OS=Homo sapiens GN=FLNA PE=1 SV=4							

$55-2			3	2	1.11%	277505.36	5.7	tr|Q60FE6|Q60FE6_HUMAN Filamin A OS=Homo sapiens GN=FLNA PE=2 SV=1							
$55-3			3	2	1.11%	278226.18	5.71	tr|Q60FE5|Q60FE5_HUMAN Filamin A OS=Homo sapiens GN=FLNA PE=1 SV=1							
$55-4			3	2	1.25%	245850.76	5.65	tr|A0A087WWY3|A0A087WWY3_HUMAN Filamin-A OS=Homo sapiens GN=FLNA PE=1 SV=1							
$55-5			3	2	1.11%	276550.17	5.69	tr|Q5HY54|Q5HY54_HUMAN Filamin-A OS=Homo sapiens GN=FLNA PE=1 SV=1							
$55-6			3	2	1.25%	245866.76	5.65	tr|A6NDY9|A6NDY9_HUMAN Filamin A OS=Homo sapiens GN=FLNA PE=2 SV=4							
	R16062_1_KIAA,10930	R.GAGTGGLGLAVEGPSEAK.M		1571.7144	0.3294	2	1		2.2376	0.3841	680.3	1 16|34	4.53	1	6
	R16062_1_KIAA,19446	R.LIALLEVLSQK.K		1227.5186	-0.1704	2	1		2.3087	0.5758	785.3	1 14|20	6	1	6
	R16062_1_KIAA,19502	R.LIALLEVLSQK.K		1227.5186	-0.8554	2	1		2.209	0.282	612.3	1 14|20	6	1	6
$56-1			2	2	22.88%	12766.4	9.15	tr|A0N5G5|A0N5G5_HUMAN Rheumatoid factor D5 light chain (Fragment) OS=Homo sapiens GN=V<kappa>3 PE=2 SV=1							
	R16062_1_KIAA,13699	-.EIVLTQSPATLSLSPGER.A		1899.1352	0.5292	2	1		2.9155	0.4558	853.7	1 17|34	4.53	1	1
	R16062_1_KIAA,10532	R.LLIYDASNR.A		1065.2048	1.1808	2	1		2.2253	0.3247	948.4	1 14|16	5.84	2	2
$57-1			2	2	11.43%	27745.18	4.73	sp|P63104|1433Z_HUMAN 14-3-3 protein zeta/delta OS=Homo sapiens GN=YWHAZ PE=1 SV=1							
$57-2			2	2	16.67%	19072.44	4.48	tr|B0AZS6|B0AZS6_HUMAN 14-3-3 protein zeta/delta OS=Homo sapiens GN=YWHAZ PE=1 SV=1							
$57-3			2	2	22.40%	13985.51	4.29	tr|B7Z2E6|B7Z2E6_HUMAN 14-3-3 protein zeta/delta OS=Homo sapiens GN=YWHAZ PE=1 SV=1							
$57-4			2	2	11.43%	27745.18	4.73	tr|D0PNI1|D0PNI1_HUMAN Epididymis luminal protein 4 OS=Homo sapiens GN=YWHAZ PE=2 SV=1							
$57-5			2	2	21.54%	14636.33	4.37	tr|H0YB80|H0YB80_HUMAN 14-3-3 protein zeta/delta (Fragment) OS=Homo sapiens GN=YWHAZ PE=1 SV=1							
$57-6			2	2	26.42%	11810.06	4.14	tr|Q2F831|Q2F831_HUMAN Tyrosine 3-monooxygenasea/tryptophan 5-monooxygenase activation protein zeta (Fragment) OS=Homo sapiens PE=2 SV=1							
	R16062_1_KIAA,17112	K.DSTLIM*QLLR.D		1206.4382	0.6502	2	1		2.2301	0.1672	887.6	1 12|18	5.84	1	6
	R16062_1_KIAA,14637	K.GIVDQSQQAYQEAFEISK.K		2042.1919	-0.5201	2	1		2.5191	0.4315	607	1 16|34	4.14	1	6
$58-1			2	2	6.40%	45205.2	6.13	sp|P00738|HPT_HUMAN Haptoglobin OS=Homo sapiens GN=HP PE=1 SV=1							

$58-2			2	2	11.40%	25457.89	6.06	tr|Q6PEJ8|Q6PEJ8_HUMAN HP protein OS=Homo sapiens GN=HP PE=2 SV=1							
$58-3			2	2	9.25%	31407.82	8.48	tr|A0A087WU08|A0A087WU08_HUMAN Haptoglobin OS=Homo sapiens GN=HP PE=1 SV=1							
$58-4			2	2	7.49%	38451.68	6.26	tr|A0A0C4DGL8|A0A0C4DGL8_HUMAN Haptoglobin OS=Homo sapiens GN=HP PE=1 SV=1							
$58-5			2	2	9.25%	31381.78	8.48	tr|Q6NSB4|Q6NSB4_HUMAN HP protein OS=Homo sapiens GN=HP PE=2 SV=1							
$58-6			2	2	11.40%	25471.92	6.24	tr|B3KP77|B3KP77_HUMAN cDNA FLJ31310 fis, clone LIVER1000165, highly similar to Haptoglobin OS=Homo sapiens PE=2 SV=1							
$58-7			2	2	5.88%	49105.84	6.17	tr|H0Y300|H0Y300_HUMAN Haptoglobin OS=Homo sapiens GN=HP PE=1 SV=4							
$58-8			2	2	7.12%	40668.96	5.54	tr|J3QLC9|J3QLC9_HUMAN Haptoglobin (Fragment) OS=Homo sapiens GN=HP PE=1 SV=1							
$58-9			2	2	6.44%	44929.88	6.13	tr|J3QR68|J3QR68_HUMAN Haptoglobin (Fragment) OS=Homo sapiens GN=HP PE=1 SV=1							
	R16062_1_KIAA,11010	K.SCAVAEYGVYVK.V		1346.504	0.169	2	1		2.3154	0.4719	556.9	1 12|22	5.72	1	9
	R16062_1_KIAA,11634	K.YVM*LPVADQDQCIR.H		1724.9537	0.5137	2	1		2.5392	0.3567	854.6	1 16|26	4.21	1	9
$59-1			2	2	2.42%	129314.11	9.08	sp|P08123|CO1A2_HUMAN Collagen alpha-2(I) chain OS=Homo sapiens GN=COL1A2 PE=1 SV=7							
$59-2			2	2	2.42%	129150.98	9.13	tr|A0A087WTA8|A0A087WTA8_HUMAN Collagen alpha-2(I) chain OS=Homo sapiens GN=COL1A2 PE=1 SV=1							
	R16062_1_KIAA,5385	R.GEAGAAGPAGPAGPR.G		1236.3195	0.5645	2	1		2.4946	0.3982	926.4	1 17|28	6	1	2
	R16062_1_KIAA,8343	R.GETGPSGPVGPAGAVGPR.G		1563.6972	0.2442	2	1		3.0171	0.398	978.3	1 19|34	6	1	2
$60-1			2	2	10.83%	25599.64	8.83	tr|S6BGF5|S6BGF5_HUMAN IgG H chain OS=Homo sapiens PE=2 SV=1							
	R16062_1_KIAA,7637	R.GTLVTVSSASTK.G		1151.2928	0.2358	2	1		2.7321	0.4169	836.4	1 17|22	8.75	2	2
	R16062_1_KIAA,11521	R.STSESTAALGCLVK.D		1424.5731	-2.5969	2	1		3.363	0.2639	1125.9	1 19|26	5.72	4	6
$61-1			4	1	13.33%	13282.83	8.62	tr|A2NYQ9|A2NYQ9_HUMAN Anti-folate binding protein (Fragment) OS=Homo sapiens GN=HuVH8B VH PE=2 SV=1							
	R16062_1_KIAA,11454	-.QVQLVESGGGLVQPGR.S		1624.8237	-0.2783	2	1		2.9155	0.4606	1459.8	1 19|30	6	1	1
	R16062_1_KIAA,11466	-.QVQLVESGGGLVQPGR.S		1624.8237	-0.6543	2	1		3.1665	0.429	1225.3	1 20|30	6	1	1

	R16062_1_KIAA,11511	-.QVQLVESGGGLVQPGR.S		1624.8237	0.3777	2	1		3.7845	0.4187	1810.9	1 21|30	6	1	1
	R16062_1_KIAA,11539	-.QVQLVESGGGLVQPGR.S		1624.8237	0.4107	2	1		3.1884	0.4102	1354.6	1 18|30	6	1	1
$62-1			4	1	7.35%	22876.37	9.73	sp|P46782|RS5_HUMAN 40S ribosomal protein S5 OS=Homo sapiens GN=RPS5 PE=1 SV=4							
$62-2			4	1	7.35%	22876.37	9.73	tr|A0A024R4Q8|A0A024R4Q8_HUMAN Ribosomal protein S5, isoform CRA_a OS=Homo sapiens GN=RPS5 PE=3 SV=1							
$62-3			4	1	6.67%	25333.23	9.78	tr|M0R0R2|M0R0R2_HUMAN 40S ribosomal protein S5 OS=Homo sapiens GN=RPS5 PE=1 SV=1							
$62-4			4	1	7.50%	22390.83	9.59	tr|M0R0F0|M0R0F0_HUMAN 40S ribosomal protein S5 (Fragment) OS=Homo sapiens GN=RPS5 PE=1 SV=1							
$62-5			4	1	11.19%	14762.75	8.66	tr|M0QZN2|M0QZN2_HUMAN 40S ribosomal protein S5 OS=Homo sapiens GN=RPS5 PE=1 SV=1							
$62-6			4	1	7.35%	22964.49	9.76	tr|Q53G25|Q53G25_HUMAN Ribosomal protein S5 variant (Fragment) OS=Homo sapiens PE=2 SV=1							
	R16062_1_KIAA,19311	K.TIAECLADELINAAK.G		1632.8311	0.1901	2	1		3.0252	0.5113	1303.4	1 18|28	4.14	1	6
	R16062_1_KIAA,19324	K.TIAECLADELINAAK.G		1632.8311	-0.3689	2	1		3.2939	0.4453	974.5	1 18|28	4.14	1	6
	R16062_1_KIAA,19340	K.TIAECLADELINAAK.G		1632.8311	1.3791	2	1		2.7191	0.2968	1609.2	1 18|28	4.14	1	6
	R16062_1_KIAA,19356	K.TIAECLADELINAAK.G		1632.8311	1.4571	2	1		3.2179	0.2962	1479.9	1 19|28	4.14	1	6
$63-1			4	1	5.53%	22110.34	8.27	sp|Q06830|PRDX1_HUMAN Peroxiredoxin-1 OS=Homo sapiens GN=PRDX1 PE=1 SV=1							
$63-2			4	1	5.56%	21891.93	5.66	sp|P32119|PRDX2_HUMAN Peroxiredoxin-2 OS=Homo sapiens GN=PRDX2 PE=1 SV=5							
$63-3			4	1	6.43%	18975.75	6.42	tr|A0A0A0MSI0|A0A0A0MSI0_HUMAN Peroxiredoxin-1 (Fragment) OS=Homo sapiens GN=PRDX1 PE=1 SV=1							
$63-4			4	1	5.56%	21891.93	5.66	tr|V9HW12|V9HW12_HUMAN Epididymis secretory sperm binding protein Li 2a OS=Homo sapiens GN=HEL-S-2a PE=2 SV=1							
$63-5			4	1	6.01%	20107.19	8.9	tr|B4DF70|B4DF70_HUMAN cDNA FLJ60461, highly similar to Peroxiredoxin-2 (EC 1.11.1.15) OS=Homo sapiens PE=2 SV=1							
$63-6			4	1	5.53%	22200.51	8.58	tr|B2R4P2|B2R4P2_HUMAN cDNA, FLJ92164, highly similar to Homo sapiens peroxiredoxin 1 (PRDX1), mRNA OS=Homo sapiens PE=2 SV=1							
$63-7			4	1	11.34%	10676.1	8.79	tr|A0A0A0MRQ5|A0A0A0MRQ5_HUMAN Peroxiredoxin-1 OS=Homo sapiens GN=PRDX1 PE=1 SV=1							
	R16062_1_KIAA,11	R.QITVNDLPVGR.S		1212.381	0.168	2	1		2.3237	0.3005	616.9	1 11|20	5.84	1	7

	432															
	R16062_1_KIAA,11472	R.QITVNDLPVGR.S		1212.381	-0.485	2	1		2.7165	0.3418	1220.6	1	16|20	5.84	1	7
	R16062_1_KIAA,11524	R.QITVNDLPVGR.S		1212.381	0.985	2	1		2.8559	0.3889	826.2	1	15|20	5.84	1	7
	R16062_1_KIAA,11533	R.QITVNDLPVGR.S		1212.381	0.515	2	1		2.6238	0.372	753.5	1	13|20	5.84	1	7
$64-1			3	1	13.22%	13437.2	9.43	sp|P01771|HV310_HUMAN Ig heavy chain V-III region HIL OS=Homo sapiens PE=1 SV=1								
	R16062_1_KIAA,12785	-.QVKLVQAGGGVVQPGR.S		1593.856	-0.632	2	1		2.2298	0.2525	394.3	3	13|30	11	1	1
	R16062_1_KIAA,12817	-.QVKLVQAGGGVVQPGR.S		1593.856	-0.418	2	1		2.8817	0.217	699.5	3	15|30	11	1	1
	R16062_1_KIAA,12861	-.QVKLVQAGGGVVQPGR.S		1593.856	-0.424	2	1		2.9304	0.2442	596.9	3	15|30	11	1	1
$65-1			3	1	7.77%	11367.28	11.36	sp|P62805|H4_HUMAN Histone H4 OS=Homo sapiens GN=HIST1H4A PE=1 SV=2								
$65-2			3	1	18.60%	4915.8	10.92	tr|Q6B823|Q6B823_HUMAN Histone H4 (Fragment) OS=Homo sapiens PE=3 SV=1								
$65-3			3	1	7.77%	11367.28	11.36	tr|B2R4R0|B2R4R0_HUMAN Histone H4 OS=Homo sapiens GN=HIST1H4L PE=2 SV=1								
$65-4			3	1	7.77%	11314.23	11.04	tr|Q0VAS5|Q0VAS5_HUMAN Histone H4 OS=Homo sapiens GN=HIST1H4H PE=2 SV=1								
	R16062_1_KIAA,13655	K.VFLENVIR.D		990.1811	0.4381	2	1		2.2759	0.2887	634.7	2	12|14	5.97	1	4
	R16062_1_KIAA,13672 - 13673	K.VFLENVIR.D		990.1811	-0.0549	2	1		2.5114	0.3316	598.7	6	12|14	5.97	1	4
	R16062_1_KIAA,13686	K.VFLENVIR.D		990.1811	0.3571	2	1		2.3892	0.3359	659.5	2	12|14	5.97	1	4
$66-1			2	1	10.00%	13352.93	5.72	tr|A0A068LKQ0|A0A068LKQ0_HUMAN Ig heavy chain variable region (Fragment) OS=Homo sapiens PE=4 SV=1								
	R16062_1_KIAA,8725	-.EVQLVQSGAEVK.K		1287.4447	-0.0633	2	1		2.8872	0.2047	648.4	1	14|22	4.53	1	1
	R16062_1_KIAA,8883	-.EVQLVQSGAEVK.K		1287.4447	0.9987	2	1		2.38	0.1118	917.3	1	15|22	4.53	1	1
$67-1			2	1	13.91%	12626.09	8.93	tr|A2J1N6|A2J1N6_HUMAN Rheumatoid factor RF-ET9 (Fragment) OS=Homo sapiens PE=2 SV=1								
	R16062_1_KIAA,10454	-.LVESGGGLVKPGGSLR.L		1526.7632	0.4632	2	1		2.3532	0.2813	688.4	1	16|30	8.75	1	1
	R16062_1_KIAA,10467	-.LVESGGGLVKPGGSLR.L		1526.7632	0.2432	2	1		2.4754	0.267	803.8	1	17|30	8.75	1	1

$68-1			2	1	14.07%	14538.35	8.93	tr|A2N2G5|A2N2G5_HUMAN VH87-2 protein (Fragment) OS=Homo sapiens GN=VH87-2 PE=2 SV=1							
	R16062_1_KIAA,14008	-.QVQLLESGGGLVKPGGSLR.L		1896.181	-0.262	2	1		3.0063	0.1654	1219.8	1 19|36	8.75	1	1
	R16062_1_KIAA,19025	-.QVQLLESGGGLVKPGGSLR.L		1896.181	-0.253	2	1		2.304	0.2297	517.6	2 13|36	8.75	1	1
$69-1			2	1	11.90%	13950.15	10.31	sp|P33778|H2B1B_HUMAN Histone H2B type 1-B OS=Homo sapiens GN=HIST1H2BB PE=1 SV=2							
$69-2			2	1	11.90%	13906.09	10.31	sp|P62807|H2B1C_HUMAN Histone H2B type 1-C/E/F/G/I OS=Homo sapiens GN=HIST1H2BC PE=1 SV=4							
$69-3			2	1	11.90%	13936.11	10.31	sp|P58876|H2B1D_HUMAN Histone H2B type 1-D OS=Homo sapiens GN=HIST1H2BD PE=1 SV=2							
$69-4			2	1	11.90%	13892.06	10.31	sp|Q93079|H2B1H_HUMAN Histone H2B type 1-H OS=Homo sapiens GN=HIST1H2BH PE=1 SV=3							
$69-5			2	1	11.90%	13904.12	10.31	sp|P06899|H2B1J_HUMAN Histone H2B type 1-J OS=Homo sapiens GN=HIST1H2BJ PE=1 SV=3							
$69-6			2	1	11.90%	13890.09	10.31	sp|O60814|H2B1K_HUMAN Histone H2B type 1-K OS=Homo sapiens GN=HIST1H2BK PE=1 SV=3							
$69-7			2	1	11.90%	13952.16	10.31	sp|Q99880|H2B1L_HUMAN Histone H2B type 1-L OS=Homo sapiens GN=HIST1H2BL PE=1 SV=3							
$69-8			2	1	11.90%	13989.22	10.31	sp|Q99879|H2B1M_HUMAN Histone H2B type 1-M OS=Homo sapiens GN=HIST1H2BM PE=1 SV=3							
$69-9			2	1	11.90%	13922.09	10.31	sp|Q99877|H2B1N_HUMAN Histone H2B type 1-N OS=Homo sapiens GN=HIST1H2BN PE=1 SV=3							
$69-10			2	1	11.90%	13906.09	10.31	sp|P23527|H2B1O_HUMAN Histone H2B type 1-O OS=Homo sapiens GN=HIST1H2BO PE=1 SV=3							
$69-11			2	1	11.90%	13920.12	10.31	sp|Q16778|H2B2E_HUMAN Histone H2B type 2-E OS=Homo sapiens GN=HIST2H2BE PE=1 SV=3							
$69-12			2	1	11.90%	13920.11	10.31	sp|Q5QNW6|H2B2F_HUMAN Histone H2B type 2-F OS=Homo sapiens GN=HIST2H2BF PE=1 SV=3							
$69-13			2	1	11.90%	13908.06	10.31	sp|Q8N257|H2B3B_HUMAN Histone H2B type 3-B OS=Homo sapiens GN=HIST3H2BB PE=1 SV=3							
$69-14			2	1	11.90%	13944.15	10.37	sp|P57053|H2BFS_HUMAN Histone H2B type F-S OS=Homo sapiens GN=H2BFS PE=1 SV=2							
$69-15			2	1	11.90%	13906.09	10.31	tr|B2R4S9|B2R4S9_HUMAN Histone H2B OS=Homo sapiens GN=HIST1H2BE PE=2 SV=1							
$69-16			2	1	11.90%	13922.09	10.31	tr|A0A024RCJ9|A0A024RCJ9_HUMAN Histone H2B OS=Homo sapiens PE=3 SV=1							
$69-17			2	1	9.04%	18803.85	10.53	tr|U3KQK0|U3KQK0_HUMAN Histone H2B OS=Homo sapiens GN=HIST1H2BN PE=1 SV=1							
$69-1			2	1	11.90%	13936.11	10.31	tr|A0A024QZZ7|A0A024QZZ7_HUMAN Histone H2B OS=Homo							

8								sapiens GN=HIST1H2BD PE=3 SV=1							
$69-19			2	1	11.90%	13904.12	10.31	tr|A0A024RCJ2|A0A024RCJ2_HUMAN Histone H2B OS=Homo sapiens GN=HIST1H2BJ PE=3 SV=1							
$69-20			2	1	10.87%	15156.5	10.25	tr|I6L9F7|I6L9F7_HUMAN Histone H2B (Fragment) OS=Homo sapiens GN=HIST1H2BM PE=2 SV=1							
$69-21			2	1	11.90%	13996.21	10.31	tr|A8K9J7|A8K9J7_HUMAN Histone H2B OS=Homo sapiens PE=2 SV=1							
$69-22			2	1	11.90%	13890.09	10.31	tr|A0A024RCL8|A0A024RCL8_HUMAN Histone H2B OS=Homo sapiens GN=HIST1H2BK PE=3 SV=1							
$69-23			2	1	9.04%	18040.84	10.31	tr|B4DR52|B4DR52_HUMAN Histone H2B OS=Homo sapiens PE=2 SV=1							
$69-24			2	1	11.90%	13834.02	10.39	tr|Q0D2M2|Q0D2M2_HUMAN HIST1H2BC protein OS=Homo sapiens GN=HIST1H2BC PE=2 SV=1							
	R16062_1_KIAA,16626	K.AM*GIM*NSFVNDIFER.I		1777.0155	0.5865	2	1		2.8943	0.4924	682.6	1 15|28	4.37	1	24
	R16062_1_KIAA,16646 - 16647	K.AM*GIM*NSFVNDIFER.I		1777.0155	1.1755	2	1		2.7722	0.5061	1088.8	1 18|28	4.37	1	24
$70-1			2	1	6.15%	29246.01	6.87	sp|P00918|CAH2_HUMAN Carbonic anhydrase 2 OS=Homo sapiens GN=CA2 PE=1 SV=2							
$70-2			2	1	6.15%	29246.01	6.87	tr|V9HW21|V9HW21_HUMAN Epididymis luminal protein 76 OS=Homo sapiens GN=HEL-76 PE=2 SV=1							
$70-3			2	1	6.15%	29175.96	7.22	tr|A0A0K0K1L1|A0A0K0K1L1_HUMAN Epididymis secretory protein Li 282 OS=Homo sapiens GN=HEL-S-282 PE=2 SV=1							
	R16062_1_KIAA,20460	K.AVQQPDGLAVLGIFLK.V		1669.9891	0.2831	2	1		2.4213	0.4639	352.5	1 14|30	5.88	1	3
	R16062_1_KIAA,20531	K.AVQQPDGLAVLGIFLK.V		1669.9891	1.5271	2	1		2.3884	0.3978	524.2	1 16|30	5.88	1	3
$71-1			2	1	12.05%	18502.47	8.22	sp|P23528|COF1_HUMAN Cofilin-1 OS=Homo sapiens GN=CFL1 PE=1 SV=3							
$71-2			2	1	13.42%	16811.55	8.54	tr|G3V1A4|G3V1A4_HUMAN Cofilin 1 (Non-muscle), isoform CRA_a OS=Homo sapiens GN=CFL1 PE=1 SV=1							
$71-3			2	1	12.05%	18502.47	8.22	tr|V9HWI5|V9HWI5_HUMAN Epididymis secretory protein Li 15 OS=Homo sapiens GN=HEL-S-15 PE=2 SV=1							
$71-4			2	1	25.32%	9090.51	8.5	tr|E9PLJ3|E9PLJ3_HUMAN Cofilin-1 (Fragment) OS=Homo sapiens GN=CFL1 PE=1 SV=1							
$71-5			2	1	12.58%	17777.61	8.22	tr|E9PP50|E9PP50_HUMAN Cofilin-1 (Fragment) OS=Homo sapiens GN=CFL1 PE=1 SV=6							
$71-6			2	1	9.80%	22728.14	8.51	tr|E9PK25|E9PK25_HUMAN Cofilin-1 OS=Homo sapiens GN=CFL1 PE=1 SV=1							
$71-7			2	1	17.86%	12459.53	6.28	tr|B4E112|B4E112_HUMAN cDNA FLJ51435, moderately similar to Cofilin-1 OS=Homo sapiens PE=2 SV=1							

<table>
<tr><td>$71-8</td><td></td><td></td><td>2</td><td>1</td><td>22.22%</td><td>10079.62</td><td>5.5</td><td>tr|E9PS23|E9PS23_HUMAN Cofilin-1 (Fragment) OS=Homo sapiens GN=CFL1 PE=1 SV=6</td><td></td><td></td><td></td><td></td><td></td><td></td><td></td></tr>
<tr><td>$71-9</td><td></td><td></td><td>2</td><td>1</td><td>16.39%</td><td>13923.21</td><td>8.86</td><td>tr|E9PQB7|E9PQB7_HUMAN Cofilin-1 (Fragment) OS=Homo sapiens GN=CFL1 PE=1 SV=1</td><td></td><td></td><td></td><td></td><td></td><td></td><td></td></tr>
<tr><td></td><td>R16062_1_KIAA,16166</td><td>K.EILVGDVGQTVDDPYATFVK.M</td><td></td><td>2167.4008</td><td>0.8078</td><td>2</td><td>1</td><td></td><td>3.5271</td><td>0.5457</td><td>1205</td><td>1 21|38</td><td>3.84</td><td>1</td><td>9</td></tr>
<tr><td></td><td>R16062_1_KIAA,16219</td><td>K.EILVGDVGQTVDDPYATFVK.M</td><td></td><td>2167.4008</td><td>0.8388</td><td>2</td><td>1</td><td></td><td>3.7862</td><td>0.5517</td><td>921.9</td><td>1 21|38</td><td>3.84</td><td>1</td><td>9</td></tr>
<tr><td>$72-1</td><td></td><td></td><td>2</td><td>1</td><td>6.31%</td><td>24722.05</td><td>8.35</td><td>sp|P04179|SODM_HUMAN Superoxide dismutase [Mn], mitochondrial OS=Homo sapiens GN=SOD2 PE=1 SV=2</td><td></td><td></td><td></td><td></td><td></td><td></td><td></td></tr>
<tr><td>$72-2</td><td></td><td></td><td>2</td><td>1</td><td>6.31%</td><td>24750.1</td><td>8.35</td><td>tr|A0A0C4DFU2|A0A0C4DFU2_HUMAN Superoxide dismutase OS=Homo sapiens GN=SOD2 PE=1 SV=1</td><td></td><td></td><td></td><td></td><td></td><td></td><td></td></tr>
<tr><td>$72-3</td><td></td><td></td><td>2</td><td>1</td><td>8.64%</td><td>18261.81</td><td>8.48</td><td>tr|A0A0C4DG56|A0A0C4DG56_HUMAN Superoxide dismutase OS=Homo sapiens GN=SOD2 PE=1 SV=1</td><td></td><td></td><td></td><td></td><td></td><td></td><td></td></tr>
<tr><td>$72-4</td><td></td><td></td><td>2</td><td>1</td><td>6.60%</td><td>23638.65</td><td>7.34</td><td>tr|Q4ZJI1|Q4ZJI1_HUMAN Superoxide dismutase (Fragment) OS=Homo sapiens GN=SOD2 PE=2 SV=1</td><td></td><td></td><td></td><td></td><td></td><td></td><td></td></tr>
<tr><td>$72-5</td><td></td><td></td><td>2</td><td>1</td><td>6.67%</td><td>23224.19</td><td>6.87</td><td>tr|Q7Z7M6|Q7Z7M6_HUMAN Superoxide dismutase (Fragment) OS=Homo sapiens GN=SOD2 PE=2 SV=1</td><td></td><td></td><td></td><td></td><td></td><td></td><td></td></tr>
<tr><td>$72-6</td><td></td><td></td><td>2</td><td>1</td><td>6.57%</td><td>23672.68</td><td>6.87</td><td>tr|Q7Z7M4|Q7Z7M4_HUMAN Superoxide dismutase (Fragment) OS=Homo sapiens GN=SOD2 PE=2 SV=1</td><td></td><td></td><td></td><td></td><td></td><td></td><td></td></tr>
<tr><td>$72-7</td><td></td><td></td><td>2</td><td>1</td><td>7.37%</td><td>21104.8</td><td>9.24</td><td>tr|F5GYZ5|F5GYZ5_HUMAN Superoxide dismutase [Mn], mitochondrial (Fragment) OS=Homo sapiens GN=SOD2 PE=1 SV=1</td><td></td><td></td><td></td><td></td><td></td><td></td><td></td></tr>
<tr><td>$72-8</td><td></td><td></td><td>2</td><td>1</td><td>7.65%</td><td>20019.63</td><td>9.36</td><td>tr|F5H4R2|F5H4R2_HUMAN Superoxide dismutase [Mn], mitochondrial (Fragment) OS=Homo sapiens GN=SOD2 PE=1 SV=1</td><td></td><td></td><td></td><td></td><td></td><td></td><td></td></tr>
<tr><td>$72-9</td><td></td><td></td><td>2</td><td>1</td><td>6.57%</td><td>23558.61</td><td>6.9</td><td>tr|Q7Z7M7|Q7Z7M7_HUMAN Superoxide dismutase (Fragment) OS=Homo sapiens GN=SOD2 PE=2 SV=1</td><td></td><td></td><td></td><td></td><td></td><td></td><td></td></tr>
<tr><td>$72-10</td><td></td><td></td><td>2</td><td>1</td><td>12.61%</td><td>12130.54</td><td>8.05</td><td>tr|F5H3C5|F5H3C5_HUMAN Superoxide dismutase [Mn], mitochondrial (Fragment) OS=Homo sapiens GN=SOD2 PE=1 SV=1</td><td></td><td></td><td></td><td></td><td></td><td></td><td></td></tr>
<tr><td></td><td>R16062_1_KIAA,12225</td><td>K.GDVTAQIALQPALK.F</td><td></td><td>1425.6556</td><td>-0.6684</td><td>2</td><td>1</td><td></td><td>2.3033</td><td>0.2726</td><td>606</td><td>1 13|26</td><td>5.84</td><td>1</td><td>10</td></tr>
<tr><td></td><td>R16062_1_KIAA,12242</td><td>K.GDVTAQIALQPALK.F</td><td></td><td>1425.6556</td><td>0.9096</td><td>2</td><td>1</td><td></td><td>2.4271</td><td>0.2746</td><td>649.6</td><td>2 13|26</td><td>5.84</td><td>1</td><td>10</td></tr>
<tr><td>$73-1</td><td></td><td></td><td>2</td><td>1</td><td>7.47%</td><td>26922.76</td><td>5.09</td><td>sp|O00299|CLIC1_HUMAN Chloride intracellular channel protein 1 OS=Homo sapiens GN=CLIC1 PE=1 SV=4</td><td></td><td></td><td></td><td></td><td></td><td></td><td></td></tr>
<tr><td>$73-2</td><td></td><td></td><td>2</td><td>1</td><td>7.47%</td><td>26922.76</td><td>5.09</td><td>tr|Q5SRT3|Q5SRT3_HUMAN Chloride intracellular channel protein OS=Homo sapiens GN=CLIC1 PE=2 SV=2</td><td></td><td></td><td></td><td></td><td></td><td></td><td></td></tr>
<tr><td>$73-3</td><td></td><td></td><td>2</td><td>1</td><td>7.47%</td><td>27014.86</td><td>5.09</td><td>tr|Q53FB0|Q53FB0_HUMAN Chloride intracellular channel protein (Fragment) OS=Homo sapiens PE=2 SV=1</td><td></td><td></td><td></td><td></td><td></td><td></td><td></td></tr>
</table>

	R16062_1_KIAA,15960	K.LAALNPESNTAGLDIFAK.F		1846.074	0.565	2	1		2.7533	0.372	619.7	1 16|34	4.37	1	3
	R16062_1_KIAA,15970	K.LAALNPESNTAGLDIFAK.F		1846.074	0.823	2	1		2.8014	0.4263	645.2	1 17|34	4.37	1	3
$74-1			2	1	4.23%	39594.9	9.1	sp|P51991|ROA3_HUMAN Heterogeneous nuclear ribonucleoprotein A3 OS=Homo sapiens GN=HNRNPA3 PE=1 SV=2							
$74-2			2	1	4.49%	37028.98	8.46	tr|B4DDB6|B4DDB6_HUMAN Heterogeneous nuclear ribonucleoprotein A3, isoform CRA_a OS=Homo sapiens GN=HNRPA3 PE=2 SV=1							
$74-3			2	1	4.95%	34189.15	8.18	tr|B4E3E6|B4E3E6_HUMAN cDNA FLJ58832, highly similar to Heterogeneous nuclear ribonucleoprotein A3 OS=Homo sapiens PE=2 SV=1							
$74-4			2	1	5.95%	29356.68	8.5	tr|Q65ZQ3|Q65ZQ3_HUMAN FBRNP OS=Homo sapiens GN=D10S102 PE=2 SV=1							
	R16062_1_KIAA,17267	K.LFIGGLSFETTDDSLR.E		1771.9488	0.7728	2	1		2.6808	0.2659	1177.3	1 19|30	4.03	1	4
	R16062_1_KIAA,17346	K.LFIGGLSFETTDDSLR.E		1771.9488	0.2908	2	1		2.8108	0.3798	937.9	1 17|30	4.03	1	4
$75-1			2	1	3.26%	37540.16	6.36	sp|P37837|TALDO_HUMAN Transaldolase OS=Homo sapiens GN=TALDO1 PE=1 SV=2							
$75-2			2	1	3.46%	35328.83	9.07	tr|F2Z393|F2Z393_HUMAN Transaldolase OS=Homo sapiens GN=TALDO1 PE=1 SV=1							
	R16062_1_KIAA,13063	K.LLGELLQDNAK.L		1214.3937	-0.8883	2	1		2.5668	0.3047	680	1 13|20	4.37	1	2
	R16062_1_KIAA,13077	K.LLGELLQDNAK.L		1214.3937	-0.2713	2	1		2.8913	0.4608	1220.1	1 16|20	4.37	1	2
$76-1			2	1	5.02%	35882.74	5.84	sp|P09525|ANXA4_HUMAN Annexin A4 OS=Homo sapiens GN=ANXA4 PE=1 SV=4							
$76-2			2	1	4.98%	36085.01	5.84	tr|Q6LES2|Q6LES2_HUMAN Annexin (Fragment) OS=Homo sapiens GN=ANXA4 PE=2 SV=1							
$76-3			2	1	5.35%	33551.92	5.64	tr|Q6P452|Q6P452_HUMAN Annexin OS=Homo sapiens GN=ANXA4 PE=1 SV=1							
$76-4			2	1	7.11%	25420.97	6.03	tr|Q59FK3|Q59FK3_HUMAN Annexin (Fragment) OS=Homo sapiens PE=2 SV=1							
$76-5			2	1	14.29%	12747.68	8.52	tr|Q6MZI0|Q6MZI0_HUMAN Annexin (Fragment) OS=Homo sapiens GN=DKFZp686H02120 PE=2 SV=1							
$76-6			2	1	5.28%	34459.16	5.65	tr|B4DE02|B4DE02_HUMAN Annexin OS=Homo sapiens PE=2 SV=1							
$76-7			2	1	4.98%	36056.95	5.65	tr|V9HW59|V9HW59_HUMAN Annexin OS=Homo sapiens GN=HEL-S-274 PE=2 SV=1							

$76-8			2	1	5.35%	33613.96	5.64	tr|B4DDZ4|B4DDZ4_HUMAN Annexin OS=Homo sapiens PE=2 SV=1							
	R16062_1_KIAA,19476	K.SETSGSFEDALLAIVK.C		1667.8389	0.4089	2	1		3.0374	0.3639	1025.2	1 16|30	4.14	1	8
	R16062_1_KIAA,19530	K.SETSGSFEDALLAIVK.C		1667.8389	0.6169	2	1		2.9033	0.4402	789.3	1 16|30	4.14	1	8
$77-1			2	1	3.79%	34364.47	5.41	sp|Q8NHW5|RLA0L_HUMAN 60S acidic ribosomal protein P0-like OS=Homo sapiens GN=RPLP0P6 PE=5 SV=1							
$77-2			2	1	3.79%	34273.51	5.72	sp|P05388|RLA0_HUMAN 60S acidic ribosomal protein P0 OS=Homo sapiens GN=RPLP0 PE=1 SV=1							
$77-3			2	1	7.23%	18270.39	9.65	tr|G3V210|G3V210_HUMAN 60S acidic ribosomal protein P0 OS=Homo sapiens GN=RPLP0 PE=1 SV=1							
$77-4			2	1	3.79%	34273.51	5.72	tr|A0A024RBS2|A0A024RBS2_HUMAN 60S acidic ribosomal protein P0 OS=Homo sapiens GN=RPLP0 PE=3 SV=1							
$77-5			2	1	3.79%	34301.53	5.72	tr|Q53HW2|Q53HW2_HUMAN 60S acidic ribosomal protein P0 (Fragment) OS=Homo sapiens PE=2 SV=1							
$77-6			2	1	3.79%	34239.49	5.72	tr|A8K4Z4|A8K4Z4_HUMAN 60S acidic ribosomal protein P0 OS=Homo sapiens PE=2 SV=1							
$77-7			2	1	4.86%	27187.55	9.11	tr|F8VU65|F8VU65_HUMAN 60S acidic ribosomal protein P0 (Fragment) OS=Homo sapiens GN=RPLP0 PE=1 SV=1							
$77-8			2	1	7.84%	16680.51	9.39	tr|F8VPE8|F8VPE8_HUMAN 60S acidic ribosomal protein P0 (Fragment) OS=Homo sapiens GN=RPLP0 PE=1 SV=1							
$77-9			2	1	4.72%	27298.65	8.36	tr|Q6NSF2|Q6NSF2_HUMAN RPLP0 protein OS=Homo sapiens GN=RPLP0 PE=2 SV=1							
$77-10			2	1	4.51%	28538.63	4.69	tr|B4E3D5|B4E3D5_HUMAN cDNA FLJ51469, highly similar to 60S acidic ribosomal protein P0 OS=Homo sapiens PE=2 SV=1							
$77-11			2	1	4.27%	30456.11	5.9	tr|F8VWS0|F8VWS0_HUMAN 60S acidic ribosomal protein P0 OS=Homo sapiens GN=RPLP0 PE=1 SV=1							
$77-12			2	1	4.92%	26901.25	9.05	tr|F8VW21|F8VW21_HUMAN 60S acidic ribosomal protein P0 (Fragment) OS=Homo sapiens GN=RPLP0 PE=1 SV=1							
	R16062_1_KIAA,17427	K.TSFFQALGITTK.I		1314.5117	0.3797	2	1		2.4005	0.5168	999.2	1 14|22	8.41	1	12
	R16062_1_KIAA,17482	K.TSFFQALGITTK.I		1314.5117	0.3687	2	1		3.2542	0.5068	895.2	1 14|22	8.41	1	12
$78-1			2	1	4.89%	29995.51	10.61	sp|P62424|RL7A_HUMAN 60S ribosomal protein L7a OS=Homo sapiens GN=RPL7A PE=1 SV=2							
$78-2			2	1	6.81%	21544.69	11.02	tr|Q5T8U3|Q5T8U3_HUMAN 60S ribosomal protein L7a (Fragment) OS=Homo sapiens GN=RPL7A PE=1 SV=1							
$78-3			2	1	8.61%	16539.45	10.01	tr|Q5T8U2|Q5T8U2_HUMAN 60S ribosomal protein L7a OS=Homo sapiens GN=RPL7A PE=1 SV=1							
$78-4			2	1	9.15%	15636.38	10.06	tr|Q9BY74|Q9BY74_HUMAN Ribosomal protein L7a (Fragment)							

								OS=Homo sapiens GN=RP-L7a PE=2 SV=1							
	R16062_1_KIAA,11410	R.AGVNTVTTLVENK.K		1346.5121	0.3131	2	1		2.798	0.5118	718.5	1 14|24	6.05	1	4
	R16062_1_KIAA,11431	R.AGVNTVTTLVENK.K		1346.5121	1.0751	2	1		3.273	0.4627	1057.9	1 16|24	6.05	1	4
$79-1			2	1	4.27%	31121.92	4.9	sp|P29692|EF1D_HUMAN Elongation factor 1-delta OS=Homo sapiens GN=EEF1D PE=1 SV=5							
$79-2			2	1	7.23%	18157.05	4.97	tr|E9PPR1|E9PPR1_HUMAN Elongation factor 1-delta (Fragment) OS=Homo sapiens GN=EEF1D PE=1 SV=1							
$79-3			2	1	6.06%	21824.3	5.38	tr|E9PQ49|E9PQ49_HUMAN Elongation factor 1-delta (Fragment) OS=Homo sapiens GN=EEF1D PE=1 SV=6							
$79-4			2	1	4.60%	28821.32	4.93	tr|E9PK01|E9PK01_HUMAN Elongation factor 1-delta (Fragment) OS=Homo sapiens GN=EEF1D PE=1 SV=1							
$79-5			2	1	6.42%	20819.11	4.9	tr|E9PL71|E9PL71_HUMAN Elongation factor 1-delta (Fragment) OS=Homo sapiens GN=EEF1D PE=1 SV=1							
$79-6			2	1	9.23%	14179.82	6.84	tr|E9PMW7|E9PMW7_HUMAN Elongation factor 1-delta OS=Homo sapiens GN=EEF1D PE=1 SV=1							
$79-7			2	1	5.88%	22490.13	5.38	tr|E9PI39|E9PI39_HUMAN Elongation factor 1-delta (Fragment) OS=Homo sapiens GN=EEF1D PE=1 SV=1							
$79-8			2	1	1.90%	69282.71	6.76	tr|A0A087X1X7|A0A087X1X7_HUMAN Elongation factor 1-delta OS=Homo sapiens GN=EEF1D PE=1 SV=1							
$79-9			2	1	7.14%	18401.26	4.82	tr|E9PL12|E9PL12_HUMAN Elongation factor 1-delta (Fragment) OS=Homo sapiens GN=EEF1D PE=1 SV=1							
$79-10			2	1	2.18%	60882.77	7.48	tr|Q9BW34|Q9BW34_HUMAN EEF1D protein (Fragment) OS=Homo sapiens GN=EEF1D PE=2 SV=2							
$79-11			2	1	1.85%	71450.24	6.02	tr|B2RAR6|B2RAR6_HUMAN cDNA, FLJ95068, highly similar to Homo sapiens eukaryotic translation elongation factor 1 delta (guanine nucleotide exchange protein) (EEF1D), transcript variant 1, mRNA OS=Homo sapiens PE=2 SV=1							
$79-12			2	1	1.85%	71422.19	6.02	tr|D3DWK1|D3DWK1_HUMAN Eukaryotic translation elongation factor 1 delta (Guanine nucleotide exchange protein), isoform CRA_b OS=Homo sapiens GN=EEF1D PE=3 SV=1							
$79-13			2	1	8.76%	15001.89	8.85	tr|E9PIZ1|E9PIZ1_HUMAN Elongation factor 1-delta (Fragment) OS=Homo sapiens GN=EEF1D PE=1 SV=1							
$79-14			2	1	1.90%	69395.87	6.76	tr|Q71RH4|Q71RH4_HUMAN FP1047 OS=Homo sapiens PE=2 SV=1							
$79-15			2	1	1.85%	71418.3	6.23	tr|Q9H7G6|Q9H7G6_HUMAN cDNA: FLJ20897 fis, clone ADKA03573 OS=Homo sapiens PE=2 SV=1							
$79-16			2	1	1.72%	76570.37	6.61	tr|E9PRY8|E9PRY8_HUMAN Elongation factor 1-delta OS=Homo sapiens GN=EEF1D PE=1 SV=1							
$79-1			2	1	5.71%	22836.7	9.05	tr|H0YCK7|H0YCK7_HUMAN Elongation factor 1-delta							

7								(Fragment) OS=Homo sapiens GN=EEF1D PE=1 SV=1							
$79-18			2	1	11.32%	11615.91	6.28	tr|E9PN91|E9PN91_HUMAN Elongation factor 1-delta OS=Homo sapiens GN=EEF1D PE=1 SV=1							
	R16062_1_KIAA,15679	R.GVVQELQQAISK.L		1300.4866	0.2096	2	1		2.5926	0.4084	894.8	1 14|22	6	1	18
	R16062_1_KIAA,15740	R.GVVQELQQAISK.L		1300.4866	0.1206	2	1		2.2135	0.3556	635.4	1 12|22	6	1	18
$80-1			2	1	3.50%	32922.77	5.45	sp|P52907|CAZA1_HUMAN F-actin-capping protein subunit alpha-1 OS=Homo sapiens GN=CAPZA1 PE=1 SV=3							
$80-2			2	1	3.50%	32949.08	5.57	sp|P47755|CAZA2_HUMAN F-actin-capping protein subunit alpha-2 OS=Homo sapiens GN=CAPZA2 PE=1 SV=3							
$80-3			2	1	3.50%	32922.77	5.45	tr|A0A024R0E5|A0A024R0E5_HUMAN Capping protein (Actin filament) muscle Z-line, alpha 1, isoform CRA_a OS=Homo sapiens GN=CAPZA1 PE=4 SV=1							
$80-4			2	1	3.50%	32949.08	5.57	tr|A4D0V4|A4D0V4_HUMAN Capping protein (Actin filament) muscle Z-line, alpha 2 OS=Homo sapiens GN=CAPZA2 PE=2 SV=1							
$80-5			2	1	3.50%	32967.11	5.57	tr|Q53GC7|Q53GC7_HUMAN Capping protein (Actin filament) muscle Z-line, alpha 2 variant (Fragment) OS=Homo sapiens PE=2 SV=1							
$80-6			2	1	5.75%	20080.88	6.07	tr|A0A0D9SET8|A0A0D9SET8_HUMAN F-actin-capping protein subunit alpha-2 OS=Homo sapiens GN=CAPZA2 PE=1 SV=1							
$80-7			2	1	6.85%	16698.71	5	tr|C9JUG7|C9JUG7_HUMAN F-actin-capping protein subunit alpha-2 OS=Homo sapiens GN=CAPZA2 PE=1 SV=1							
$80-8			2	1	3.50%	32967.11	5.57	tr|Q53GE2|Q53GE2_HUMAN Capping protein (Actin filament) muscle Z-line, alpha 2 variant (Fragment) OS=Homo sapiens PE=2 SV=1							
$80-9			2	1	3.50%	32908.74	5.44	tr|A8K0T9|A8K0T9_HUMAN cDNA FLJ75422, highly similar to Homo sapiens capping protein (actin filament) muscle Z-line, alpha 1, mRNA OS=Homo sapiens PE=2 SV=1							
$80-10			2	1	5.75%	19963.56	5.14	tr|F8W9N7|F8W9N7_HUMAN F-actin-capping protein subunit alpha-2 OS=Homo sapiens GN=CAPZA2 PE=1 SV=1							
	R16062_1_KIAA,13638	R.LLLNNDNLLR.E		1198.3975	-0.4025	2	1		2.3746	0.3255	874.3	1 14|18	5.84	1	10
	R16062_1_KIAA,13669	R.LLLNNDNLLR.E		1198.3975	-1.5415	2	1		2.6166	0.1714	1184.2	1 15|18	5.84	1	10
$81-1			2	1	8.25%	22119.27	7.13	sp|P30043|BLVRB_HUMAN Flavin reductase (NADPH) OS=Homo sapiens GN=BLVRB PE=1 SV=3							
$81-2			2	1	10.43%	17160.36	6.27	tr|M0R192|M0R192_HUMAN Flavin reductase (NADPH) OS=Homo sapiens GN=BLVRB PE=1 SV=1							
$81-3			2	1	8.25%	22119.27	7.13	tr|V9HWI1|V9HWI1_HUMAN Epididymis secretory protein Li 10							

								OS=Homo sapiens GN=HEL-S-10 PE=2 SV=1								
$81-4			2	1	11.04%	15669.7	5.22	tr|M0QZL1|M0QZL1_HUMAN Flavin reductase (NADPH) OS=Homo sapiens GN=BLVRB PE=1 SV=1								
	R16062_1_KIAA,12837	R.PAHVVVGDVLQAADVDK.T		1733.9469	0.4289	2	1		2.9331	0.4936	2036.5	1	22|32	4.41	1	4
	R16062_1_KIAA,12921	R.PAHVVVGDVLQAADVDK.T		1733.9469	0.2369	2	1		2.5767	0.4136	457.9	1	13|32	4.41	1	4
$82-1			1	1	16.67%	11834.32	8.94	tr|Q9UL83|Q9UL83_HUMAN Myosin-reactive immunoglobulin light chain variable region (Fragment) OS=Homo sapiens PE=2 SV=1								
$82-2			1	1	16.51%	11761.2	8.74	tr|Q9UL85|Q9UL85_HUMAN Myosin-reactive immunoglobulin kappa chain variable region (Fragment) OS=Homo sapiens PE=2 SV=1								
	R16062_1_KIAA,10455	-.EIVM*TQSPATLSVSPGER.A		1919.1468	0.5848	2	1		2.6633	0.3593	573.7	1	15|34	4.53	1	2
$83-1			1	1	14.50%	14142.08	9.65	tr|Q9UL88|Q9UL88_HUMAN Myosin-reactive immunoglobulin heavy chain variable region (Fragment) OS=Homo sapiens PE=2 SV=1								
$83-2			1	1	15.83%	13226.73	6.44	sp|P01766|HV305_HUMAN Ig heavy chain V-III region BRO OS=Homo sapiens PE=1 SV=1								
$83-3			1	1	15.97%	12559.17	8.72	sp|P01777|HV316_HUMAN Ig heavy chain V-III region TEI OS=Homo sapiens PE=1 SV=1								
$83-4			1	1	18.81%	11167.39	8.05	tr|A0A087WW89|A0A087WW89_HUMAN Protein IGHV3-72 OS=Homo sapiens GN=IGHV3-72 PE=1 SV=1								
$83-5			1	1	16.10%	12829.36	5.71	tr|A0A068LKR4|A0A068LKR4_HUMAN Ig heavy chain variable region (Fragment) OS=Homo sapiens PE=2 SV=1								
$83-6			1	1	19.39%	10656.78	8.66	tr|A0A0B4J2B5|A0A0B4J2B5_HUMAN Protein IGHV3OR16-9 (Fragment) OS=Homo sapiens GN=IGHV3OR16-9 PE=1 SV=1								
$83-7			1	1	16.10%	12843.4	5.25	tr|Q9UL91|Q9UL91_HUMAN Myosin-reactive immunoglobulin heavy chain variable region (Fragment) OS=Homo sapiens PE=2 SV=1								
$83-8			1	1	15.70%	13435.97	8.65	tr|Q0ZCH9|Q0ZCH9_HUMAN Immunglobulin heavy chain variable region (Fragment) OS=Homo sapiens PE=4 SV=1								
$83-9			1	1	19.79%	10429.48	8.05	tr|S4R460|S4R460_HUMAN Protein IGHV3OR16-9 OS=Homo sapiens GN=IGHV3OR16-9 PE=1 SV=2								
$83-10			1	1	16.10%	12872.26	6.48	tr|Q9UL72|Q9UL72_HUMAN Myosin-reactive immunoglobulin heavy chain variable region (Fragment) OS=Homo sapiens PE=2 SV=1								
	R16062_1_KIAA,11632	-.EVQLVESGGGLVKPGGSLR.L ! -.EVQLVESGGGLVQPGGSLR.L		1883.139	-0.12	2	1		2.597	0.1058	1010.8	1	20|36	6.24	1	10
$84-1			1	1	15.32%	13650.22	7.92	tr|Q0ZCJ2|Q0ZCJ2_HUMAN Immunglobulin heavy chain variable								

								region (Fragment) OS=Homo sapiens PE=4 SV=1							
$84-2			1	1	16.24%	12725.14	5.28	tr|Q0ZCH7|Q0ZCH7_HUMAN Immunglobulin heavy chain variable region (Fragment) OS=Homo sapiens PE=4 SV=1							
$84-3			1	1	16.24%	13000.59	7.94	tr|Q0ZCH4|Q0ZCH4_HUMAN Immunglobulin heavy chain variable region (Fragment) OS=Homo sapiens PE=4 SV=1							
	R16062_1_KIAA,14042	-.LVQLVESGGGLVQPGGSLR.L		1867.1396	2.4796	2	1		2.3508	0.3981	1060.7	1 18|36	6	1	3
$85-1			1	1	19.39%	10737.07	8.98	tr|A2J1N2|A2J1N2_HUMAN Rheumatoid factor RF-IP18 (Fragment) OS=Homo sapiens PE=2 SV=1							
$85-2			1	1	15.20%	13944.55	9.1	tr|A0A068LKR7|A0A068LKR7_HUMAN Ig heavy chain variable region (Fragment) OS=Homo sapiens PE=2 SV=1							
	R16062_1_KIAA,12720	-.QVQLVESGGGLVKPGGSLR.L		1882.1543	-0.3687	2	1		2.2007	0.1771	417.1	1 15|36	8.75	1	2
$86-1			1	1	4.08%	44614.53	8.3	sp|P00558|PGK1_HUMAN Phosphoglycerate kinase 1 OS=Homo sapiens GN=PGK1 PE=1 SV=3							
$86-2			1	1	4.08%	44614.53	8.3	tr|V9HWF4|V9HWF4_HUMAN Phosphoglycerate kinase OS=Homo sapiens GN=HEL-S-68p PE=2 SV=1							
	R16062_1_KIAA,16905	K.ACANPAAGSVILLENLR.F		1770.0174	1.3884	2	1		2.306	0.3872	598.1	1 16|32	6.04	1	2
$87-1			1	1	4.37%	44552.24	6.1	sp|P07339|CATD_HUMAN Cathepsin D OS=Homo sapiens GN=CTSD PE=1 SV=1							
$87-2			1	1	4.37%	44552.24	6.1	tr|V9HWI3|V9HWI3_HUMAN Cathepsin D (Lysosomal aspartyl peptidase), isoform CRA_a OS=Homo sapiens GN=HEL-S-130P PE=2 SV=1							
$87-3			1	1	6.90%	27672.88	6.16	tr|H7C469|H7C469_HUMAN Uncharacterized protein (Fragment) OS=Homo sapiens PE=1 SV=5							
$87-4			1	1	9.52%	20371.41	8.7	tr|H7C1V0|H7C1V0_HUMAN Cathepsin D (Fragment) OS=Homo sapiens GN=CTSD PE=1 SV=1							
	R16062_1_KIAA,14883	K.AIGAVPLIQGEYM*IPCEK.V		2006.3458	0.9868	2	1		2.3099	0.4296	773.1	1 17|34	4.53	1	4
$88-1			1	1	10.00%	11283.86	6.09	sp|P81605|DCD_HUMAN Dermcidin OS=Homo sapiens GN=DCD PE=1 SV=2							
	R16062_1_KIAA,11629	K.DAVEDLESVGK.G		1162.2293	0.5333	2	1		2.583	0.3402	995.7	1 14|20	3.92	1	1
$89-1			1	1	8.45%	15257.51	8.72	sp|P69905|HBA_HUMAN Hemoglobin subunit alpha OS=Homo sapiens GN=HBA1 PE=1 SV=2							
$89-2			1	1	14.12%	9078.48	8.16	tr|P78461|P78461_HUMAN Alpha-1 thalassemia globin gene (Fragment) OS=Homo sapiens PE=3 SV=1							
$89-3			1	1	28.57%	4522.32	8.2	tr|U6A493|U6A493_HUMAN Mutant hemoglobin alpha 1 globin chain (Fragment) OS=Homo sapiens GN=HBA2 PE=3 SV=1							
$89-4			1	1	10.91%	11947.77	8.82	tr|G3V1N2|G3V1N2_HUMAN HCG1745306, isoform CRA_a							

								OS=Homo sapiens GN=HBA2 PE=1 SV=1							
$89-5			1	1	8.45%	15257.51	8.72	tr|D1MGQ2|D1MGQ2_HUMAN Alpha-2 globin chain OS=Homo sapiens GN=HBA2 PE=3 SV=1							
$89-6			1	1	13.64%	9491.95	8.15	tr|Q86YL2|Q86YL2_HUMAN Hemoglobin alpha-1 chain (Fragment) OS=Homo sapiens GN=HBA1 PE=3 SV=1							
$89-7			1	1	13.19%	9671.17	9.31	tr|Q86YQ1|Q86YQ1_HUMAN Hemoglobin alpha-2 (Fragment) OS=Homo sapiens GN=HBA2 PE=3 SV=1							
$89-8			1	1	8.45%	15279.56	9.07	tr|I1VZV6|I1VZV6_HUMAN Hemoglobin alpha 1 OS=Homo sapiens GN=HBA1 PE=3 SV=1							
$89-9			1	1	27.91%	4635.48	8.2	tr|Q7Z7B4|Q7Z7B4_HUMAN Truncated alpha 1 globin variant (Fragment) OS=Homo sapiens PE=3 SV=1							
$89-10			1	1	13.64%	9482	9.26	tr|Q86YQ4|Q86YQ4_HUMAN Alpha-1 globin (Fragment) OS=Homo sapiens GN=HBA1 PE=3 SV=1							
$89-11			1	1	21.43%	6226.37	9.75	tr|Q86YQ5|Q86YQ5_HUMAN Alpha-2 globin (Fragment) OS=Homo sapiens GN=HBA2 PE=3 SV=1							
$89-12			1	1	13.19%	9627.07	8.14	tr|Q9NQT3|Q9NQT3_HUMAN Alpha one globin (Fragment) OS=Homo sapiens GN=HBA1 PE=2 SV=1							
$89-13			1	1	29.27%	4366.13	7.01	tr|U6A3P2|U6A3P2_HUMAN Mutant hemoglobin alpha 2 globin chain (Fragment) OS=Homo sapiens GN=HBA2 PE=3 SV=1							
$89-14			1	1	9.38%	13582.49	7.97	tr|V9H1D9|V9H1D9_HUMAN Alpha globin OS=Homo sapiens PE=3 SV=1							
$89-15			1	1	8.45%	15257.51	8.72	tr|A0A0K2BMD8|A0A0K2BMD8_HUMAN Mutant hemoglobin alpha 2 globin chain OS=Homo sapiens GN=HBA2 PE=3 SV=1							
$89-16			1	1	13.33%	9591.12	9.52	tr|Q6J1Z9|Q6J1Z9_HUMAN Hemoglobin alpha 1 (Fragment) OS=Homo sapiens GN=HBA1 PE=3 SV=1							
	R16062_1_KIAA,13637	K.FLASVSTVLTSK.Y		1253.4698	0.6638	2	1		2.3328	0.384	673.2	1 13|22	8.75	1	16
$90-1			1	1	2.59%	50976.43	5.39	sp|P61978|HNRPK_HUMAN Heterogeneous nuclear ribonucleoprotein K OS=Homo sapiens GN=HNRNPK PE=1 SV=1							
$90-2			1	1	2.59%	51028.42	5.19	tr|Q6IBN1|Q6IBN1_HUMAN HNRPK protein OS=Homo sapiens GN=HNRPK PE=2 SV=1							
$90-3			1	1	2.59%	51218.76	5.46	tr|A0A024R228|A0A024R228_HUMAN Heterogeneous nuclear ribonucleoprotein K, isoform CRA_d OS=Homo sapiens GN=HNRPK PE=4 SV=1							
$90-4			1	1	3.17%	41807.17	5.43	tr|Q5T6W2|Q5T6W2_HUMAN Heterogeneous nuclear ribonucleoprotein K (Fragment) OS=Homo sapiens GN=HNRNPK PE=1 SV=1							
$90-5			1	1	2.59%	51058.44	5.19	tr|Q5EC54|Q5EC54_HUMAN Heterogeneous nuclear ribonucleoprotein K transcript variant OS=Homo sapiens GN=HNRPK PE=2 SV=1							

$90-6			1	1	2.61%	50715.23	5.18	tr|B4DFF1|B4DFF1_HUMAN cDNA FLJ53312, highly similar to Heterogeneous nuclear ribonucleoprotein K OS=Homo sapiens PE=2 SV=1								
$90-7			1	1	2.73%	48510.66	5.69	tr|B4DUQ1|B4DUQ1_HUMAN cDNA FLJ54552, highly similar to Heterogeneous nuclear ribonucleoprotein K OS=Homo sapiens PE=2 SV=1								
	R16062_1_KIAA,17049	K.IILDLISESPIK.G		1341.6185	0.8125	2	1		2.9329	0.368	1417.8	1	15|22	4.37	1	7
$91-1			1	1	6.91%	21634.46	11.73	sp|Q07020|RL18_HUMAN 60S ribosomal protein L18 OS=Homo sapiens GN=RPL18 PE=1 SV=2								
$91-2			1	1	6.91%	21634.46	11.73	tr|A0A024QZD1|A0A024QZD1_HUMAN Ribosomal protein L18, isoform CRA_c OS=Homo sapiens GN=RPL18 PE=4 SV=1								
$91-3			1	1	7.93%	18756.14	11.59	tr|G3V203|G3V203_HUMAN 60S ribosomal protein L18 OS=Homo sapiens GN=RPL18 PE=1 SV=1								
$91-4			1	1	6.84%	21728.52	11.73	tr|J3QQ67|J3QQ67_HUMAN 60S ribosomal protein L18 (Fragment) OS=Homo sapiens GN=RPL18 PE=1 SV=1								
$91-5			1	1	10.00%	14529.08	11.75	tr|F8VUA6|F8VUA6_HUMAN 60S ribosomal protein L18 (Fragment) OS=Homo sapiens GN=RPL18 PE=1 SV=1								
$91-6			1	1	7.93%	18732.14	11.82	tr|Q0QEW2|Q0QEW2_HUMAN Ribosomal protein L18 (Fragment) OS=Homo sapiens GN=RPL18 PE=2 SV=1								
$91-7			1	1	7.78%	18863.2	11.77	tr|H0YHA7|H0YHA7_HUMAN 60S ribosomal protein L18 (Fragment) OS=Homo sapiens GN=RPL18 PE=1 SV=1								
	R16062_1_KIAA,15934	K.ILTFDQLALDSPK.G		1461.6847	0.5707	2	1		2.5939	0.1896	1042.8	1	16|24	4.21	1	7
$92-1			1	1	4.02%	25035.01	6	sp|P30041|PRDX6_HUMAN Peroxiredoxin-6 OS=Homo sapiens GN=PRDX6 PE=1 SV=3								
$92-2			1	1	4.02%	25035.01	6	tr|V9HWC7|V9HWC7_HUMAN Epididymis secretory sperm binding protein Li 128m OS=Homo sapiens GN=HEL-S-128m PE=2 SV=1								
$92-3			1	1	11.25%	8794.32	5.04	tr|A4UCS6|A4UCS6_HUMAN Peroxiredoxin 6 (Fragment) OS=Homo sapiens PE=2 SV=1								
	R16062_1_KIAA,15140	K.LPFPIIDDR.N		1086.2656	0.1906	2	1		2.4273	0.4612	740.1	3	12|16	4.21	1	3
$93-1			1	1	10.43%	12784	9.65	sp|P62888|RL30_HUMAN 60S ribosomal protein L30 OS=Homo sapiens GN=RPL30 PE=1 SV=2								
$93-2			1	1	21.43%	6262.62	10.67	tr|E5RJH3|E5RJH3_HUMAN 60S ribosomal protein L30 OS=Homo sapiens GN=RPL30 PE=1 SV=1								
$93-3			1	1	10.43%	12784	9.65	tr|A0A024R9D3|A0A024R9D3_HUMAN Ribosomal protein L30, isoform CRA_b OS=Homo sapiens GN=RPL30 PE=3 SV=1								
$93-4			1	1	10.53%	12655.83	9.59	tr|E5RI99|E5RI99_HUMAN 60S ribosomal protein L30 (Fragment) OS=Homo sapiens GN=RPL30 PE=1 SV=1								

	R16062_1_KIAA,12622	K.LVILANNCPALR.K		1354.6172	0.7672	2	1		2.4734	0.2941	868.8	1 14|22	8.25	1	4
$94-1			1	1	3.25%	51676.43	6.55	sp|P02790|HEMO_HUMAN Hemopexin OS=Homo sapiens GN=HPX PE=1 SV=2							
	R16062_1_KIAA,18249	K.LYLVQGTQVYVFLTK.G		1773.1078	-0.7042	2	1		3.2723	0.4053	628.4	1 15|28	8.5	1	1
$95-1			1	1	3.14%	43810.07	9.47	sp|P51888|PRELP_HUMAN Prolargin OS=Homo sapiens GN=PRELP PE=1 SV=1							
$95-2			1	1	5.31%	26038.22	9.27	tr|Q7Z4B2|Q7Z4B2_HUMAN MSTP161 OS=Homo sapiens GN=MST161 PE=2 SV=1							
$95-3			1	1	3.14%	43783.05	9.47	tr|Q6FHG6|Q6FHG6_HUMAN PRELP protein (Fragment) OS=Homo sapiens GN=PRELP PE=2 SV=1							
	R16062_1_KIAA,11369	K.NQLEEVPSALPR.N		1353.5063	0.2793	2	1		2.6933	0.5067	1327.1	1 17|22	4.53	1	3
$96-1			1	1	8.90%	21258.59	6.16	sp|P60953|CDC42_HUMAN Cell division control protein 42 homolog OS=Homo sapiens GN=CDC42 PE=1 SV=2							
$96-2			1	1	8.90%	21258.59	6.16	tr|A0A024RAE4|A0A024RAE4_HUMAN Cell division cycle 42 (GTP binding protein, 25kDa), isoform CRA_a OS=Homo sapiens GN=CDC42 PE=3 SV=1							
$96-3			1	1	8.90%	21272.62	6.73	tr|A0A024R9T1|A0A024R9T1_HUMAN HCG39634, isoform CRA_a OS=Homo sapiens GN=hCG_39634 PE=3 SV=1							
$96-4			1	1	7.20%	26528.26	7.52	tr|B4E1U9|B4E1U9_HUMAN cDNA FLJ54776, highly similar to Cell division control protein 42 homolog OS=Homo sapiens PE=2 SV=1							
$96-5			1	1	7.30%	26519.18	6.89	tr|B7ZAY4|B7ZAY4_HUMAN cDNA, FLJ79348, highly similar to Cell division control protein 42 homolog OS=Homo sapiens PE=2 SV=1							
$96-6			1	1	7.30%	26579.19	6.44	tr|B4DMH5|B4DMH5_HUMAN cDNA FLJ55107, highly similar to Cell division control protein 42 homolog OS=Homo sapiens PE=2 SV=1							
	R16062_1_KIAA,19951	K.NVFDEAILAALEPPEPK.K		1854.0929	0.9249	3	1		4.1327	0.5197	1150.2	1 30|64	4	1	6
$97-1			1	1	2.17%	85697.56	5.9	sp|P06396|GELS_HUMAN Gelsolin OS=Homo sapiens GN=GSN PE=1 SV=1							
$97-2			1	1	2.27%	82525.62	5.43	tr|A0A0A0MS51|A0A0A0MS51_HUMAN Gelsolin OS=Homo sapiens GN=GSN PE=1 SV=1							
$97-3			1	1	2.22%	84745.33	5.57	tr|A0A0A0MT01|A0A0A0MT01_HUMAN Gelsolin OS=Homo sapiens GN=GSN PE=1 SV=1							
$97-4			1	1	2.22%	84766.38	5.57	tr|B7Z6N2|B7Z6N2_HUMAN cDNA FLJ56154, highly similar to Gelsolin OS=Homo sapiens PE=2 SV=1							
$97-5			1	1	2.47%	75751.99	5.56	tr|B7Z4U6|B7Z4U6_HUMAN cDNA FLJ55803, highly similar to							

								Gelsolin OS=Homo sapiens PE=2 SV=1							
$97-6			1	1	2.25%	83103.47	5.58	tr|B7Z9A0|B7Z9A0_HUMAN cDNA FLJ56212, highly similar to Gelsolin OS=Homo sapiens PE=2 SV=1							
$97-7			1	1	2.38%	78831.6	5.77	tr|B7Z992|B7Z992_HUMAN cDNA FLJ53698, highly similar to Gelsolin OS=Homo sapiens PE=2 SV=1							
$97-8			1	1	3.51%	52372.78	5.21	tr|Q5T0H9|Q5T0H9_HUMAN Gelsolin OS=Homo sapiens GN=GSN PE=1 SV=1							
$97-9			1	1	2.41%	77789.43	5.47	tr|B7Z2X4|B7Z2X4_HUMAN cDNA FLJ53327, highly similar to Gelsolin OS=Homo sapiens PE=2 SV=1							
	R16062_1_KIAA,14564	K.QTQVSVLPEGGETPLFK.Q		1831.0594	0.3764	2	1		2.4122	0.3378	551.6	1 16|32	4.53	1	9
$98-1			1	1	11.11%	12772.57	8.46	tr|A0A0J9YXX1|A0A0J9YXX1_HUMAN Uncharacterized protein (Fragment) OS=Homo sapiens PE=1 SV=1							
$98-2			1	1	11.11%	12674.54	8.45	tr|A0A0C4DH38|A0A0C4DH38_HUMAN Protein IGHV5-51 (Fragment) OS=Homo sapiens GN=IGHV5-51 PE=1 SV=1							
$98-3			1	1	11.82%	12149.58	8.53	tr|A0A0F7TAV4|A0A0F7TAV4_HUMAN IGHV5-51 protein (Fragment) OS=Homo sapiens GN=IGHV5-51 PE=4 SV=1							
	R16062_1_KIAA,15572	K.SISTAYLQWSSLK.A		1484.6784	0.4024	2	1		2.493	0.4136	761.4	1 13|24	8.31	1	3
$99-1			1	1	4.49%	36638.37	5.71	sp|P07195|LDHB_HUMAN L-lactate dehydrogenase B chain OS=Homo sapiens GN=LDHB PE=1 SV=2							
$99-2			1	1	4.49%	36638.37	5.71	tr|Q5U077|Q5U077_HUMAN L-lactate dehydrogenase OS=Homo sapiens GN=LDHB PE=2 SV=1							
$99-3			1	1	11.03%	14734.25	5.4	tr|C9J7H8|C9J7H8_HUMAN L-lactate dehydrogenase B chain (Fragment) OS=Homo sapiens GN=LDHB PE=1 SV=6							
$99-4			1	1	14.71%	10806.58	5.02	tr|F5H793|F5H793_HUMAN L-lactate dehydrogenase B chain (Fragment) OS=Homo sapiens GN=LDHB PE=1 SV=1							
$99-5			1	1	6.47%	25218	5.52	tr|A8MW50|A8MW50_HUMAN L-lactate dehydrogenase (Fragment) OS=Homo sapiens GN=LDHB PE=1 SV=1							
	R16062_1_KIAA,19453	K.SLADELALVDVLEDK.L		1630.8185	0.5955	2	1		2.693	0.3882	748	1 16|28	3.77	1	5
$100-1			1	1	5.15%	15508.25	11.13	sp|Q16695|H31T_HUMAN Histone H3.1t OS=Homo sapiens GN=HIST3H3 PE=1 SV=3							
$100-2			1	1	5.15%	15404.09	11.13	sp|P68431|H31_HUMAN Histone H3.1 OS=Homo sapiens GN=HIST1H3A PE=1 SV=2							
$100-3			1	1	5.15%	15388.03	11.27	sp|Q71DI3|H32_HUMAN Histone H3.2 OS=Homo sapiens GN=HIST2H3A PE=1 SV=3							
$100-4			1	1	5.15%	15327.92	11.27	sp|P84243|H33_HUMAN Histone H3.3 OS=Homo sapiens GN=H3F3A PE=1 SV=2							
$100-5			1	1	5.19%	15213.74	11.11	sp|Q6NXT2|H3C_HUMAN Histone H3.3C OS=Homo sapiens GN=H3F3C PE=1 SV=3							

$100-6			1	1	5.69%	14052.6	11.34	tr|B4DEB1|B4DEB1_HUMAN Histone H3 OS=Homo sapiens GN=H3F3A PE=1 SV=1								
$100-7			1	1	5.15%	15430.11	11.27	tr|Q5TEC6|Q5TEC6_HUMAN Histone H3 OS=Homo sapiens GN=HIST2H3PS2 PE=1 SV=1								
$100-8			1	1	5.15%	15327.92	11.27	tr|B2R4P9|B2R4P9_HUMAN Histone H3 OS=Homo sapiens GN=H3F3A PE=2 SV=1								
$100-9			1	1	5.15%	15313.85	11.26	tr|B2R6Y1|B2R6Y1_HUMAN Histone H3 OS=Homo sapiens PE=2 SV=1								
$100-10			1	1	4.64%	16621.42	11.84	tr|K7ES00|K7ES00_HUMAN Histone H3.3 (Fragment) OS=Homo sapiens GN=H3F3B PE=1 SV=1								
$100-11			1	1	5.30%	14914.48	11.29	tr|K7EK07|K7EK07_HUMAN Histone H3 (Fragment) OS=Homo sapiens GN=H3F3B PE=1 SV=1								
$100-12			1	1	7.61%	10334.07	11.82	tr|K7EMV3|K7EMV3_HUMAN Histone H3 OS=Homo sapiens GN=H3F3B PE=1 SV=1								
$100-13			1	1	6.19%	12918.21	11.92	tr|B4E380|B4E380_HUMAN Histone H3 OS=Homo sapiens PE=2 SV=1								
$100-14			1	1	6.19%	12808.02	11.49	tr|K7EP01|K7EP01_HUMAN Histone H3.3 OS=Homo sapiens GN=H3F3B PE=1 SV=1								
	R16062_1_KIAA,10159	K.STELLIR.K		831.9803	0.4573	2	1		2.2893	0.2375	557.5	4	10|12	5.72	1	14
$101-1			1	1	52.17%	2564.98	4.68	tr|A8CGI2|A8CGI2_HUMAN Ubiquitin C (Fragment) OS=Homo sapiens GN=UBC PE=2 SV=1								
	R16062_1_KIAA,10387	K.TITLEVEPSDTI.-		1318.4523	-0.4787	2	1		2.5963	0.1048	429.2	12	11|22	3.57	1	1
$102-1			1	1	3.45%	28870.18	6.59	sp|P00915|CAH1_HUMAN Carbonic anhydrase 1 OS=Homo sapiens GN=CA1 PE=1 SV=2								
$102-2			1	1	3.45%	28870.18	6.59	tr|V9HWE3|V9HWE3_HUMAN Carbonic anhydrase I, isoform CRA_a OS=Homo sapiens GN=HEL-S-11 PE=2 SV=1								
$102-3			1	1	5.06%	19697.03	8.06	tr|H0YBE2|H0YBE2_HUMAN Carbonic anhydrase 1 (Fragment) OS=Homo sapiens GN=CA1 PE=1 SV=1								
$102-4			1	1	6.57%	15041.25	8.74	tr|E5RJI8|E5RJI8_HUMAN Carbonic anhydrase 1 (Fragment) OS=Homo sapiens GN=CA1 PE=1 SV=6								
$102-5			1	1	4.64%	21402.04	7.1	tr|E5RFE7|E5RFE7_HUMAN Carbonic anhydrase 1 (Fragment) OS=Homo sapiens GN=CA1 PE=1 SV=1								
$102-6			1	1	3.59%	27753.85	6.11	tr|E5RHP7|E5RHP7_HUMAN Carbonic anhydrase 1 (Fragment) OS=Homo sapiens GN=CA1 PE=1 SV=1								
$102-7			1	1	5.14%	19236.32	6.75	tr|E5RH81|E5RH81_HUMAN Carbonic anhydrase 1 (Fragment) OS=Homo sapiens GN=CA1 PE=1 SV=6								
$102-8			1	1	10.34%	9406.94	9.57	tr|E5RJF6|E5RJF6_HUMAN Carbonic anhydrase 1 (Fragment) OS=Homo sapiens GN=CA1 PE=1 SV=1								
	R16062_1_KIAA,12	K.VLDALQAIK.T		971.176	-0.24	2	1		2.3457	0.3349	712.7	1	13|16	5.81	1	8

	436														
$103-1			1	1	1.04%	94973.05	5.7	sp|P02671|FIBA_HUMAN Fibrinogen alpha chain OS=Homo sapiens GN=FGA PE=1 SV=2							
$103-2			1	1	3.11%	33004.95	8.57	tr|A0A087WUA0|A0A087WUA0_HUMAN Fibrinogen alpha chain OS=Homo sapiens GN=FGA PE=1 SV=1							
$103-3			1	1	3.60%	28830.95	9.51	tr|Q3KRA7|Q3KRA7_HUMAN FGA protein (Fragment) OS=Homo sapiens GN=FGA PE=2 SV=1							
$103-4			1	1	3.11%	32990.92	8.57	tr|Q6NSD8|Q6NSD8_HUMAN FGA protein OS=Homo sapiens GN=FGA PE=2 SV=1							
	R16062_1_KIAA,8881	K.VQHIQLLQK.N		1107.3312	0.1682	2	1		2.2214	0.2981	849.9	1 13|16	8.73	1	4
$104-1			1	1	2.66%	66022.66	5.63	sp|Q07065|CKAP4_HUMAN Cytoskeleton-associated protein 4 OS=Homo sapiens GN=CKAP4 PE=1 SV=2							
$104-2			1	1	2.66%	66022.66	5.63	tr|A0A024RBH2|A0A024RBH2_HUMAN Cytoskeleton-associated protein 4, isoform CRA_c OS=Homo sapiens GN=CKAP4 PE=4 SV=1							
$104-3			1	1	2.67%	67818.45	9.34	tr|Q6NWZ1|Q6NWZ1_HUMAN CKAP4 protein (Fragment) OS=Homo sapiens GN=CKAP4 PE=2 SV=1							
$104-4			1	1	2.86%	62060.65	5.51	tr|Q8TB01|Q8TB01_HUMAN Similar to cytoskeleton-associated protein 4 (Fragment) OS=Homo sapiens PE=2 SV=1							
$104-5			1	1	3.07%	58150.98	5.15	tr|B3KVX6|B3KVX6_HUMAN cDNA FLJ41699 fis, clone HCHON2004776, highly similar to Homo sapiens cytoskeleton-associated protein 4 (CKAP4), mRNA OS=Homo sapiens PE=2 SV=1							
	R16062_1_KIAA,19815	K.VQSLQATFGTFESILR.S		1798.0326	0.2056	2	1		2.6441	0.5202	767	1 15|30	5.97	1	5
$105-1			1	1	1.67%	93313.74	7.27	sp|Q15063|POSTN_HUMAN Periostin OS=Homo sapiens GN=POSTN PE=1 SV=2							
$105-2			1	1	1.67%	93313.74	7.27	tr|A0A024RDS2|A0A024RDS2_HUMAN Periostin, osteoblast specific factor, isoform CRA_c OS=Homo sapiens GN=POSTN PE=4 SV=1							
$105-3			1	1	1.80%	87020.37	7.87	tr|A0A024RDT5|A0A024RDT5_HUMAN Periostin, osteoblast specific factor, isoform CRA_a OS=Homo sapiens GN=POSTN PE=4 SV=1							
$105-4			1	1	1.73%	90143.17	8.08	tr|B1ALD9|B1ALD9_HUMAN Periostin OS=Homo sapiens GN=POSTN PE=1 SV=1							
$105-5			1	1	5.05%	30478.67	5.1	tr|B7Z6G1|B7Z6G1_HUMAN cDNA FLJ50671, highly similar to Periostin OS=Homo sapiens PE=2 SV=1							
$105-6			1	1	1.79%	87271.75	8.1	tr|C0IMJ3|C0IMJ3_HUMAN Periostin isoform thy6 OS=Homo sapiens PE=2 SV=1							
	R16062_1_KIAA,18	R.AAAITSDILEALGR.D		1401.5909	0.3439	2	1		2.6456	0.3147	1213.2	1 17|26	4.37	1	6

	169															
$106-1			1	1	6.19%	26145.3	7.7	sp|P36543|VATE1_HUMAN V-type proton ATPase subunit E 1 OS=Homo sapiens GN=ATP6V1E1 PE=1 SV=1								
$106-2			1	1	6.19%	26145.3	7.7	tr|Q53Y06|Q53Y06_HUMAN ATPase, H+ transporting, lysosomal 31kDa, V1 subunit E isoform 1 OS=Homo sapiens GN=ATP6V1E1 PE=2 SV=1								
$106-3			1	1	6.90%	23539.37	9.11	tr|C9J8H1|C9J8H1_HUMAN V-type proton ATPase subunit E 1 (Fragment) OS=Homo sapiens GN=ATP6V1E1 PE=1 SV=1								
	R16062_1_KIAA,17445	R.ARDDLITDLLNEAK.Q		1587.757	0.475	2	1		2.353	0.3593	812.2	1	14|26	4.23	1	3
$107-1			1	1	2.63%	47607.85	5.61	sp|Q9P1U1|ARP3B_HUMAN Actin-related protein 3B OS=Homo sapiens GN=ACTR3B PE=2 SV=1								
$107-2			1	1	5.24%	23712.34	5.36	sp|Q9C0K3|ARP3C_HUMAN Actin-related protein 3C OS=Homo sapiens GN=ACTR3C PE=2 SV=1								
$107-3			1	1	2.63%	47371.29	5.61	sp|P61158|ARP3_HUMAN Actin-related protein 3 OS=Homo sapiens GN=ACTR3 PE=1 SV=3								
$107-4			1	1	3.00%	42004.07	5.41	tr|B4DXW1|B4DXW1_HUMAN Actin-related protein 3 OS=Homo sapiens GN=ACTR3 PE=1 SV=1								
$107-5			1	1	2.63%	47371.29	5.61	tr|A0A024RAI1|A0A024RAI1_HUMAN ARP3 actin-related protein 3 homolog (Yeast), isoform CRA_a OS=Homo sapiens GN=ACTR3 PE=3 SV=1								
$107-6			1	1	3.81%	32656.46	6.67	tr|B4DTI0|B4DTI0_HUMAN cDNA FLJ52521, highly similar to Actin-like protein 3 OS=Homo sapiens PE=2 SV=1								
$107-7			1	1	5.82%	21312.6	5.12	tr|A0A0A0MTI9|A0A0A0MTI9_HUMAN Actin-related protein 3C OS=Homo sapiens GN=ACTR3C PE=3 SV=1								
$107-8			1	1	3.90%	32114.05	8.42	tr|Q59GD5|Q59GD5_HUMAN Actin-related protein 3-beta variant (Fragment) OS=Homo sapiens PE=2 SV=1								
$107-9			1	1	5.29%	23770.31	5.6	tr|H7C4J1|H7C4J1_HUMAN Actin-related protein 3C (Fragment) OS=Homo sapiens GN=ACTR3C PE=4 SV=1								
$107-10			1	1	4.95%	24320.08	8.41	tr|C9IZN3|C9IZN3_HUMAN Actin-related protein 3C (Fragment) OS=Homo sapiens GN=ACTR3C PE=4 SV=1								
$107-11			1	1	2.98%	42258.4	5.59	tr|Q59FV6|Q59FV6_HUMAN ARP3 actin-related protein 3 homolog variant (Fragment) OS=Homo sapiens PE=2 SV=1								
$107-12			1	1	3.33%	37871.7	5.97	tr|B7Z9W3|B7Z9W3_HUMAN cDNA, FLJ78977, highly similar to Homo sapiens ARP3 actin-related protein 3 homolog B (yeast) (ACTR3B), mRNA OS=Homo sapiens PE=2 SV=1								
	R16062_1_KIAA,21822	R.DITYFIQQLLR.E		1410.6426	-0.9924	2	1		2.2329	0.3124	384.2	3	11|20	5.84	1	12
$108-1			1	1	5.09%	30515.12	6.62	sp|Q15661|TRYB1_HUMAN Tryptase alpha/beta-1 OS=Homo sapiens GN=TPSAB1 PE=1 SV=1								
$108-			1	1	5.09%	30515.12	6.62	sp|P20231|TRYB2_HUMAN Tryptase beta-2 OS=Homo sapiens								

2								GN=TPSB2 PE=1 SV=2							
$108-3			1	1	4.96%	31282.12	6.62	tr|J3QTS8|J3QTS8_HUMAN Tryptase alpha/beta-1 OS=Homo sapiens GN=TPSAB1 PE=1 SV=1							
$108-4			1	1	4.96%	31296.19	6.85	tr|Q6NZY1|Q6NZY1_HUMAN TPSB2 protein OS=Homo sapiens GN=TPSB2 PE=2 SV=1							
$108-5			1	1	5.09%	30488.1	6.62	tr|Q6B051|Q6B051_HUMAN Tryptase alpha/beta 1 OS=Homo sapiens GN=TPSAB1 PE=2 SV=1							
$108-6			1	1	5.09%	30713.46	6.18	tr|Q86TM8|Q86TM8_HUMAN TPSAB1 protein OS=Homo sapiens GN=TPSAB1 PE=2 SV=1							
$108-7			1	1	4.96%	31418.28	6.82	tr|A0A087WUI4|A0A087WUI4_HUMAN Tryptase beta-2 OS=Homo sapiens GN=TPSB2 PE=1 SV=1							
$108-8			1	1	4.79%	32576.47	6.82	tr|A0A087X1U0|A0A087X1U0_HUMAN Tryptase beta-2 OS=Homo sapiens GN=TPSB2 PE=1 SV=1							
$108-9			1	1	5.09%	30517.08	6.62	tr|Q6FHB8|Q6FHB8_HUMAN TPSB2 protein (Fragment) OS=Homo sapiens GN=TPSB2 PE=2 SV=1							
$108-10			1	1	5.09%	30491.1	6.58	tr|Q96RZ6|Q96RZ6_HUMAN Tryptase I OS=Homo sapiens GN=tryptaseC PE=3 SV=1							
$108-11			1	1	5.07%	30869.65	6.37	tr|Q86UA5|Q86UA5_HUMAN TPSAB1 protein (Fragment) OS=Homo sapiens GN=TPSAB1 PE=2 SV=1							
$108-12			1	1	6.57%	23895.27	5.96	tr|A0A0C4DGM1|A0A0C4DGM1_HUMAN Tryptase alpha/beta-1 (Fragment) OS=Homo sapiens GN=TPSAB1 PE=1 SV=1							
	R16062_1_KIAA,6796	R.DSCQGDSGGPLVCK.V		1480.5471	0.5371	2	1		2.5367	0.3999	756.9	1 16|26	4.21	1	12
$109-1			1	1	3.14%	54305.86	5.55	sp|P04004|VTNC_HUMAN Vitronectin OS=Homo sapiens GN=VTN PE=1 SV=1							
$109-2			1	1	3.14%	54305.86	5.55	tr|D9ZGG2|D9ZGG2_HUMAN Vitronectin OS=Homo sapiens GN=VTN PE=4 SV=1							
$109-3			1	1	7.01%	23605.07	4.53	tr|B7Z553|B7Z553_HUMAN cDNA FLJ51266, highly similar to Vitronectin OS=Homo sapiens PE=2 SV=1							
	R16062_1_KIAA,18194	R.DVWGIEGPIDAAFTR.I		1647.8126	0.4856	2	1		2.2114	0.2747	466.4	1 14|28	4.03	1	3
$110-1			1	1	2.97%	75873.42	5.42	sp|P08133|ANXA6_HUMAN Annexin A6 OS=Homo sapiens GN=ANXA6 PE=1 SV=3							
$110-2			1	1	3.66%	61852.41	5.36	tr|B7Z582|B7Z582_HUMAN Annexin OS=Homo sapiens PE=2 SV=1							
$110-3			1	1	6.06%	36962.98	5.62	tr|E7EMC6|E7EMC6_HUMAN Annexin OS=Homo sapiens GN=ANXA6 PE=1 SV=1							
$110-4			1	1	6.06%	36998.98	5.69	tr|Q6ZP35|Q6ZP35_HUMAN Annexin OS=Homo sapiens PE=2 SV=1							
	R16062_1_KIAA,16773	R.EDAQVAAEILEIADTPSGDK.T		2073.2011	0.8011	2	1		2.606	0.4502	972.6	1 21|38	3.71	1	4

$111-1			1	1	3.68%	29804.17	5.57	sp|P35232|PHB_HUMAN Prohibitin OS=Homo sapiens GN=PHB PE=1 SV=1							
$111-2			1	1	3.68%	29820.21	5.57	tr|Q6PUJ7|Q6PUJ7_HUMAN Epididymis luminal protein 215 OS=Homo sapiens GN=HEL-S-54e PE=2 SV=1							
$111-3			1	1	3.68%	29804.17	5.57	tr|A8K401|A8K401_HUMAN Prohibitin, isoform CRA_a OS=Homo sapiens GN=PHB PE=2 SV=1							
$111-4			1	1	3.68%	29803.22	6.02	tr|Q53FV0|Q53FV0_HUMAN Prohibitin variant (Fragment) OS=Homo sapiens PE=2 SV=1							
$111-5			1	1	6.41%	16919.67	5.36	tr|Q9BSE8|Q9BSE8_HUMAN ZNF607 protein OS=Homo sapiens GN=ZNF607 PE=2 SV=1							
$111-6			1	1	4.63%	23824.35	5.98	tr|E9PCW0|E9PCW0_HUMAN Prohibitin (Fragment) OS=Homo sapiens GN=PHB PE=1 SV=2							
$111-7			1	1	3.68%	29832.22	5.57	tr|Q6FHP5|Q6FHP5_HUMAN PHB protein (Fragment) OS=Homo sapiens GN=PHB PE=2 SV=1							
$111-8			1	1	4.95%	22398.74	5.97	tr|E7ESE2|E7ESE2_HUMAN Prohibitin (Fragment) OS=Homo sapiens GN=PHB PE=1 SV=2							
$111-9			1	1	4.98%	22270.57	5.71	tr|C9JZ20|C9JZ20_HUMAN Prohibitin (Fragment) OS=Homo sapiens GN=PHB PE=1 SV=1							
$111-10			1	1	4.07%	26891.77	5.29	tr|C9JW96|C9JW96_HUMAN Prohibitin (Fragment) OS=Homo sapiens GN=PHB PE=1 SV=2							
	R16062_1_KIAA,12251	R.FDAGELITQR.E		1150.2665	0.7325	2	1		2.9312	0.2057	1223.3	1 14|18	4.37	1	10
$112-1			1	1	7.98%	23277.28	9.97	sp|P48047|ATPO_HUMAN ATP synthase subunit O, mitochondrial OS=Homo sapiens GN=ATP5O PE=1 SV=1							
$112-2			1	1	7.98%	23261.24	9.97	tr|Q53HH2|Q53HH2_HUMAN Mitochondrial ATP synthase, O subunit variant (Fragment) OS=Homo sapiens PE=2 SV=1							
$112-3			1	1	7.02%	26798.86	9.51	tr|H7C0C1|H7C0C1_HUMAN Uncharacterized protein (Fragment) OS=Homo sapiens PE=4 SV=2							
	R16062_1_KIAA,19924	R.FSPLTTNLINLLAENGR.L		1874.1306	0.6936	2	1		2.2394	0.432	378.1	1 15|32	6	1	3
$113-1			1	1	6.02%	24393.51	6.12	sp|P62491|RB11A_HUMAN Ras-related protein Rab-11A OS=Homo sapiens GN=RAB11A PE=1 SV=3							
$113-2			1	1	5.96%	24488.49	5.64	sp|Q15907|RB11B_HUMAN Ras-related protein Rab-11B OS=Homo sapiens GN=RAB11B PE=1 SV=4							
$113-3			1	1	6.02%	24393.51	6.12	tr|A0A024R5Z8|A0A024R5Z8_HUMAN RAB11A, member RAS oncogene family, isoform CRA_a OS=Homo sapiens GN=RAB11A PE=3 SV=1							
$113-4			1	1	4.98%	28861.65	8.46	tr|B4DMN1|B4DMN1_HUMAN cDNA FLJ61136, highly similar to Ras-related protein Rab-11A OS=Homo sapiens PE=2 SV=1							
$113-5			1	1	8.39%	17668.03	7.93	tr|H3BMH2|H3BMH2_HUMAN Ras-related protein Rab-11A (Fragment) OS=Homo sapiens GN=RAB11A PE=3 SV=1							

$113-6			1	1	6.57%	22486.45	6.91	tr|H3BSC1|H3BSC1_HUMAN Ras-related protein Rab-11A OS=Homo sapiens GN=RAB11A PE=1 SV=1							
	R16062_1_KIAA,16247	R.GAVGALLVYDIAK.H		1290.5333	0.7963	2	1		2.5586	0.4198	662.4	1 13|24	5.84	1	6
$114-1			1	1	5.78%	30374.91	8.55	sp|P16152|CBR1_HUMAN Carbonyl reductase [NADPH] 1 OS=Homo sapiens GN=CBR1 PE=1 SV=3							
$114-2			1	1	5.78%	30850.27	5.82	sp|O75828|CBR3_HUMAN Carbonyl reductase [NADPH] 3 OS=Homo sapiens GN=CBR3 PE=1 SV=3							
$114-3			1	1	8.99%	19021.89	5.87	tr|E9PQ63|E9PQ63_HUMAN Carbonyl reductase [NADPH] 1 OS=Homo sapiens GN=CBR1 PE=1 SV=1							
$114-4			1	1	7.21%	24498.77	9.06	tr|A8MTM1|A8MTM1_HUMAN Carbonyl reductase [NADPH] 1 OS=Homo sapiens GN=CBR1 PE=1 SV=1							
$114-5			1	1	5.78%	30850.27	5.82	tr|V9HW40|V9HW40_HUMAN Epididymis secretory protein Li 25 OS=Homo sapiens GN=HEL-S-25 PE=1 SV=1							
$114-6			1	1	5.78%	30868.3	5.82	tr|Q53F60|Q53F60_HUMAN Carbonyl reductase 3 variant (Fragment) OS=Homo sapiens PE=2 SV=1							
	R16062_1_KIAA,10798	R.GQAAVQQLQAEGLSPR.F		1653.8219	0.4789	2	1		2.4342	0.3702	804.1	1 15|30	6	1	6
$115-1			1	1	0.55%	269767.09	5.77	sp|Q9Y490|TLN1_HUMAN Talin-1 OS=Homo sapiens GN=TLN1 PE=1 SV=3							
	R16062_1_KIAA,14868	R.ILAQATSDLVNAIK.A		1457.6976	1.1106	2	1		2.2411	0.4269	275	7 11|26	5.84	1	1
$116-1			1	1	3.64%	59971.64	8.96	sp|Q9NSA0|S22AB_HUMAN Solute carrier family 22 member 11 OS=Homo sapiens GN=SLC22A11 PE=1 SV=1							
$116-2			1	1	4.16%	52617.81	9.21	tr|A6NCG2|A6NCG2_HUMAN Solute carrier family 22 member 11 OS=Homo sapiens GN=SLC22A11 PE=1 SV=1							
$116-3			1	1	8.06%	27139.52	10.72	tr|Q8NBZ3|Q8NBZ3_HUMAN cDNA FLJ90646 fis, clone PLACE1004279 OS=Homo sapiens PE=2 SV=1							
	R16062_1_KIAA,13618	R.INGHKEAKNLTIEVLMSSVK.E		2212.5993	1.2373	2	1		2.3195	0.1498	360.2	1 14|38	8.51	1	3
$117-1			1	1	3.52%	32708.73	4.69	sp|P09493|TPM1_HUMAN Tropomyosin alpha-1 chain OS=Homo sapiens GN=TPM1 PE=1 SV=2							
$117-2			1	1	3.52%	32850.87	4.66	sp|P07951|TPM2_HUMAN Tropomyosin beta chain OS=Homo sapiens GN=TPM2 PE=1 SV=1							
$117-3			1	1	3.51%	32950.14	4.68	sp|P06753|TPM3_HUMAN Tropomyosin alpha-3 chain OS=Homo sapiens GN=TPM3 PE=1 SV=2							
$117-4			1	1	4.03%	28521.96	4.67	sp|P67936|TPM4_HUMAN Tropomyosin alpha-4 chain OS=Homo sapiens GN=TPM4 PE=1 SV=3							
$117-5			1	1	3.52%	32876.07	4.72	tr|D9YZV5|D9YZV5_HUMAN Tropomyosin 1 (Alpha) isoform 4 OS=Homo sapiens GN=TPM1 PE=3 SV=1							
$117-			1	1	4.03%	28608.04	4.77	tr|B7Z722|B7Z722_HUMAN Tropomyosin 1 (Alpha), isoform							

6						CRA_i OS=Homo sapiens GN=TPM1 PE=2 SV=1
$117-7	1	1	4.03%	28747.31	4.78	tr|H7BYY1|H7BYY1_HUMAN Tropomyosin 1 (Alpha), isoform CRA_m OS=Homo sapiens GN=TPM1 PE=1 SV=1
$117-8	1	1	3.64%	31753.14	4.85	tr|B7Z596|B7Z596_HUMAN Tropomyosin alpha-1 chain OS=Homo sapiens GN=TPM1 PE=1 SV=1
$117-9	1	1	4.03%	28521.96	4.67	tr|V9HW56|V9HW56_HUMAN Epididymis secretory protein Li 108 OS=Homo sapiens GN=HEL-S-108 PE=2 SV=1
$117-10	1	1	3.07%	37453.09	4.67	tr|Q6ZN40|Q6ZN40_HUMAN Tropomyosin 1 (Alpha), isoform CRA_f OS=Homo sapiens GN=TPM1 PE=1 SV=1
$117-11	1	1	4.31%	27175.7	4.71	tr|Q5HYB6|Q5HYB6_HUMAN Epididymis luminal protein 189 OS=Homo sapiens GN=DKFZp686J1372 PE=1 SV=1
$117-12	1	1	3.52%	32866.03	4.69	tr|A0A0K0K1I0|A0A0K0K1I0_HUMAN Epididymis secretory protein Li 265 OS=Homo sapiens GN=HEL-S-265 PE=2 SV=1
$117-13	1	1	3.30%	34402.47	5.35	tr|Q59GR8|Q59GR8_HUMAN TPM1 protein variant (Fragment) OS=Homo sapiens PE=2 SV=1
$117-14	1	1	5.59%	20621.09	4.61	tr|K7ENT6|K7ENT6_HUMAN Tropomyosin alpha-4 chain (Fragment) OS=Homo sapiens GN=TPM4 PE=1 SV=1
$117-15	1	1	3.52%	32677.74	4.7	tr|A0A024R5W6|A0A024R5W6_HUMAN Tropomyosin 1 (Alpha), isoform CRA_a OS=Homo sapiens GN=TPM1 PE=3 SV=1
$117-16	1	1	3.52%	33026.07	4.65	tr|A7XZE4|A7XZE4_HUMAN Beta tropomyosin isoform OS=Homo sapiens GN=TPM2b PE=2 SV=1
$117-17	1	1	10.53%	11017.31	4.8	tr|D6R904|D6R904_HUMAN Tropomyosin alpha-3 chain OS=Homo sapiens GN=TPM3 PE=1 SV=1
$117-18	1	1	3.51%	33222.68	4.73	tr|J3KN67|J3KN67_HUMAN Tropomyosin alpha-3 chain OS=Homo sapiens GN=TPM3 PE=1 SV=1
$117-19	1	1	5.88%	19306.65	4.76	tr|K7ERG3|K7ERG3_HUMAN Tropomyosin alpha-4 chain (Fragment) OS=Homo sapiens GN=TPM4 PE=1 SV=1
$117-20	1	1	4.03%	28579.97	4.74	tr|H0YK48|H0YK48_HUMAN Tropomyosin alpha-1 chain OS=Homo sapiens GN=TPM1 PE=1 SV=1
$117-21	1	1	6.33%	17833.02	4.8	tr|B4DTK3|B4DTK3_HUMAN cDNA FLJ57891, highly similar to Tropomyosin beta chain OS=Homo sapiens PE=2 SV=1
$117-22	1	1	14.71%	8014.11	4.82	tr|H0YL80|H0YL80_HUMAN Tropomyosin alpha-1 chain (Fragment) OS=Homo sapiens GN=TPM1 PE=1 SV=1
$117-23	1	1	1.38%	82419.73	4.9	tr|M1VPF4|M1VPF4_HUMAN Tyrosine-protein kinase receptor OS=Homo sapiens GN=TPM3-ROS1 PE=2 SV=1
$117-24	1	1	4.08%	28385.68	4.74	tr|D9YZV7|D9YZV7_HUMAN Tropomyosin 1 (Alpha) isoform 6 OS=Homo sapiens GN=TPM1 PE=3 SV=1
$117-25	1	1	5.32%	21833.55	4.78	tr|B4DGC2|B4DGC2_HUMAN cDNA FLJ56690, highly similar to Tropomyosin beta chain OS=Homo sapiens PE=2 SV=1
$117-26	1	1	4.12%	28420.27	4.89	tr|Q15657|Q15657_HUMAN Tropomyosin isoform OS=Homo sapiens PE=2 SV=1

$117-27			1	1	3.11%	36747.29	4.73	tr|Q5TCU8|Q5TCU8_HUMAN Tropomyosin beta chain OS=Homo sapiens GN=TPM2 PE=1 SV=1							
$117-28			1	1	4.41%	26420.88	4.75	tr|A0A087WWU8|A0A087WWU8_HUMAN Tropomyosin alpha-3 chain OS=Homo sapiens GN=TPM3 PE=1 SV=1							
$117-29			1	1	3.52%	32989.97	4.63	tr|V9HW25|V9HW25_HUMAN Epididymis secretory protein Li 273 OS=Homo sapiens GN=HEL-S-273 PE=2 SV=1							
$117-30			1	1	4.93%	23754.64	4.72	tr|Q8TCG4|Q8TCG4_HUMAN TPMsk1 (Fragment) OS=Homo sapiens GN=TPM1 PE=2 SV=1							
$117-31			1	1	4.48%	25470.49	4.6	tr|H0YNC7|H0YNC7_HUMAN Tropomyosin alpha-1 chain (Fragment) OS=Homo sapiens GN=TPM1 PE=1 SV=1							
$117-32			1	1	4.08%	28508.91	4.75	tr|Q1ZYL5|Q1ZYL5_HUMAN Tropomyosin 1 alpha variant 6 OS=Homo sapiens PE=2 SV=1							
$117-33			1	1	4.08%	28524.95	4.75	tr|F5H7S3|F5H7S3_HUMAN Tropomyosin alpha-1 chain OS=Homo sapiens GN=TPM1 PE=1 SV=2							
$117-34			1	1	3.77%	30380.92	4.64	tr|H0YL52|H0YL52_HUMAN Tropomyosin alpha-1 chain (Fragment) OS=Homo sapiens GN=TPM1 PE=1 SV=1							
$117-35			1	1	4.37%	26221.37	4.64	tr|B4DVY2|B4DVY2_HUMAN cDNA FLJ54184, highly similar to Tropomyosin alpha-4 chain OS=Homo sapiens PE=2 SV=1							
$117-36			1	1	7.25%	15849.09	5.01	tr|K7EP68|K7EP68_HUMAN Tropomyosin alpha-4 chain (Fragment) OS=Homo sapiens GN=TPM4 PE=1 SV=1							
$117-37			1	1	4.03%	29017.81	4.72	tr|B2RDE1|B2RDE1_HUMAN cDNA, FLJ96568, highly similar to Homo sapiens tropomyosin 3 (TPM3), mRNA OS=Homo sapiens PE=2 SV=1							
$117-38			1	1	3.52%	32708.73	4.69	tr|D9YZV4|D9YZV4_HUMAN Tropomyosin 1 (Alpha) isoform 1 OS=Homo sapiens GN=TPM1 PE=3 SV=1							
$117-39			1	1	3.52%	32814.77	4.64	tr|Q5TCU3|Q5TCU3_HUMAN Tropomyosin beta chain OS=Homo sapiens GN=TPM2 PE=1 SV=1							
$117-40			1	1	6.49%	17634.69	4.71	tr|B4DTB1|B4DTB1_HUMAN cDNA FLJ52936, weakly similar to Tropomyosin alpha-4 chain OS=Homo sapiens PE=2 SV=1							
$117-41			1	1	4.61%	24883.93	4.66	tr|C9IZA2|C9IZA2_HUMAN cDNA FLJ26372 fis, clone HRT06233 OS=Homo sapiens PE=2 SV=2							
$117-42			1	1	7.04%	16365.49	4.7	tr|H0YKX5|H0YKX5_HUMAN Tropomyosin alpha-1 chain (Fragment) OS=Homo sapiens GN=TPM1 PE=1 SV=1							
$117-43			1	1	6.37%	17904	5.02	tr|H0YKP3|H0YKP3_HUMAN Tropomyosin alpha-1 chain OS=Homo sapiens GN=TPM1 PE=1 SV=1							
	R16062_1_KIAA,12135	R.IQLVEEELDR.A		1244.3766	0.1806	2	1		2.4985	0.3229	783	1 13|18	4	1	43
$118-1			1	1	0.68%	193540.03	5.93	sp|A4D0S4|LAMB4_HUMAN Laminin subunit beta-4 OS=Homo sapiens GN=LAMB4 PE=2 SV=1							
$118-2			1	1	0.68%	193570.05	5.93	tr|B7ZMJ6|B7ZMJ6_HUMAN Laminin, beta 4 OS=Homo sapiens GN=LAMB4 PE=2 SV=1							

	R16062_1_KIAA,12069	R.KIQDLNLSRQAK.A		1414.636	-0.68	2	1		2.811	0.1702	284.6	5	12|22	9.99	1	2
$119-1			1	1	1.36%	74139.71	6.57	sp|P02545|LMNA_HUMAN Prelamin-A/C OS=Homo sapiens GN=LMNA PE=1 SV=1								
$119-2			1	1	1.85%	55638.35	6.24	tr|Q3BDU5|Q3BDU5_HUMAN Prelamin-A/C OS=Homo sapiens GN=LMNA PE=1 SV=1								
$119-3			1	1	1.94%	53197.58	6.03	tr|Q8N519|Q8N519_HUMAN LMNA protein OS=Homo sapiens PE=2 SV=1								
$119-4			1	1	4.95%	20796.48	6.04	tr|H0YAB0|H0YAB0_HUMAN Prelamin-A/C (Fragment) OS=Homo sapiens GN=LMNA PE=1 SV=1								
$119-5			1	1	1.88%	55043.76	6.51	tr|Q5I6Y5|Q5I6Y5_HUMAN Lamin A/C transcript variant 1 OS=Homo sapiens GN=LMNA PE=2 SV=1								
$119-6			1	1	1.57%	65116.95	6.4	tr|W8QEH3|W8QEH3_HUMAN Lamin A/C OS=Homo sapiens GN=LMNA PE=3 SV=1								
$119-7			1	1	1.83%	55762.49	6.55	tr|Q5TCI8|Q5TCI8_HUMAN Prelamin-A/C OS=Homo sapiens GN=LMNA PE=1 SV=1								
	R16062_1_KIAA,11764	R.LADALQELR.A		1029.1724	1.0134	2	1		2.8909	0.2881	1200.8	1	15|16	4.37	1	7
$120-1			1	1	7.84%	35619.67	8.42	sp|Q9H7P6|MB12B_HUMAN Multivesicular body subunit 12B OS=Homo sapiens GN=MVB12B PE=1 SV=2								
$120-2			1	1	7.84%	35619.67	8.42	tr|A0A024R8B8|A0A024R8B8_HUMAN Chromosome 9 open reading frame 28, isoform CRA_a OS=Homo sapiens GN=C9orf28 PE=4 SV=1								
$120-3			1	1	8.22%	33830.34	5.69	tr|B7Z1P9|B7Z1P9_HUMAN cDNA FLJ53722 OS=Homo sapiens PE=2 SV=1								
$120-4			1	1	11.68%	24052.64	9.69	tr|B7Z4X0|B7Z4X0_HUMAN cDNA FLJ55114 OS=Homo sapiens PE=2 SV=1								
$120-5			1	1	13.30%	21225.17	8.72	tr|Q9H7N7|Q9H7N7_HUMAN FLJ00031 protein (Fragment) OS=Homo sapiens GN=FLJ00031 PE=2 SV=1								
	R16062_1_KIAA,15795	R.NHDSSQPTTPSQSSAASTPAPNLPR.H		2549.6528	0.5638	2	1		2.3006	0.1551	191.5	3	12|48	6.74	1	5
$121-1			1	1	2.06%	66248.64	8.22	sp|Q5JTV8|TOIP1_HUMAN Torsin-1A-interacting protein 1 OS=Homo sapiens GN=TOR1AIP1 PE=1 SV=2								
$121-2			1	1	4.05%	33639.61	7.9	tr|H0Y4R4|H0Y4R4_HUMAN Torsin-1A-interacting protein 1 (Fragment) OS=Homo sapiens GN=TOR1AIP1 PE=1 SV=1								
$121-3			1	1	2.60%	52406.24	6.59	tr|A0A0A0MSK5|A0A0A0MSK5_HUMAN Torsin-1A-interacting protein 1 OS=Homo sapiens GN=TOR1AIP1 PE=1 SV=1								
$121-4			1	1	2.00%	67822.29	7.71	tr|J3KN66|J3KN66_HUMAN Torsin-1A-interacting protein 1 OS=Homo sapiens GN=TOR1AIP1 PE=1 SV=1								
	R16062_1_KIAA,15553	R.SQPAILLLTAAR.D		1254.5042	-0.3248	2	1		2.3921	0.3108	558.3	1	15|22	9.47	1	4

$122-1			1	1	17.48%	11536.25	6.19	tr|Q562M3|Q562M3_HUMAN Actin-like protein (Fragment) OS=Homo sapiens GN=ACT PE=3 SV=1							
	R16062_1_KIAA,12304	R.VAPEEHPVLLTQAPLNPK.A		1954.2589	0.3749	2	1		2.2019	0.1597	420.2	1 16|34	5.4	1	1
$123-1			1	1	1.35%	103057.69	5.25	sp|P12814|ACTN1_HUMAN Alpha-actinin-1 OS=Homo sapiens GN=ACTN1 PE=1 SV=2							
$123-2			1	1	1.32%	104854.22	5.27	sp|O43707|ACTN4_HUMAN Alpha-actinin-4 OS=Homo sapiens GN=ACTN4 PE=1 SV=2							
$123-3			1	1	1.35%	103057.69	5.25	tr|A0A024R694|A0A024R694_HUMAN Actinin, alpha 1, isoform CRA_a OS=Homo sapiens GN=ACTN1 PE=4 SV=1							
$123-4			1	1	2.25%	61575.38	5.05	tr|B4DFY0|B4DFY0_HUMAN cDNA FLJ53313, highly similar to Alpha-actinin-1 OS=Homo sapiens PE=2 SV=1							
$123-5			1	1	1.46%	94783.8	5.47	tr|B7Z565|B7Z565_HUMAN cDNA FLJ54739, highly similar to Alpha-actinin-1 OS=Homo sapiens PE=2 SV=1							
$123-6			1	1	13.95%	10195.33	5.41	tr|Q5ZEZ4|Q5ZEZ4_HUMAN Alpha-actinin 1 (Fragment) OS=Homo sapiens GN=ACTN1 PE=2 SV=1							
$123-7			1	1	4.04%	34320.38	5.14	tr|H7C5W8|H7C5W8_HUMAN Alpha-actinin-1 (Fragment) OS=Homo sapiens GN=ACTN1 PE=1 SV=1							
$123-8			1	1	4.78%	28762.32	4.94	tr|H0YJW3|H0YJW3_HUMAN Alpha-actinin-1 (Fragment) OS=Homo sapiens GN=ACTN1 PE=1 SV=1							
$123-9			1	1	1.46%	94825.88	5.47	tr|H9KV75|H9KV75_HUMAN Alpha-actinin-1 OS=Homo sapiens GN=ACTN1 PE=1 SV=1							
$123-10			1	1	1.89%	73619.77	5.16	tr|Q96BG6|Q96BG6_HUMAN ACTN4 protein (Fragment) OS=Homo sapiens GN=ACTN4 PE=2 SV=2							
$123-11			1	1	5.80%	24263.23	5.37	tr|H0YJ11|H0YJ11_HUMAN Alpha-actinin-1 (Fragment) OS=Homo sapiens GN=ACTN1 PE=1 SV=1							
$123-12			1	1	2.30%	59564.47	4.82	tr|F5GXS2|F5GXS2_HUMAN Alpha-actinin-4 OS=Homo sapiens GN=ACTN4 PE=1 SV=2							
$123-13			1	1	3.51%	39001.7	5.1	tr|H7C144|H7C144_HUMAN Alpha-actinin-4 (Fragment) OS=Homo sapiens GN=ACTN4 PE=1 SV=1							
$123-14			1	1	2.54%	54281.13	4.98	tr|B3KUX9|B3KUX9_HUMAN cDNA FLJ40884 fis, clone UTERU2000607, highly similar to Alpha-actinin-1 OS=Homo sapiens PE=2 SV=1							
$123-15			1	1	3.88%	35411.97	5.01	tr|A1L0V1|A1L0V1_HUMAN ACTN1 protein (Fragment) OS=Homo sapiens GN=ACTN1 PE=2 SV=1							
$123-16			1	1	2.01%	67142.84	5.83	tr|B7Z2W3|B7Z2W3_HUMAN cDNA FLJ54432, highly similar to Alpha-actinin-1 OS=Homo sapiens PE=2 SV=1							
	R16062_1_KIAA,19609	R.VGWEQLLTTIAR.T		1387.6091	0.0821	2	1		2.4487	0.3763	707.1	1 13|22	5.97	1	16
$124-1			1	1	7.28%	28048.49	8.25	sp|P35270|SPRE_HUMAN Sepiapterin reductase OS=Homo sapiens GN=SPR PE=1 SV=1							

$124-2			1	1	15.45%	13013.2	11	tr|B4DSF0|B4DSF0_HUMAN cDNA FLJ56734, moderately similar to Sepiapterin reductase (EC 1.1.1.153) OS=Homo sapiens PE=2 SV=1								
	R16062_1_KIAA,20222	R.VPADLGAEAGLQQLLGALR.E		1893.1771	0.6201	2	1		3.4054	0.5128	1101.3	1	20|36	4.37	1	2
